# Asymptotics of the Determinant of Discrete Laplacians on Triangulated and Quadrangulated Surfaces

**DOI:** 10.1007/s00220-022-04437-3

**Published:** 2022-07-30

**Authors:** Konstantin Izyurov, Mikhail Khristoforov

**Affiliations:** 1 University of Helsinki, Helsinki, Finland; 2 Chebyshev Laboratory, Saint Petersburg State University, Saint Petersburg, Russia

## Abstract

Consider a surface $\Omega$ with a boundary obtained by gluing together a finite number of equilateral triangles, or squares, along their boundaries, equipped with a vector bundle with a flat unitary connection. Let $\Omega ^{\delta }$ be a discretization of this surface, in which each triangle or square is discretized by a bi-periodic lattice of mesh size $\delta$, possessing enough symmetries so that these discretizations can be glued together seamlessly. We show that the logarithm of the product of non-zero eigenvalues of the discrete Laplacian acting on the sections of the bundle is asymptotic to

$$\begin{aligned} A|\Omega ^{\delta }|+B|\partial \Omega ^{\delta }|+C\log \delta +D+o(1). \end{aligned}$$

Here *A* and *B* are constants that depend only on the lattice, *C* is an explicit constant depending on the bundle, the angles at conical singularities and at corners of the boundary, and *D* is a sum of lattice-dependent contributions from singularities and a universal term that can be interpreted as a zeta-regularization of the determinant of the continuum Laplacian acting on the sections of the bundle. We allow for Dirichlet or Neumann boundary conditions, or mixtures thereof. Our proof is based on an integral formula for the determinant in terms of theta function, and the functional Central limit theorem.

## Introduction

Let $\Omega$ be a connected surface, possibly with boundary, obtained by gluing finitely many equal equilateral triangles, or squares, along their boundaries. Thus, $\Omega$ may have conical singularities and piece-wise straight boundary with corners; the cone and wedge angles either all belong to $\frac{\pi }{3}k,$ or to $\frac{\pi }{2}k,$ $k\in \mathbb {N}$; we refer to the former situation as a triangulation and the latter one as a quadrangulation. The boundary of $\Omega$ will be decomposed into two parts, $\partial _{\mathcal {D}}\Omega$ and $\partial _{\mathcal {N}}\Omega ,$ that will carry Dirichlet and Neumann boundary conditions respectively; we assume that there are finitely many points separating the two. We assume that $\Omega$ is equipped with a finite rank vector bundle with a unitary flat connection $\varphi$. We furthermore allow $\Omega$ to have finitely many punctures, distinct from the set of conical singularities, and allow $\varphi$ to have monodromy around those punctures. Our main results and techniques are new already in the case of a trivial line bundle, and the reader interested in the simplest situation may think of this case.

**Fig. 1:**
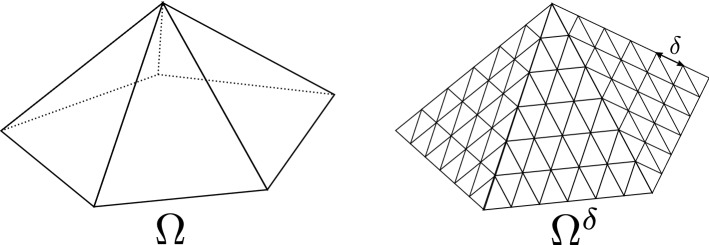
An example of a surface $\Omega ,$ glued of five equilateral triangles, and its discretization $\Omega ^{\delta }$ by triangular lattice. In this case, $\Omega$ has one conical singularity of angle $\frac{5\pi }{3}$ and five boundary corners of angle $\frac{2\pi }{3}.$ Note that the discrete triangles are glued so that the local graph structure at the edges is no different from that in the bulk of the triangles

We summarize our setup briefly here, referring to Sect. 2 for the detailed definitions. By a *lattice*, we mean an (infinite) undirected planar graph with non-negative weights on edges embedded bi-periodically in the plane $\mathbb {C}$. Given a lattice which is symmetric under reflections $z\mapsto {\overline{z}}$ and under rotation by $\frac{\pi }{2}$ or $\frac{\pi }{3}$ (such as, e.g., the square lattice in the case of quadrangulations and the triangular lattice in the case of triangulations), we can discretize $\Omega$ by this lattice scaled to have small mesh $\delta$. We denote the discretized surface by $\Omega ^{\delta }$, see Fig. 1. In what follows, by a *lattice-dependent* constant, we mean a quantity that does not depend on $\delta$, but may depend on the underlying lattice and weights (and, in the case of $D_{p},$ on local geometric data); a *lattice-independent*, or *universal* quantity, is allowed to depend only on $\Omega ,$ the boundary conditions, and the connection $\varphi .$ The parallel transport of $\varphi$ along the edges gives a discrete connection, and we can consider the corresponding discrete Laplacian $\Delta ^{\Omega ^{\delta },\varphi }$, with Dirichlet/Neumann boundary conditions approximating those in $\Omega$. We will denote the rank of $\varphi$ by *d*. The subject of the present paper is the asymptotics of $\mathrm {det}^{\star }\Delta ^{\Omega ^{\delta },\varphi }$ as $\delta \rightarrow 0$, where $\mathrm {det}^{\star }$ stands for the product of all non-zero eigenvalues.

We make the following conventions regarding the parameters, see Sect. 2 for details. The mesh size $\delta$ is defined so that the lattice has $\delta ^{-2}$ vertices per unit area. Thus, for a given lattice, $\delta =\frac{\delta _{0}}{N}$ for a constant $\delta _{0}$ and an integer *N*. We denote by $|\Omega ^{\delta }|$ the size of $\Omega ^{\delta },$ measured in the number of fundamental “plaquettes” (triangles or squares) in their discretization; thus as $\delta \rightarrow 0,$ $|\Omega ^{\delta }|$ is of order $\delta ^{-2}.$ Similarly, $|\partial _{\mathcal {D}}\Omega ^{\delta }|,|\partial _{\mathcal {N}}\Omega ^{\delta }|$ denote the size of Dirichlet and Neumann parts of the boundary, measured in the number of plaquette sides, thus $|\partial _{\mathcal {D}}\Omega ^{\delta }|,|\partial _{\mathcal {N}}\Omega ^{\delta }|$ have order $\delta ^{-1}$. We will also assume that the weights on the graph are normalized so that the random walk on $\Omega ^{\delta }$ converges to the standard Brownian motion.

Our main result is as follows:

### Theorem 1.1

As $\delta \rightarrow 0$, one has the following asymptotics:

1.1 $$\begin{aligned} \log \mathrm {det}^{\star }\Delta ^{\Omega ^{\delta },\varphi }=A\cdot |\Omega ^{\delta }|+B_{\mathcal {D}}\cdot |\partial _{\mathcal {D}}\Omega ^{\delta }|+B_{\mathcal {N}}\cdot |\partial _{\mathcal {N}}\Omega ^{\delta }|+C\cdot \log \delta +D+o(1),\nonumber \\ \end{aligned}$$

where:

- *A* and $B_{\mathcal {D}}=-B_{\mathcal {N}}$ are lattice-dependent constants that are expressed in terms of continuous time lattice heat kernels, see (4.3), (6.8).
- *C* is a lattice-independent constant given by
  $$\begin{aligned} C=-2\dim \ker \Delta ^{\Omega ,\varphi }-d\cdot \sum _{p\in \mathcal {C}^{}\cup \Upsilon \cup \mathcal {P}}C_{p}, \end{aligned}$$
  where $\Delta ^{\Omega ,\varphi }$ stands for the Friedrichs extension the Laplacian on $\Omega$ acting on sections of $\varphi$, see Sect. 3; the sum is over the set of conical singularities, corners, and punctures of $\Omega$, and the values $C_{p}$ for a cone $\mathcal {C}^{\alpha }$ of angle $\alpha$, corners $\Upsilon _{\mathcal {D}}^{\alpha },\Upsilon _{\mathcal {N}}^{\alpha },\Upsilon _{\mathcal {N}\mathcal {D}}$ of angle $\alpha$ with Dirichlet, Neumann, changing Neumann-to-Dirichlet boundary conditions, and a puncture $\mathcal {P}^{M}$ with monodromy operator *M* are given by
  $$\begin{aligned} C_{\mathcal {C}^{^{\alpha }}}\quad&=\frac{\alpha }{12\pi }-\frac{\pi }{3\alpha };&C_{\Upsilon _{\mathcal {D}}^{\alpha }}&=C_{\Upsilon _{\mathcal {N}}^{\alpha }}=\frac{\alpha }{12\pi }-\frac{\pi }{12\alpha };\\ C_{\Upsilon _{\mathcal {N}\mathcal {D}}^{\alpha }}\quad&=\frac{\alpha }{12\pi }+\frac{\pi }{24\alpha };&C_{\mathcal {P}^{M}}&=\pi ^{-2}\sum _{k=1}^{\infty }(1-d^{-1}\Re \mathfrak {e}\,\mathrm {Tr\,}M^{k})k^{-2} \end{aligned}$$
- the constant *D* has the form
  $$\begin{aligned} D=d\cdot \sum _{p\in \mathcal {C}^{}\cup \Upsilon \cup \mathcal {P}}D_{p}+\log \mathrm {det}^{\star }_{\zeta }\Delta ^{\Omega ,\varphi }, \end{aligned}$$
  where $D_{p}$ are lattice-dependent constants (entirely determined by the lattice and angle, boundary conditions, or the monodromy at a puncture *p*); and $\mathrm {det}^{\star }_{\zeta }\Delta ^{\Omega ,\varphi }$ is the zeta-regularized determinant of $\Delta ^{\Omega ,\varphi }$, see Sect. 3.

The constants $A,B_{\mathcal {D}},B_{\mathcal {N}}$ depend linearly on the rank *d* of the bundle, i.e., when divided by *d*, they only depend on the underlying lattice and the weights.

There is a lot of previous work on the subject. Discretizations of Laplacian were studied by Dodziuk [10] on a class of discretizations of Riemannian manifolds in arbitrary dimension. He established [10, Section 5] the convergence of the eigenvalues and the spectral zeta function of a combinatorial Laplacian with weights inherited from the Riemannian metric on the underlying manifold. Cardy and Peschel [5] conjectured that the asymptotic of the partition function of any critical 2D lattice model on a Riemann surface should take a form similar to (1.1). Since the determinants of the discrete Laplacian and its vector bundle versions are partition functions of a number of lattice models, such as dimers and double dimers, discrete GFF, spanning trees and cycle-rooted spanning forests [3, 11, 24, 25, 27, 28, 30, 31, 37], our results can be viewed as a rigorous proof of a particular case of the Cardy–Peschel conjecture. Duplantier and David [13] computed the asymptotics of the determinant of the discrete square lattice Laplacian on a torus and a rectangle; their results were extended to cylinder, Möbius strip and Klein bottle by Brankov–Priezzhev and Izmailyan–Oganesyan–Hu [4, 23]. The approach in these papers is based on the fact that these geometries have large groups of symmetries acting on them, and hence the discrete Laplacian can be diagonalized explicitly. The determinant is then an explicit product, whose asymptotics is still non-trivial, but doable e.g. by Euler–Maclaurin formula.

Kenyon [28] proved the asymptotic expansion of the type (1.1) in the case of the square lattice and planar simply connected rectilinear domains, with a slightly weaker control of the corner contributions. In this general setting, the explicit diagonalization of the discrete Laplacian is not available; instead, Kenyon’s method is based on tracking the variation of the determinant of the Laplacian when cutting the domain along vertical or horizontal line, using a relation to the dimer model. Ananth Sridhar [44] extended this result to the case of the Laplacian with smoothly changing inhomogeneous weights, by variation of these weights starting from Kenyon’s result.

Recently, Finski [16–18] obtained a version of Theorem 1.1 in the case of the square lattice quadrangulations of Riemann surfaces with Neumann boundary conditions and cone angles restricted to integer multiples of $\pi$, and with a slightly weaker control of the corner and cone contributions. In particular, that work extended the aforementioned result of Kenyon to multiply connected domains, and directly connected the constant term *D* to the $\zeta$-regularized determinant of the Friedrichs extension of the Laplacian, settling two of the open problems stated in [28, Section 8]. Like ours, Finski’s method uses the discrete spectral $\zeta$-function, but otherwise it is rather different. He starts by proving, by Rayleigh method, the convergence of individual eigenvalues and eigenfunctions of the discrete Laplacian [17]. From this, together with some estimates such as uniform Weyl’s law, it follows that the discrete spectral $\zeta$-function converges to its continuous counterpart in the region where its defining series converges absolutely. In order to deduce the asymptotics in a neighborhood of the origin, Finski follows Müller’s proof of the Ray–Singer conjecture, and regularizes the $\zeta$-function by comparing it to the trace of a sum of “localizations” of the powers of the Laplacian subordinate to a partition of unity. These localizations live on squares and on a number of model surfaces constructed using the infinite cones and infinite angles, and it turns out to be possible, with some work, to compute their asymptotics.

The zeta-regularized determinant of the Laplacian $\mathrm {det}^{\star }_{\zeta }\Delta ^{\Omega ,\varphi }$ that appears in the constant term of the expansion (1.1) goes back to Kronecker [32] who computed it for the torus. It has subsequently received a lot of attention with the introduction of analytic torsion by Ray and Singer [41, 42] and the celebrated proofs by Cheeger and Müller of its equivalence to the R-torsion. On the physics side, its importance stems from its role as a partition function of conformal field theories, and in particular, from its conformal transformation properties given by the Polyakov–Alvarez formula [2, 39, 40], see also [1] and the references therein for the most recent developments. For other related recent work, see [12, 22, 43, 44, 46].

The result of Theorem 1.1 improves on the state of the art in the following ways. First, our class of surfaces is more general, in that it allows for conical (and corner) angles any multiples of $\frac{\pi }{3}$ or $\frac{\pi }{2},$ and also for punctures. Second, we work simultaneously with *general geometries* that do not admit an explicit diagonalization of the Laplacian and in a *universal* setting, allowing for discretizations by arbitrary doubly periodic lattices, possibly with weights, with enough symmetries. Third, we allow for mixed Dirichlet and Neumann boundary conditions. Finally, we improve the estimates of the corner and cone contribution from $C_{p}\log \delta +o(\log \delta )$ to $C_{p}\log \delta +D_{p}+o(1).$

We also propose a new proof. The method is similar to that used by Chinta–Jorgenson–Karlsson [7, 8] and Friedli [19] who studied the square lattice Laplacians on a torus: we use a representation for $\log \mathrm {det}^{\star }\Delta ^{\Omega ^{\delta },\varphi }$ as an integral transform of the theta function, i.e., the trace of the discrete heat kernel. We then break the integral into parts that we analyze separately, see the key formula (4.1). The main idea is to regularize the discrete heat kernel by subtracting the heat kernel on one of a discretized model surface—the full plane, the half-plane, a punctured plane, a cone, or a corner—that matches the geometry of $\Omega ^{\delta }$ locally. This immediately isolates the volume term $A\cdot |\Omega ^{\delta }|$ in (1.1), and also the boundary, cone and puncture contributions, whose asymptotics can be analyzed separately by studying heat kernels on model surfaces. On the other hand, the regularized trace of the heat kernel receives only Brownian scale contributions, and after rescaling converges to its continuous counterpart, more or less, by the local Central limit theorem. We derive this convergence from the functional CLT in the plane and the parabolic Harnack inequality of Delmotte. To complete the asymptotic analysis, we need to pass to the limit under the integral; to this end, we employ a large deviation estimate for small *t* and a uniform spectral gap bound for large *t*.

Thus, essentially, we only use three ingredients: the functional Central limit theorem, parabolic regularity, and the fact that microscopically, our lattice approximation “looks the same at all places”. The only reason we do not consider more general surfaces (e.g., allowing for conical singularities with arbitrary angles, or for genuinely curved surfaces) is that those do not admit nice discretizations with this last property; see Remark 9.2. To see the difficulty it entails, note that the constant *A*, being essentially the free energy per lattice site in the infinite volume limit, is lattice-dependent; if the lattice is different in different places of $\Omega ^{\delta }$, then *A* will also fluctuate, and the volume term in the asymptotics may be hard to control with meaningful precision. This is why we believe that approximation schemes such as Dodziuk’s are too general to admit asymptotic formulae like (1.1). On the positive side, apart from the volume term (4.3), the rest of our analysis, at least in the absence of the boundary, does not use regularity of the lattice in any essential way. This leaves hope that the method can be applied to more general surfaces and approximations in sufficiently integrable case, e.g. on isoradial graphs.

Note that if we only want to study the the difference $\log \mathrm {det}^{\star }\Delta ^{\Omega ^{\delta },\varphi _{1}}-\log \mathrm {det}^{\star }\Delta ^{\Omega ^{\delta },\varphi _{2}}$, the aforementioned volume term cancels out, as do most of other terms in the asymptotics, and and our method yields a simple proof the asymptotics $2(\dim \ker \varphi _{1}-\dim \ker \varphi _{2})\log \delta +\log \mathrm {det}^{\star }_{\zeta }\Delta ^{\Omega ,\varphi _{1}}-\log \mathrm {det}^{\star }_{\zeta }\Delta ^{\Omega ,\varphi _{2}}$, using only the functional CLT and the parabolic regularity. Such differences are of interest since they often compute interesting topological observables in the models, see e.g. [11, 12, 24–26], and there are a number of results in this direction. Dubédat and Gheissari [12] proved convergence for tori in a different way under even weaker assumptions and Kassel–Kenyon [25] proved a similar result on general Riemann surfaces, without identifying the limit. A remark on cancellation of the singular terms in this setting is also made by Kenyon [28], for trivial line bundles with different simply-connected domains, and Finski [18], for general bundles.

Some of the ingredients of our proof could be alternatively established by the methods [17, 18]; e.g., the convergence of theta function in the Brownian scale follows easily from the convergence of rescaled eigenvalues and the uniform Weyl law, as does the spectral gap estimate of Lemma 5.5. Arguably, our proofs via the functional CLT are more streamlined; for example, cones and conical singularities, that seem to be a source of technical difficulties in [17], do not enter this part of our proof at all, since the Brownian motion avoids them almost surely. On the other hand, some intermediate results and techniques in [17, 18] are of independent interest.

Our method gives the explicit values of $A,B_{\mathcal {D}}=-B_{\mathcal {N}}$ and $D_{p}$ as sums of integrals of discrete (continuous time) heat kernels on the discretizations of the plane, half-plane, corners, or cones. Note, however, that to determine those constant, it suffices to compute the asymptotics (1.1) in some simple geometry; for example, a torus for *A*, or a cylinder or a square (with corresponding boundary conditions) for $B_{\mathcal {D}},B_{\mathcal {N}}.$ Thus, on the square lattice, it follows from [13, Eq. (4.24) and (5.14)] that

$$\begin{aligned} A^{\square }=\frac{4G}{\pi }-\log 2,\quad B_{\mathcal {D}}^{\square }=\frac{1}{2}\log (\sqrt{2}-1),\quad B_{\mathcal {N}}^{+}=\frac{1}{2}\log (\sqrt{2}-1)-\frac{1}{2}\log 2. \end{aligned}$$

where *G* is the Catalan’s constant $1-\frac{1}{3^{2}}+\frac{1}{5^{2}}-\dots$ Note that [13] computes $B_{\mathcal {D}}^{\square }$ and $B_{\mathcal {N}}^{+}$ on two *different* discretizations of a square (see Fig. 4), shifted with respect to each other by $\frac{\delta }{2}+i\frac{\delta }{2}$, and $B_{\mathcal {D}}^{\square }$ is related to $B_{\mathcal {N}}^{+}$ by the Uniform Spanning tree duality. By contrast, our identity $B_{\mathcal {D}}=-B_{\mathcal {N}}$ holds for a *fixed* discretization. An expression for *A* in terms of polylogarithms is known, by a different method, for isoradial graphs with critical weights, including the hexagonal and the triangular lattices [29, Theorem 1.1]. In Sect. 8, we compute closed-form expressions for $B_{\mathcal {D}}=-B_{\mathcal {N}}$ for triangular lattice (rotated in two different ways), for the square lattice rotated by $45^{\circ }$, and give an alternative computation in the case of a non-rotated square lattice, recovering the result of [13]. Note that the specific values of the constants are sensitive to conventions such as the definition of $|\Omega ^{\delta }|$ and the normalization of weights, hence e.g. the extra $-\log 2$ in $A^{\square }$ above compared to [13, 18, 28].

In our proof, we employ the functional Central limit theorem with an error bound that we derive from Einmahl’s multidimensional KMT coupling [14]. The only place the error bound is used in earnest is when we sharpen the asymptotics of the contribution of cones and corners to (1.1), from $C_{p}\log \delta +o(\log \delta )$ in [18, 28] to $C_{p}\log \delta +D_{p}+o(1).$ (We also use the error bound it in the computation of the boundary contribution; however, there we do not need the *functional* CLT, so e.g. Berry–Esseen bounds would suffice.) Since any power law error bound suffices for that, one could use e.g. the Skorokhod embedding instead, cf. [34, Theorem 3.4.2]; see also [34, Section 7.2] for simple proofs of the KMT coupling on the square and the triangular lattices. The same error bound allows one to track the rate of convergence in Lemma 5.6, Corollary 6.4, and (6.2), (6.6), improving the *o*(1) in (1.1) to $O(\delta ^{\rho })$ with $\rho >0$. The recent independent work [21] gives explicitly the error bound on the square lattice; it also shows that similar methods allow one to treat polygonal boundaries with any rational slopes.

The assumption that the weights are symmetric is only used in the proofs of technical Lemmas 5.1–5.6, where we found it convenient to use the parabolic Harnack inequality of Delmotte [9]; we note that we use the continuous time version that is significantly simpler than the discrete time one. With some work, these lemmas, modified accordingly, can be given alternative proofs, allowing one to lift the symmetry assumption. The same applies to the unitarity of $\varphi$, which can probably be relaxed under the assumption that the real parts of the eigenvalues of the Laplacian remain positive.

## Laplacians, Heat Kernels and Zeta Functions

Although we are mainly interested in the discretizations of the Riemann surfaces, we start from a more general setup. Let $G=(\mathcal {V},\mathcal {E})$ be a connected finite undirected graph with weights $w_{e}>0$ assigned to its edges. A *vector bundle* of rank *d* over *G* is a collection of *d*-dimensional complex vector spaces $V_{x}$ attached to its vertices $x\in \mathcal {V}$. A *connection* on a vector bundle is a collection of linear isomorphisms $\varphi _{xy}=\varphi _{yx}^{-1}:V_{x}\rightarrow V_{y}$ for each pair $x\sim y$ of adjacent vertices. From now on, we will only consider *unitary* connections, that is, each $V_{x}$ is equipped with an inner product and the maps $\varphi _{xy}$ are unitary. A *section* *f* of a vector bundle is a choice of an element $f(x)\in V_{x}$ for each $x\in \mathcal {V}$; we will take the liberty to refer to it as a section of $\varphi$ in order to lighten the notation. When the vector bundle is unitary, the linear space of it sections comes with the inner product $\langle f;g\rangle =\sum _{x\in \mathcal {V}}\langle f(x);g(x)\rangle$.

The Laplace operator $\Delta ^{G,\varphi }$ acts on sections of the vector bundles by the formula

$$\begin{aligned} \left( \Delta ^{G,\varphi }f\right) (x):=\sum _{(yx)\in \mathcal {E}}w_{xy}\left( f(x)-\varphi _{yx}f(y)\right) . \end{aligned}$$

Note that with respect to the inner product as above, we have

$$\begin{aligned} \langle \Delta ^{G,\varphi }f;g\rangle =\frac{1}{2}\sum _{(xy)\in \mathcal {E}}w_{xy}\langle \varphi _{yx}f(y)-f(x);\varphi _{yx}g(y)-g(x)\rangle \end{aligned}$$

which shows that $\Delta ^{G,\varphi }$ is non-negative and self-adjoint and therefore diagonalizable with real non-negative eigenvalues.

We define the *heat operator* associated to $\Delta ^{G,\varphi }$ by

$$\begin{aligned} P_{t}^{G,\varphi }:=\exp (-t\Delta ^{G,\varphi }). \end{aligned}$$

This is again a linear operator acting on the linear space of sections of the vector bundle. By linearity, we can write $(P_{t}^{G,\varphi }f)(y)=\sum _{x\in \mathcal {V}}P^{G,\varphi }(x,y,t)f(x)$, where $P^{G,\varphi }(x,y,t):V_{x}\rightarrow V_{y}$ is a linear operator, called the *heat kernel*.

The trace of $P_{t}^{G,\varphi }$ is called the *theta function*. By computing the trace first as the sum of eigenvalues, and then as the sum of the diagonal elements, we get the *theta inversion identity*

2.1 $$\begin{aligned} \Theta ^{G,\varphi }(t):=\mathrm {Tr\,}P_{t}^{G,\varphi }=\sum _{\lambda \in \sigma (\Delta ^{G,\varphi })}e^{-\lambda t}=\sum _{x\in \mathcal {V}}\mathrm {Tr\,}P^{G,\varphi }(x,x,t). \end{aligned}$$

The *zeta function* associated to $\Delta ^{G,\varphi }$ is the Mellin transofrm of $\Theta ^{G,\varphi }(t)$, defined for $s\in \mathbb {C}$ as

$$\begin{aligned} \zeta ^{G,\varphi }(s):=\sum _{0\ne \lambda \in \sigma (\Delta ^{G,\varphi })}\lambda ^{-s}. \end{aligned}$$

Let $k=\dim \ker \Delta ^{G,\varphi }$. By subtracting *k* from both sides of the theta inversion formula, multiplying by $t^{s-1}$ and integrating, one gets

2.2 $$\begin{aligned} \zeta ^{G,\varphi }(s)=\frac{1}{\Gamma (s)}\int _{0}^{\infty }(\Theta ^{G,\varphi }(t)-k)t^{s-1}\,dt,\quad \text {if }\Re \mathfrak {e}\,(s)>0; \end{aligned}$$

note that the integral converges at infinity because of the positivity of the eigenvalues. Our main motivation for studying the zeta function is the identity

2.3 $$\begin{aligned} -\left( \zeta ^{G,\varphi }\right) '(0)=\sum _{0\ne \lambda \in \sigma (\Delta ^{G,\varphi })}\log \lambda =\log \mathrm {det}^{\star }\left( \Delta ^{G,\varphi }\right) . \end{aligned}$$

We will need a probabilistic interpretation of the heat kernel. Let $\gamma _{t}$ denote the continuous time random walk on the weighted graph (*G*; *w*), that, being at $x\in \mathcal {V}$, moves following exponential clock to an adjacent vertex *y* with intensity $w_{xy}$. We denote $\varphi _{\gamma _{[0;t]}}:=\varphi _{x_{n-1}x_{n}}\circ \dots \circ \varphi _{x_{0}x_{1}}$, where $\gamma _{0}=x_{0}\sim x_{1}\sim \dots \sim x_{n}=\gamma _{t}$ are the vertices visited consecutively by $\gamma$ up to time *t*. We denote by $\mathbb {P}^{x}$ and $\mathsf {\mathbb {E}}^{x}$ the probability and the expectation with respect to this random walk started at *x*. We have the following lemma:

### Lemma 2.1

We have

2.4 $$\begin{aligned} P^{G,\varphi }(x,y,t)=\mathsf {\mathbb {E}}^{x}(\varphi _{\gamma _{[0,t]}}\mathbb {I}_{\gamma _{t}=y}). \end{aligned}$$

### Proof

Denote the right-hand side by ${\hat{P}}^{G,\varphi }(x,y,t)$. We observe that

$$\begin{aligned} \partial _{t}{\hat{P}}^{G,\varphi }(x,y,t)+\Delta _{x}^{G,\varphi }{\hat{P}}^{G,\varphi }(x,y,t)=0, \end{aligned}$$

and the same system of ODEs with the same initial conditions holds for $P^{G,\varphi }(x,y,t)$, and the solution is unique. $\square$

We use (2.4) to extend the definition of the heat kernel to infinite graphs.

In what follows, the graph *G* will be a discretization of a triangulated or a quadrangulated surface $\Omega$, as in the introduction. To discretize $\Omega$, choose an infinite locally finite weighted connected graph $\mathbb {C}^{\delta _{0}}$ with vertices embedded in the plane; we assume that the embedded weighted graph is bi-periodic with periods either 1 and $\frac{1}{2}+\frac{\sqrt{3}}{2}i$ (for triangulation case), or 1 and *i* (for quadrangulation case) and has $\delta _{0}^{-2}$ vertices per unit area. We moreover assume that $\mathbb {C}^{\delta _{0}}$ is preserved under rotations by $\pi /3$, respectively, $\pi /2$, around the origin, and under reflections with respect to the real line. We denote $\mathbb {C}^{\delta }=\frac{1}{N}\mathbb {C}^{\delta _{0}},$ where $\delta =\delta _{0}/N$, $N\in \mathbb {N}$, so that $\mathbb {C}^{\delta }$ has $\delta ^{-2}$ vertices per unit area. Let *T* denote the unit triangle $\{0;1;\frac{1}{2}+\frac{\sqrt{3}}{2}i\}$ (respectively, the unit square $\{0,1,1+i,i\}$). We denote by $\Omega ^{\delta }$ the discrete surface obtained by discretizing each triangle/square in $\Omega$ with $T^{\delta }=\mathbb {C}^{\delta }\cap T$; since $\mathbb {C}^{\delta }$ has all the symmetries of $\mathbb {C}^{\delta _{0}}$, these discretizations can be naturally glued together. We will interchangeably use $\delta$ and $N=\delta _{0}\delta ^{-1}$ as mesh parameters of the discretization. We do not require $\mathbb {C}^{\delta _{0}}$ to be properly embedded, i.e., edges are allowed to intersect; however, we do assume that the graph obtained by removing edges connecting a vertex strictly inside *T* with one strictly outside *T* is still connected, so that $\Omega ^{\delta }$ is connected.

As an example, $\mathbb {Z}+i{{\mathbb {Z}}}$ and $\frac{1}{2}+\frac{i}{2}+\mathbb {Z}+i\mathbb {Z}$ can serve as $\mathbb {C}^{\delta _{0}}$ (with $\delta _{0}=1$ in this case) in the quadrangulated case, leading to two different families of discretizations (one of them will have vertices at cone tips). The square lattice rotated by $45^{\circ }$ yields another discretization. The triangular and the hexagonal lattices can serve as $\mathbb {C}^{\delta _{0}}$ in the triangulation case, with $\delta _{0}=\frac{\root 4 \of {3}}{\sqrt{2}}$ and $\delta _{0}=\frac{\root 4 \of {3}}{2}$ respectively, and also one may choose to rotate them, see Fig. 4.

It is easy to show (see Lemma 10.2 below) that the continuous time random walk on $\mathbb {C}^{\delta }$ satisfies the Central limit theorem, i.e., converges, as $\delta \rightarrow 0,$ to a Brownian motion, whose covariance matrix must be scalar, because of the symmetries. We will assume that the weights $w_{xy}$ are chosen so that this matrix is the identity, i.e., $\sum _{y\in B}P^{\mathbb {C}^{\delta }}(x,y,\delta ^{-2}t){\mathop {\longrightarrow }\limits ^{\delta \rightarrow 0}}\int _{B}\frac{1}{2\pi t}\exp (-\frac{|x-y|^{2}}{2t})dy$ for any disc *B*. This can always be achieved by simultaneously multiplying all the weights by a common factor, which only affects the values of the lattice-dependent constants in (1.1).

Let us comment on the boundary conditions. We will assume that if a point $p\in \partial \Omega$ of Dirichlet-to-Neumann change is at a corner, then, at $\Omega ^{\delta },$ the boundary conditions also change at the corresponding corner, and if *p* is an inner point of a side of one of the triangles/squares, then it is approximated by sequence of points $p^{\delta }\rightarrow p$ at distance $m^{\delta }/N$, $m^{\delta }\in \mathbb {N}$, from the corner of that triangle (or square). To define an action of $\Delta ^{\Omega ^{\delta },\varphi }$ on a section *f* with Dirichlet (respectively, Neumann) boundary condition at a boundary segment *l*, we extend *f* across *l* by $f(z^{\star })={\mp } f(z),$ where $z\mapsto z^{\star }$ is the reflection with respect to *l*; if there are vertices on the Dirichlet boundary, we only consider sections *f* that are zero at those vertices. This procedure may lead to non-symmetric weights that are gauge equivalent to symmetric ones; the above formulae are not affected. We adopt the convention that when writing the sum over $x\in \Omega ^{\delta }$ (as e.g. in (2.1)) we do not include vertices lying on the Dirichlet part of the boundary, but do include those on the Neumann part.

An approximation $p^{\delta }$ to a puncture *p* will be realized as a point of $\Omega$ disjoint from any edge of $\Omega ^{\delta }$; we moreover insist that it is an image under scaling of a *fixed* point in a fundamental domain of $\mathbb {C}^{\delta _{0}}.$

## Heat Kernels and Theta Functions in the Continuum

We recall a continuous version of the above theory, in which the random walk $\gamma ^{\delta }$ on $\Omega ^{\delta }$ is replaced by the Brownian motion $\gamma _{t}$ on $\Omega ,$ reflected at $\partial _{\mathcal {N}}\Omega$ and absorbed at $\partial _{\mathcal {D}}\Omega$. This is only needed for the interpretation of the constant term in the asymptotics (1.1); a reader willing to accept (3.3–3.5) as the definition of the zeta-reguralized determinant of the Laplacian may skip most of the rest. Recall [38] that the Laplacian on surfaces with conical singularities is not essentially self-adjoint; hence some care is needed when specifying its self-adjoint extension, see also [17, Section 2.3].

We start by constructing the Brownian motion on $\Omega ,$ which can be done by elementary means. Let ${\hat{\Omega }}$ be two copies of $\Omega$ glued along the boundary with conical singularities removed; we can define the Brownian motion ${\hat{\gamma }}$ on ${\hat{\Omega }}$ by coupling it to the Brownian motion ${\check{\gamma }}$ in the plane, lifting ${\check{\gamma }}$ to ${\hat{\Omega }}$ by local isometries. The lifting is well defined at least up to the first time ${\hat{\gamma }}$ hits a conical singularity of ${\hat{\Omega }}$, but this can only happen when ${\check{\gamma }}$ hits a point of a triangular or a square lattice, i.e., with probability 0. Hence, almost surely for $\mathrm {Leb}({\hat{\Omega }})$ a.e. starting point, ${\hat{\gamma }}_{t}$ is defined for all *t*. We then define $\gamma _{t}$ to be ${\hat{\gamma }}_{t}$ reflected to $\Omega$ and stopped upon hitting $\partial _{\mathcal {D}}\Omega .$

### Lemma 3.1

The Markov process $\gamma _{t}$ is symmetric with respect to the Lebesgue measure on $\Omega .$ Moreover, for each fixed $t>0,$ its transition kernel $P^{\Omega }(\cdot ,\cdot ,t)$ is bounded, and it is smooth away from $\partial \Omega$ and the cone tips.

### Proof

See Sect. 10. $$\square $$

The following construction is general for symmetric Markov processes, see [45, Section 1.4] or [20, Section 1.4]. Let $L^{2,\varphi }(\Omega )$ be the set of $L^{2}$ sections of $\varphi .$ Define the *Markov semi-group* $P_{t}^{\Omega ,\varphi }:L^{2,\varphi }(\Omega )\rightarrow L^{2,\varphi }(\Omega )$ by

3.1 $$\begin{aligned} (P_{t}^{\Omega ,\varphi }f)(x):=\,&\mathsf {\mathbb {E}}^{x}\left[ \varphi _{\gamma _{[0,t]}}^{-1}(f(\gamma _{t}))\right] \nonumber \\ =&\int _{y\in \Omega }\mathsf {\mathbb {E}}^{x}\left[ \varphi _{\gamma _{[0,t]}}^{-1}(f(y))|\gamma _{t}=y\right] P^{\Omega }(x,y,t)\,dy\nonumber \\ =&\int _{y\in \Omega }P^{\Omega ,\varphi }(x,y,t)f(y)dy. \end{aligned}$$

From the symmetry of $\gamma _{t}$ and the unitarity of $\varphi ,$ we see that $P_{t}^{\Omega ,\varphi }$ are self-adjoint; also, $\left\| P^{\Omega ,\varphi }\right\| \le P^{\Omega }$ pointwise. Hence, by Cauchy–Schwarz,

$$\begin{aligned} \left\| (P_{t}^{\Omega ,\varphi }f)(x)\right\| ^{2}\le \int \left\| P^{\Omega ,\varphi }(x,y,t)\right\| \,dx\int _{y\in \Omega }\left\| P^{\Omega ,\varphi }(x,y,t)\right\| \left\| f(y)\right\| ^{2}\,dy\le \left\| f\right\| ^{2}, \end{aligned}$$

i.e., for each $t>0,$ $P_{t}^{\Omega ,\varphi }$ is a self-adjoint contraction on $L^{2,\varphi }(\Omega )$. The semi-group $P_{t}^{\Omega ,\varphi }$ is strongly continuous: it is enough to check that $P_{t}^{\Omega ,\varphi }f{\mathop {\longrightarrow }\limits ^{t\rightarrow 0}}f$ in $L^{2}$ for a continuous *f*, in which case the convergence clearly holds uniformly. Put $D_{t}^{\Omega ,\varphi }=t^{-1}(I-P_{t}^{\Omega ,\varphi });$ by looking at the spectral decomposition of the semigroup $\{P_{t}^{\Omega ,\varphi }\}_{t>0}$, we see that $\langle D_{t}^{\Omega ,\varphi }f,f\rangle$ is decreasing in *t*. We define the *Dirichlet form *$\mathcal {E}^{\Omega ,\varphi }(f,f):=\lim _{t\searrow 0}\langle D_{t}^{\Omega ,\varphi }f,f\rangle$ on the set $\mathcal {D}(\mathcal {E}^{\Omega ,\varphi })$ of all $f\in L^{2,\varphi }(\Omega )$ for which the limit is finite. The form $\mathcal {E}^{\Omega ,\varphi }$ is non-negative and closed [45, Lemma 4.2], and we denote by $\Delta ^{\Omega ,\varphi }$ the unique non-negative self-adjoint operator associated to $\mathcal {E}^{\Omega ,\varphi },$ called the *generator* of $\{P_{t}^{\Omega ,\varphi }\}_{t>0}.$ In terms of the common spectral projections $E_{\lambda }$ for $P_{t}^{\Omega ,\varphi },$ we have $P_{t}^{\Omega ,\varphi }f=\int _{0}^{\infty }e^{-\lambda t}\,dE_{\lambda }f,$ $\Delta ^{\Omega ,\varphi }f=\int _{0}^{\infty }\lambda \,dE_{\lambda }f,$ and $\mathcal {E}^{\Omega ,\varphi }(f,g)=\int _{0}^{\infty }\lambda \langle dE_{\lambda }f,g\rangle ,$ with the domains $L^{2,\varphi }(\Omega ),$ $\mathcal {D}(\Delta ^{\Omega ,\varphi })=\{f\in L^{2,\varphi }(\Omega ):\int _{0}^{\infty }\lambda ^{2}\,\langle dE_{\lambda }f,f\rangle <\infty \}$ and $\mathcal {D}(\mathcal {E}^{\Omega ,\varphi })=\{f\in L^{2,\varphi }(\Omega ):\int _{0}^{\infty }\lambda \,\langle dE_{\lambda }f,f\rangle <\infty \}$ respectively.

Since $P^{\Omega ,\varphi }(\cdot ,\cdot ,t)$ is bounded, $P_{t}^{\Omega ,\varphi }$ is Hilbert–Schmidt, and hence $P_{2t}^{\Omega ,\varphi }$ is trace class for all $t>0.$ Therefore, we can define the* spectral theta function* by

3.2 $$\begin{aligned} \Theta ^{\Omega ,\varphi }(t)=\mathrm {Tr\,}P_{t}^{\Omega ,\varphi }=\sum _{i=1}^{\infty }e^{-\lambda _{i}t},\quad t>0. \end{aligned}$$

where $0\le \lambda _{1}\le \lambda _{2}\le \dots$ are the eigenvalues of $\Delta ^{\Omega ,\varphi }.$ We have, for any $f\in L^{2}(\Omega ),$

$$\begin{aligned} \int _{\Omega }P^{\Omega ,\varphi }(x,y,t)f(y)dy\!=\!(P_{t}^{\Omega ,\varphi }f)(x)\!=\!\sum _{i=1}^{\infty }(P_{t}\psi _{i})(x)\langle \psi _{i};f\rangle \!=\!\sum _{i}e^{-\lambda _{i}t}\psi _{i}(x)\langle \psi _{i};f\rangle , \end{aligned}$$

thus, for any vector $v\in V_{y}$, $P^{\Omega ,\varphi }(x,y,t)v=\sum _{i}e^{-\lambda _{i}t}\psi _{i}(x)\langle \psi _{i}(y),v\rangle ,$ so that $\mathrm {Tr\,}P^{\Omega ,\varphi }(x,x,t)=\sum _{i}e^{-\lambda _{i}t}\langle \psi _{i}(x);\psi _{i}(x)\rangle$ and, integrating, we arrive at the continuous theta inversion identity,

3.3 $$\begin{aligned} \int _{x\in \Omega }\mathrm {Tr\,}P^{\Omega ,\varphi }(x,x,t)\,dx=\Theta ^{\Omega ,\varphi }(t). \end{aligned}$$

We define the *spectral zeta function* by

3.4 $$\begin{aligned} \zeta ^{\Omega ,\varphi }(s)=\frac{1}{\Gamma (s)}\cdot \int _{0}^{\infty }(\Theta ^{\Omega ,\varphi }(t)-k)t^{s-1}\,dt,\quad \Re \mathfrak {e}\,s>1, \end{aligned}$$

postponing the proof of convergence to Sect. 9. Here $k=\dim \ker \Delta ^{\Omega ,\varphi };$ we can also describe *k* explicitly (for a connected $\Omega$) as follows: if $\partial _{\mathcal {D}}\Omega \ne \emptyset ,$ then $k=0,$ otherwise, *k* is the dimension of the *maximal trivial sub-bundle*. This description follows from Lemma 5.5, which uses the latter definition *k*, and whose conclusion, together with (5.4), implies that both $\Theta ^{\delta }(t)-k$ and $\Theta (t)-k$ tend to zero as $t\rightarrow \infty$. In particular, $k=\dim \ker \Delta ^{\Omega ,\varphi }=\dim \ker \Delta ^{\Omega ^{\delta },\varphi }=k^{\delta }$. Alternatively, one could note from Lemma A.4 that, if $\psi \in \ker \Delta ^{\Omega ,\varphi },$ then $\langle \nabla ^{\varphi }\psi ,\nabla ^{\varphi }\psi \rangle =0,$ that is, $\psi$ is a *covariant constant*, also known as *a flat section*, cf. [17, Corollary 2.6].

As in the discrete case, we have $\zeta ^{\Omega ,\varphi }(s)=\sum _{i}\lambda _{i}^{-s}$ whenever either the series or the integral (3.4) converges absolutely. Moreover, in in Sect. 9, we analytically continue $\zeta ^{\Omega ,\varphi }$ into a neighborhood of the origin, cf. [6]. In analogy with (2.3), this allows one to define the zeta-regularized determinant of $\Delta ^{\Omega ,\varphi }$ (cf. [41, 42]) by

3.5 $$\begin{aligned} \log \mathrm {det}^{\star }_{\zeta }\left( \Delta ^{\Omega ,\varphi }\right) =-\left( \zeta ^{\Omega ,\varphi }\right) '(0). \end{aligned}$$

It is possible to describe the form $\mathcal {E}^{\Omega ,\varphi }$ and its domain $\mathcal {D}(\mathcal {E}^{\Omega ,\varphi })$ (or, equivalently, the generator $\Delta ^{\Omega ,\varphi }$) more explicitly, which we postpone to the Appendix.

**Fig. 2:**
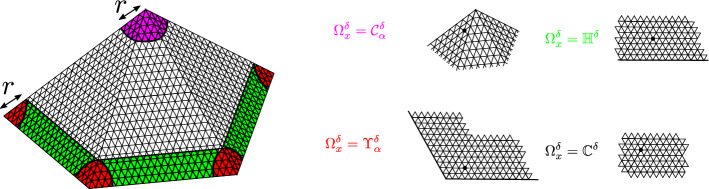
The discretized surface $\Omega ^{\delta }_{x}$ is a plane, half-plane, a infinite cone or an infinite wedge, depending on the local geometryof $\Omega ^{\delta }$ near *x*

## The Key Formula for the Determinant of the Discrete Laplacian

For notational simplicity, we first assume there are no punctures, and also that the lattice is such that there are no vertices at conical singularities. We will then discuss the necessary modifications in the general case.

**Fig. 3:**
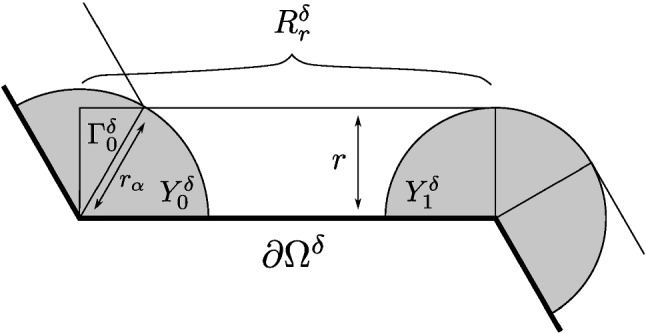
A decomposition of a neighborhood of a boundary segment. The rectangle $R_{r}^{\delta }$ includes two sectors $Y_{0,1}^{\delta }$ and a triangle $\Gamma _{0}^{\delta };$ in this case, $\Gamma _{1}^{\delta }=\emptyset$ since the corresponding angle is greater than $\pi$. The shaded sectors of radii $r_{\alpha }=r/\sin \frac{\alpha }{2}$ for $\alpha =2\pi /3$ and $r_{\alpha }=r$ for $\alpha =4\pi /3>\pi$ are the regions for which $\Omega ^{\delta }_{x}$ is a wedge; in the white part of $R_{r}^{\delta },$ $\Omega _{x}^{\delta }=\mathbb {H}^{\delta }$

Given $x\in \Omega$, we define $\Omega _{x}$ to be one of the model surfaces, namely, a plane, a half-plane, an infinite cone, or an infinite wedge, that agrees with $\Omega$ locally near *x*, see Fig. 2. More precisely, fix a small $r>0$ in such a way that the 2*r*-neighborhoods of the tips of the conical singularities and the boundary components do not overlap or self-overlap. We define $r_{\alpha }:=r/\sin (\alpha /2)$ if $\alpha <\pi$ and $r_{\alpha }=r$ otherwise. We then define $\Omega _{x}$ to be the wedge of angle $\alpha$ if *x* is at distance at most $r_{\alpha }$ from the tip of a corner (see Fig. 3) with angle $\alpha$, else, if *x* at distance $\le r$ from $\partial \Omega$ (or a conical singularity), we define $\Omega _{x}$ to be the half-plane (respectively, the cone); else, $\Omega _{x}$ is a plane.

In a similar way (with the same *r* independent of $\delta$), we define the infinite graphs $\Omega _{x}^{\delta }$ discretizing each respective $\Omega _{x}$. These graphs, when they have a boundary, come equipped with boundary conditions inherited from $\Omega ^{\delta }$. We define the heat kernel *P* in each of the discrete model domains $\Omega _{x}^{\delta }$ by (2.4), with $\varphi$ the trivial connection on the rank one bundle, and the random walk being stopped at the Dirichlet boundary and reflected at the Neumann one.

We start from (2.2) and rewrite it as

$$\begin{aligned} \zeta ^{\Omega ^{\delta },\varphi }(s)&=\frac{1}{\Gamma (s)}\int _{\delta ^{-2}}^{\infty }\left( \Theta ^{\Omega ^{\delta },\varphi }(t)-k\right) t^{s-1}\,dt\\&\quad +\frac{1}{\Gamma (s)}\sum _{x\in \Omega ^{\delta }}\int _{0}^{\delta ^{-2}}\left( \mathrm {Tr\,}P^{\Omega ^{\delta },\varphi }(x,x,t)-d\cdot P^{\Omega _{x}^{\delta }}(x,x,t)\right) t^{s-1}\,dt\\&\quad +\frac{d}{\Gamma (s)}\sum _{x\in \Omega ^{\delta }}\int _{0}^{\infty }\left( P^{\Omega _{x}^{\delta }}(x,x,t)-P^{\mathbb {C}^{\delta }}(x,x,t)\right) t^{s-1}\,dt\\&\quad -\frac{d}{\Gamma (s)} \sum _{x\in \Omega ^{\delta }}\int _{\delta ^{-2}}^{\infty }P^{\Omega _{x}^{\delta }}(x,x,t)t^{s-1}dt\\&\quad +\frac{d}{\Gamma (s)}\sum _{x\in \Omega ^{\delta }}\int _{0}^{\infty }\left( P^{\mathbb {C}^{\delta }}(x,x,t)-e^{-w_{x}t}\right) t^{s-1}\,dt\\&\quad +\frac{d}{\Gamma (s)}\sum _{x\in \Omega ^{\delta }}\int _{0}^{\infty }e^{-w_{x}t}t^{s-1}\,dt-\frac{1}{\Gamma (s)}\int _{0}^{\delta ^{-2}}kt^{s-1}\,dt, \end{aligned}$$

where $w_{x}:=\sum _{y\sim x}w_{xy}.$ This identity is valid for $0<\Re \mathfrak {e}\,s<1$, since each integral defines an analytic function in that region (see Lemma 6.2 below for large *t* bounds). Moreover, all the integrals but the last two are in fact analytic at least in $-1<\Re \mathfrak {e}\,s<1$; indeed it follows from (2.4) that $P^{\mathbb {C}^{\delta }}(x,x,t)=e^{-w_{x}t}\cdot \mathrm {Id}+O(t^{2}),$ where $e^{-w_{x}t}$ is the probability that the random walk does not move at all by time *t*,  and $O(t^{2})$ is the probability that it makes at least two steps, which is bounded from above by $\max _{y\in T}(1-e^{-w_{x}t})(1-e^{-w_{y}t})$. The last two terms are equal to

$$\begin{aligned} d\sum _{x\in \Omega ^{d}}w_{x}^{-s}-\frac{k\delta ^{-2s}}{s\Gamma (s)}, \end{aligned}$$

and since $1/\Gamma (s)=s(1+o(1))$ as $s\rightarrow 0$, the derivative of $\zeta ^{\Omega ^{\delta },\varphi }$ at zero evaluates to

4.1 $$\begin{aligned}&-\log \mathrm {det}^{\star }\Delta ^{\Omega ^{\delta },\varphi }\nonumber \\&\quad =\int _{\delta ^{-2}}^{\infty }\left( \Theta ^{\Omega ^{\delta },\varphi }(t)-k\right) \frac{dt}{t}+\int _{0}^{\delta ^{-2}}\sum _{x}\left( \mathrm {Tr\,}P^{\Omega ^{\delta },\varphi }(x,x,t)-d\cdot P^{\Omega _{x}^{\delta }}(x,x,t)\right) \frac{dt}{t}\nonumber \\&\qquad -\, d\cdot \int _{\delta ^{-2}}^{\infty }\sum _{x}P^{\Omega _{x}^{\delta }}(x,x,t)\frac{dt}{t}+d\cdot \int _{0}^{\infty }\sum _{x}\left( P^{\Omega _{x}^{\delta }}(x,x,t)-P^{\mathbb {C}^{\delta }}(x,x,t)\right) \frac{dt}{t}\nonumber \\&\qquad +\, d\sum _{x}\left( \int _{0}^{\infty }\left( P^{\mathbb {C}^{\delta }}(x,x,t)-e^{-w_{x}t}\right) \frac{dt}{t}-\log w_{x}\right) +2k\log \delta -k\gamma _{\mathrm {Euler}}. \end{aligned}$$

This is our **key formula**; analyzing it term by term will lead to (1.1). The last two terms are already explicit, and we see the $-2k=-2\dim \ker \Delta ^{\Omega ,\varphi }$ contribution to the logarithmic term in (1.1). For the first three integrals, going back to the probabilistic interpretation of the heat kernel, we observe that only the walks with $\gtrsim \delta ^{-1}$ steps contribute, hence, these terms converge to their continuous counterparts by Central limit theorem, see Sect. 5 and Corollary 6.4 for details. As for the fourth term, we note that the summands are zero unless *x* is *r*-close to a conical singularity or to the boundary. Thus, the whole sum only depends on the number of conical singularities and their angles, and on the geometry of the boundary. We treat it in Sect. 6.

Turning to the fifth term in (4.1), denote

4.2 $$\begin{aligned} A_{x}:=\int _{0}^{\infty }\left( P^{\mathbb {C}^{\delta }}(x,x,t)-e^{-w_{x}t}\right) \frac{dt}{t}-\log w_{x}=\int _{0}^{\infty }\left( P^{\mathbb {C}^{\delta }}(x,x,t)-e^{-t}\right) \frac{dt}{t}.\nonumber \\ \end{aligned}$$

and observe that this quantity only depends on the vertex of $T^{\delta _{0}}$ corresponding to *x* in the discretization procedure, in particular in the vertex-transitive case this is just a constant. Assume first that there are no vertices of $\mathbb {C}^{\delta _{0}}$ on $\partial T^{\delta _{0}}$. Then, subdividing the vertices of $\Omega ^{\delta }$ into scaled copies of $T^{\delta _{0}}$, the fifth term above gives the leading term of the asymptotics (1.1):

4.3 $$\begin{aligned} d\sum _{x\in \Omega ^{\delta }}A_{x}=-A\cdot |\Omega ^{\delta }|\quad \text {where}\quad A:=-d\sum _{x\in T^{\delta _{0}}}A_{x}. \end{aligned}$$

If there are vertices of $T^{\delta _{0}}$ on $\partial T$ but not in its corners, then the contribution of those should be included in the definition of *A* with weights $\frac{1}{2}$. This leads to a miscount for the contribution of the vertices on $\partial \Omega$, which we absorb into the $|\partial \Omega ^{\delta }|$ term by re-defining the constants $B_{\mathcal {N}},B_{\mathcal {D}},$ cf. the second term in (6.8) below. Similarly, if there are vertices at the corners of $T^{\delta _{0}}$, they should be counted with weight $\frac{1}{6}$ or $\frac{1}{4}$, which leads to a miscount for boundary corners and cones which we absorb into $D_{p}$.

In the case there are punctures, in an *r*-neighborhood of a puncture *p*, we define $\Omega _{x}$ to be the punctured plane $\mathbb {C}^{\delta }{\setminus }\{p\}$, equipped with the connection $\varphi _{p}$ obtained by first restricting $\varphi$ to the neighborhood of *p* and then extending it to a flat connection on the whole $\mathbb {C}^{\delta }\setminus \{p\}.$ We then simply use $P^{\mathbb {C}^{\delta }\setminus \{p\},\varphi _{p}}$ instead of $d\cdot P^{\Omega ^{\delta }_{x}}$ in the above formulae. If there are vertices at the corners of *T* (and thus at the conical singularities), then the asymptotics of the heat kernel at a conical singularity *p* of angle $\alpha$ reads $P^{\Omega ^{\delta }_{p}}(p,p,t)=\frac{\alpha }{2\pi }e^{-w_{{\hat{p}}}t}\cdot \mathrm {Id}+O(t^{2}),$ where ${\hat{p}}$ is a corner of *T*; hence we should replace $P^{\mathbb {C}^{\delta }}(p,p,t)$ in the above formulae by $\frac{\alpha }{2\pi }\cdot P^{\mathbb {C}^{\delta }}({\hat{p}},{\hat{p}},t)$. This results in additional constant contributions to the asymptotics that can be absorbed into $D_{p}$.

## Contributions from the CLT Part

The goal for this section is to prove convergence of the first two terms in the key formula. We start with five fairly standard Lemmas, whose proof is deferred to Sect. 10. We denote $\Omega ^{\dagger }:=\partial \Omega \cup \mathcal {C}^{}\cup \mathcal {P}$

### Lemma 5.1

(Functional CLT). For any $T>0$, any $x\in \Omega \setminus \Omega ^{\dagger }$, any sequence $x^{\delta }\rightarrow x$, and any bounded, continuous function *f* on the space of paths $\gamma :[0,T]\rightarrow \Omega$ (equipped with sup-norm convergence), one has

$$\begin{aligned} \mathsf {\mathbb {E}}^{x^{\delta }}\left[ f(\gamma _{[0,\delta ^{-2}T]}^{\delta })\right] {\mathop {\longrightarrow }\limits ^{\delta \rightarrow 0}}\mathsf {\mathbb {E}}^{x}\left[ f(\gamma _{[0,T]})\right] . \end{aligned}$$

### Lemma 5.2

(Short time large diameter bound). For every $\varepsilon >0$, there are constants $C,c>0$ such that, for all $x\in \Omega ^{\delta }$, all $t>0$ and all $\delta <c$,

$$\begin{aligned} \mathbb {P}^{x}(\mathrm {diam}\,(\gamma _{[0,\delta ^{-2}t]}^{\delta })\ge \varepsilon )\le C\cdot t^{3}. \end{aligned}$$

### Lemma 5.3

(Uniform bound of the heat kernel). For each $\varepsilon >0$, there are constants $C,c>0$ such that, for all $\delta <c$,

5.1 $$\begin{aligned} P^{\Omega ^{\delta }}(x,y,\delta ^{-2}t)\le C\delta ^{2} \end{aligned}$$

whenever either $\mathrm {dist}(x,y)>\varepsilon ,$ or $t>\varepsilon$.

### Lemma 5.4

(Hölder regularity of heat kernels). There exists a number $\theta >0$ such that, for any $\eta >0$, there exists a constant $C_{\eta }$ with the following property: if $\mathrm {dist}(x,y)<\frac{1}{2}\mathrm {dist}(x,\Omega ^{\dagger })<\frac{1}{2}\eta$ and $t>\eta ^{2}$, then

5.2 $$\begin{aligned} \left| P^{\Omega ^{\delta },\varphi }(x,x,\delta ^{-2}t)-P^{\Omega ^{\delta },\varphi }(x,y,\delta ^{-2}t)\right| \le C_{\eta }\cdot |x-y|^{\theta }\cdot \delta ^{2}, \end{aligned}$$

where, to make sense of the left-hand side, we identify the vector spaces $V_{y},$ $y\in B(x,\frac{\eta }{2}),$ using a trivialization of $\varphi$ over $B(x,\frac{\eta }{2})$.

### Lemma 5.5

(Spectral gap). There are constants $C,c>0$ independent of $\delta$, such that for all $\delta$ small enough, one has

5.3 $$\begin{aligned} \left| \Theta ^{\Omega ^{\delta }}(\delta ^{-2}t)-k\right| <Ce^{-ct},\quad t\ge 1. \end{aligned}$$

We are in the position to prove convergence of the first two terms in the key formula:

### Lemma 5.6

We have the following convergence results:

5.4 $$\begin{aligned} \int _{1}^{\infty }\left( \Theta ^{\Omega ^{\delta },\varphi }(\delta ^{-2}t)-k\right) \frac{dt}{t}{\mathop {\longrightarrow }\limits ^{\text {as }\delta \rightarrow 0}}\int _{1}^{\infty }\left( \Theta ^{\Omega ,\varphi }(t)-k\right) \frac{dt}{t} \end{aligned}$$

and

5.5 $$\begin{aligned}&\int _{0}^{1}\sum _{x\in \Omega ^{\delta }}\left( \mathrm {Tr\,}P^{\Omega ^{\delta },\varphi }(x,x,\delta ^{-2}t)-d\cdot P^{\Omega ^{\delta }_{x}}(x,x,\delta ^{-2}t)\right) \frac{dt}{t}{\mathop {\longrightarrow }\limits ^{\text {as }\delta \rightarrow 0}}\nonumber \\&\quad \int _{0}^{1}\int _{\Omega }\left( \mathrm {Tr\,}P^{\Omega ,\varphi }(x,x,t)-d\cdot P^{\Omega _{x}}(x,x,t)\right) \,dx\frac{dt}{t}. \end{aligned}$$

### Proof

In view of Lemma 5.5, for (5.4), it suffices to prove the convergence

$$\begin{aligned} \int _{1}^{T}\Theta ^{\Omega ^{\delta },\varphi }(\delta ^{-2}t)\frac{dt}{t}{\mathop {\longrightarrow }\limits ^{\text {as }\delta \rightarrow 0}}\int _{1}^{T}\Theta ^{\Omega ,\varphi }(t)\frac{dt}{t}\,dx. \end{aligned}$$

for any fixed $T>0$. Let $0<\eta <\eta _{0}$, and let $\{\psi _{j}\}$ be a partition of unity for $\Omega$ such that $\mathrm {diam}(\mathrm {supp}\;\psi _{j})<\eta$ for all *j*. We write

$$\begin{aligned} \int _{1}^{T}\Theta ^{\Omega ^{\delta }}(\delta ^{-2}t)\frac{dt}{t}=\sum _{j}\int _{1}^{T}\sum _{x\in \Omega ^{\delta }}\psi _{j}(x)\mathrm {Tr\,}P^{\Omega ^{\delta },\varphi }(x,x,\delta ^{-2}t)\frac{dt}{t}\end{aligned}$$

and split the sum according to whether $j\in J:=\{j:\mathrm {dist}(\mathrm {supp}\;\psi _{j},\Omega ^{\dagger })>\eta _{0}\}$. By Lemma 5.3,

$$\begin{aligned} \sum _{j\notin J}\int _{1}^{T}\sum _{x\in \Omega ^{\delta }}\psi _{j}(x)\mathrm {Tr\,}P^{\Omega ^{\delta },\varphi }(x,x,\delta ^{-2}t)\frac{dt}{t}\le \sum _{\mathrm {dist}(x,\Omega ^{\dagger })\le 2\eta _{0}}\sum _{j}\psi _{j}(x)C\delta ^{2}\le C\mathcal {A}(\eta _{0}), \end{aligned}$$

where $\mathcal {A}(\eta _{0}){\mathop {\longrightarrow }\limits ^{\eta _{0}\rightarrow 0}}0$ is the area of $\{x\in \Omega :\mathrm {dist}(x,\Omega ^{\dagger })\le 2\eta _{0}\}$. For $j\in J$, we may apply Lemma 5.4 to any $x,y\in \mathrm {supp}\,\psi _{j}$. Averaging (5.2) with weights $\frac{\psi _{j}(x)\psi _{j}(y)}{S_{j}^{2}}$, where $S_{j}=\sum _{x\in \Omega ^{\delta }}\psi _{j}(x),$ multiplying by $t^{-1},$ and integrating yields

$$\begin{aligned}&\left| \int _{1}^{T}\sum _{x\in \Omega ^{\delta }}\psi _{j}(x)\mathrm {Tr\,}P^{\Omega ^{\delta },\varphi }(x,x,\delta ^{-2}t)\frac{dt}{t}-\int _{1}^{T}\frac{1}{S_{j}}\sum _{x,y\in \Omega ^{\delta }}\psi _{j}(x)\psi _{j}(y)\mathrm {Tr\,}P^{\Omega ^{\delta },\varphi }(x,y,\delta ^{-2}t)\frac{dt}{t}\right| \\&\quad \le C_{\eta _{0}}S_{j}\eta ^{\theta }\delta ^{2}. \end{aligned}$$

Summing these bounds over $j\in J$ yields the upper bound $C_{\eta _{0}}|\Omega ^{\delta }|\delta ^{2}\eta ^{\theta }$, which goes to zero uniformly in $\delta$ as $\eta \rightarrow 0$. Finally,

5.6 $$\begin{aligned} \int _{1}^{T}\frac{1}{S_{j}}\sum _{x,y\in \Omega ^{\delta }}\psi _{j}(x)\psi _{j}(y)\mathrm {Tr\,}P^{\Omega ^{\delta },\varphi }(x,y,\delta ^{-2}t)\frac{dt}{t}=\mathsf {\mathbb {E}}^{X}\left[ \int _{1}^{T}\mathrm {Tr\,}\varphi (\gamma _{[0,\delta ^{-2}t]}^{\delta })\psi _{j}(\gamma _{t}^{\delta })\frac{dt}{t}\right] , \nonumber \\ \end{aligned}$$

with the initial point *X* chosen at random with $\mathbb {P}(X=x)=\psi _{j}(x)/S_{j},$ and we pick a trivialization of $\varphi$ over $\mathrm {supp}\,\psi _{j}$. The expression inside the expectation is continuous with respect to the path $\gamma ^{\delta },$ therefore, by Lemma 5.1, (5.6) converges to its continuous counterpart

$$\begin{aligned} \mathsf {\mathbb {E}}^{X}\left[ \int _{1}^{T}\mathrm {Tr\,}\varphi (\gamma _{[0,t]})\psi _{j}(\gamma _{t})dt\right]&=\int _{1}^{T}\left( \int _{\Omega }\psi _{j}\right) ^{-1}\\&\quad \int _{x,y\in \Omega }\psi _{j}(x)\psi _{j}(y)\mathrm {Tr\,}P^{\Omega ,\varphi }(x,y,t)dxdy.=:I_{j} \end{aligned}$$

In view of the bounds we have collected, we have that

$$\begin{aligned} \int _{1}^{T}\Theta ^{\Omega ^{\delta },\varphi }(\delta ^{-2}t)\frac{dt}{t}{\mathop {\longrightarrow }\limits ^{\text {as }\delta \rightarrow 0}}\lim _{\eta _{0}\rightarrow 0}\lim _{\eta \rightarrow 0}\sum _{j\in J}I_{j}. \end{aligned}$$

Since the continuous heat kernels satisfy the suitable counterparts of Lemmas 5.3–5.4 (for instance, as a consequence of the discrete bounds and the convergence), an argument as above gives that the latter quantity is equal to $\int _{1}^{T}\Theta ^{\Omega ,\varphi }(t)\frac{dt}{t},$ as required.

For (5.5), the same argument as above, applied to $\Omega ^{\delta }$ and each of $\Omega ^{\delta }_{x},$ gives the convergence of the integral from $t_{0}$ to 1, for any fixed $t_{0}>0.$ Hence, it suffices to show that the integral from 0 to $t_{0}$ converges to 0 as $t_{0}\rightarrow 0$ uniformly in $\delta$. We can write

$$\begin{aligned} \mathrm {Tr\,}P^{\Omega ^{\delta },\varphi }(x,x,\delta ^{-2}t)-&d&\cdot P^{\Omega ^{\delta }_{x}}(x,x,\delta ^{-2}t)\\&\qquad =\mathsf {\mathbb {E}}^{x}\left[ \mathrm {Tr\,}\varphi (\gamma _{[0,\delta ^{-2}t]})\mathbb {I}_{\gamma _{\delta ^{-2}t}=x}-d\cdot \mathbb {I}_{{\hat{\gamma }}_{\delta ^{-2}t}=x}\right] , \end{aligned}$$

where $\gamma$ and ${\hat{\gamma }}$ are random walks on $\Omega ^{\delta }$ and $\Omega ^{\delta }_{x}$, respectively, coupled in such a way that they coincide up until $\tau _{r}:=\min \{s:\mathrm {dist}(\gamma _{\delta ^{-2}s},x)\ge r\}.$ On the event $\tau _{r}\ge t$, the expression in the expectation is zero. Hence, we can write

$$\begin{aligned}&\left| \mathrm {Tr\,}P^{\Omega ^{\delta },\varphi }(x,x,\delta ^{-2}t)-d\cdot P^{\Omega ^{\delta }_{x}}(x,x,\delta ^{-2}t)\right| \\&\quad \le d\cdot \mathbb {P}(\tau _{r}<t)\left( \sup _{s<t,|y-x|\ge r}P^{\Omega ^{\delta }}(y,x,\delta ^{-2}s)+\sup _{s<t,|y-x|\ge r}P^{\Omega _{x}^{\delta }}(y,x,\delta ^{-2}s)\right) \\&\quad \le Cd\cdot \mathbb {P}(\tau _{r}<t)\delta ^{2}\le C't^{3}\delta ^{2}. \end{aligned}$$

where we have used Lemma 5.3 and then Lemma 5.2. Summing over *x* gives a bound on the integrand in the left-hand side of (5.5) that is independent of $\delta$ and integrable at 0. This concludes the proof. $\square$

## Local Contributions

In this section, we compute the asymptotics of the local term $\sum _{x\in \Omega ^{\delta }}I_{\mathbb {C}^{\delta }}^{\Omega _{x}^{\delta }}(x)$ in (4.1), where

6.1 $$\begin{aligned} I_{\mathbb {C}^{\delta }}^{\Omega _{x}^{\delta }}(x)=\int _{0}^{\infty }\left( P^{\Omega ^{\delta }_{x}}\left( x,x,t\right) -P^{\mathbb {C}^{\delta }}\left( x,x,t\right) \right) \frac{dt}{t} \end{aligned}$$

Each of the model surfaces $\Lambda =\mathbb {H},\Upsilon ^{\alpha },\mathcal {C}^{\alpha }$ has scaling acting on it, and $I_{\mathbb {C}^{\delta }}^{\Lambda ^{\delta }}(x)=I_{\mathbb {C}^{\delta _{0}}}^{\Lambda ^{\delta _{0}}}(N\cdot x).$ Thus, decreasing $\delta$ by going from *N* to $N+1$ is tantamount to adding new terms to the sum, corresponding to those *x* whose distance to a conical singularity, the boundary, or a puncture is between $r\frac{N}{N+1}$ and *r*. The asymptotics of those new terms is governed by Central limit theorem. We postpone the proof of the following Lemmas to Sect. 10:

### Lemma 6.1

(Local CLT with error bound). If $\Lambda$ is one of the model surfaces, $\Lambda ^{\delta }$ its discretization, and $\varepsilon >0$, then there exist $q>0$ and $C>0$ such that

$$\begin{aligned} \left| \delta ^{-2}\cdot P^{\Lambda ^{\delta }}(x,y,\delta ^{-2}t)-P^{\Lambda }(x,y,t)\right| \le C\delta ^{q}\cdot \max \{t^{-1},1\}, \end{aligned}$$

for all $\delta ,$ all $t\in (\delta ^{q},\delta ^{-q})$ and *x*, *y* at distance at least $\varepsilon$ from the tip (if $\Lambda$ is a wedge or a cone).

### Lemma 6.2

(Uniform tail bound for the heat kernel). If $\Lambda ^{\delta }$ is one of the model surfaces, then there exists $C>0$ such that, for any $\delta ,t>0$ and $x,y\in \Lambda ^{\delta }$,

$$\begin{aligned} P^{\Lambda ^{\delta }}(x,y,\delta ^{-2}t)\le C\delta ^{2}t^{-1}. \end{aligned}$$

Let $\Lambda _{1}\ni x_{0}$ be a continuous model surface equipped with boundary conditions. Let $\Lambda _{2}$ be another model surface that contains an isometric copy $B'(x_{0},\eta )$ of the ball $B(x_{0},\eta )\subset \Lambda _{1}$ not containing tips of a wedge or a cone, with corresponding parts of the boundary having the same boundary conditions, and let $\Lambda _{1,2}^{\delta }$ be their discretizations that respect the isometry. We will denote, for $x\in B(x_{0},\eta ),$

$$\begin{aligned} I_{\Lambda _{1}}^{\Lambda _{2}}(x):=\int _{0}^{\infty }\left( P^{\Lambda _{2}}\left( x,x,t\right) -P^{\Lambda _{1}}\left( x,x,t\right) \right) \frac{dt}{t}, \end{aligned}$$

where we identify the points in $B(x_{0},\eta )$ with their isomorphic copies. We use a similar notation for discretizations $\Lambda _{1,2}^{\delta }$ of $\Lambda _{1,2}$ We have the following Lemma:

### Corollary 6.3

In the above setup, there exist $\rho >0$ and $C>0$ such that

$$\begin{aligned} \left| \delta ^{-2}I_{\Lambda _{1}^{\delta }}^{\Lambda _{2}^{\delta }}(x)-I_{\Lambda _{1}}^{\Lambda _{2}}(x)\right| \le C\delta ^{\rho }; \end{aligned}$$

for all $\delta$ and all $x\in B(x_{0},\frac{\eta }{2}).$

### Proof

Let *q* be as in Lemma 6.1. At small times $t\le \delta ^{q/2}$, we repeat the argument in the end of the proof of Lemma 5.6 for $d=1$ and $\Lambda _{1,2}^{\delta }$ instead of $\Omega ^{\delta },\Omega ^{\delta }_{x}$; this gives

$$\begin{aligned} \left| P^{\Lambda _{2}^{\delta }}\left( x,x,\delta ^{-2}t\right) -P^{\Lambda _{1}^{\delta }}\left( x,x,\delta ^{-2}t\right) \right| \le C\delta ^{2}t^{3},\quad t\le \delta ^{q/2}, \end{aligned}$$

and, integrating,

$$\begin{aligned} \left| \int _{0}^{\delta ^{q/2}}\left( P^{\Lambda _{2}^{\delta }}\left( x,x,\delta ^{-2}t\right) -P^{\Lambda _{1}^{\delta }}\left( x,x,\delta ^{-2}t\right) \right) \frac{dt}{t}\right| \le C\delta ^{2}\cdot \delta ^{3q/2}. \end{aligned}$$

At large times $t>\delta ^{-q}$, we use Lemma 6.2 to get

$$\begin{aligned} \left| \int _{\delta ^{-q}}^{\infty }\left( P^{\Lambda _{2}^{\delta }}\left( x,x,\delta ^{-2}t\right) -P^{\Lambda _{1}^{\delta }}\left( x,x,\delta ^{-2}t\right) \right) \frac{dt}{t}\right| \le 2C\delta ^{2}\int _{\delta ^{-q}}^{\infty }t^{-2}dt\le 2C\delta ^{2+q}. \end{aligned}$$

Clearly, similar estimates, with right-hand side divided by $\delta ^{2},$ hold for continuous heat kernels, e.g., as a consequence of convergence. At intermediate times $\delta ^{\frac{q}{2}}<t<\delta ^{-q}$, we apply Lemma 6.1 to each of $\Lambda _{1,2}$ separately to get

$$\begin{aligned}&\int _{\delta ^{q/2}}^{\delta ^{-q}}\left| \delta ^{-2}P^{\Lambda _{1,2}^{\delta }}\left( x,x,\delta ^{-2}t\right) -P^{\Lambda _{1,2}}\left( x,x,\delta ^{-2}t\right) \right| \frac{dt}{t}\\&\qquad \le C\delta ^{q}\int _{\delta ^{q/2}}^{\delta ^{-q}}\frac{dt}{t}\max \{t^{-1},1\}\le 2Cq\delta ^{q}(\log \delta ^{-1}+\delta ^{-\frac{q}{2}})\le {\hat{C}}\delta ^{\frac{q}{2}}. \end{aligned}$$

Combining all the estimates above yields the result. $\square$

### Corollary 6.4

We have

$$\begin{aligned} \sum _{x\in \Omega ^{\delta }}\int _{\delta ^{-2}}^{\infty }P^{\Omega _{x}^{\delta }}(x,x,t)\frac{dt}{t}{\mathop {\longrightarrow }\limits ^{\delta \rightarrow 0}}\int _{\Omega }\int _{1}^{\infty }P^{\Omega _{x}}(x,x,t)\frac{dt}{t}. \end{aligned}$$

### Proof

As in the proof of Lemma 5.6, we change the variable $t\rightarrow \delta ^{-2}t$. We then use Lemma 5.3 ensure that the sum and the integral over the $\eta _{0}$-neighborhood of the boundary and singularities tends to zero with $\eta _{0},$ uniformly in $\delta .$ This allows to consider the sum over the complement of that neighborhood only. In that region, we argue as in the proof of Corollary 6.3 that the summand converges to the integrand uniformly: apply Lemma 6.1 on the integral from 1 to $\delta ^{-q}$ and Lemma 6.2 to the integral from $\delta ^{-q}$ to infinity. The only difference with the proof of Corollary 6.3 is that now we do not need to deal with small *t*. $\square$

### Conical singularities

Let us compute the contribution of an *r*-neighborhood of the tip of a conical singularity with angle $\alpha$ to (6.1). Changing the scale to $\delta _{0}$, we see that

$$\begin{aligned} \sum _{x\in \mathcal {C}^{\alpha ,\delta }:|x|\le r}I_{\mathbb {C}^{\delta }}^{\mathcal {C}^{\alpha ,\delta }}(x)=\sum _{x\in \mathcal {C}^{\alpha ,\delta _{0}}:|x|\le rN}I_{\mathbb {C}^{\delta _{0}}}^{\mathcal {C}^{\alpha ,\delta _{0}}}(x), \end{aligned}$$

where $|\cdot |$ denotes the distance to the tip; that is, decreasing $\delta$ for a fixed *r* simply results in adding new terms to the sum. The asymptotics of those terms as $|x|\rightarrow \infty$ can be read off Corollary 6.3: if $rN\le |x|<r(N+1),$ then

$$\begin{aligned} I_{\mathbb {C}^{\delta _{0}}}^{\mathcal {C}^{\alpha ,\delta _{0}}}(x)=I_{\mathbb {C}^{\delta }}^{\mathcal {C}^{\alpha ,\delta }}\left( \frac{x}{N}\right) =\delta ^{2}\cdot I_{\mathbb {C}}^{\mathcal {C}^{\alpha }}\left( \frac{x}{N}\right) +O(\delta ^{2+\rho })=I_{\mathbb {C}}^{\mathcal {C}^{\alpha }}\left( 1\right) \cdot \delta _{0}^{2}|x|^{-2}+O(|x|^{-2-\rho }), \end{aligned}$$

where 1 is any point at distance 1 from the tip, and we used rotational invariance of $I_{\mathbb {C}}^{\mathcal {C}^{\alpha }}(ax),$ Brownian scaling $I_{\mathbb {C}}^{\mathcal {C}^{\alpha }}(ax)=a^{-2}I_{\mathbb {C}}^{\mathcal {C}^{\alpha }}(x)$, and the relation $\delta N=\delta _{0}.$ The error term $O(|x|^{-2-\rho })$ sums to a constant over $\mathcal {C}^{\alpha ,\delta _{0}},$ and recalling that $\mathbb {C}^{\delta _{0}}$ has $\delta _{0}^{-2}$ vertices per unit area, we have

$$\begin{aligned} \sum _{x\in \mathcal {C}^{\alpha ,\delta _{0}}:|x|\le rN}\delta _{0}^{2}|x|^{-2}=\int _{x\in \mathcal {C}^{\alpha }:1\le |x|\le rN}|x|^{-2}+{\check{D}}_{\alpha }+o(1)=\alpha \cdot \log (rN)+{\check{D}}_{\alpha }+o(1). \end{aligned}$$

Taking into account that $\log (rN)=\log r+\log \delta _{0}-\log \delta$, we conclude

6.2 $$\begin{aligned} \sum _{x\in \mathcal {C}^{\alpha ,\delta _{0}}:|x|\le rN}I_{\mathbb {C}^{\delta _{0}}}^{\mathcal {C}^{\alpha ,\delta _{0}}}(x)=-\alpha \cdot I_{\mathbb {C}}^{\mathcal {C}^{\alpha }}(1)\cdot \log \delta +{\hat{D}}_{\alpha }+o(1), \end{aligned}$$

where ${\hat{D}}_{\alpha }$ is a (lattice-dependent) constant.

### Boundary segments

Let $l\subset {{\partial }{{\Omega }}}$ be a side of a triangle or a square comprising $\Omega ;$ we introduce a local coordinate in which *l* is identified with $(0;1)\subset \partial \mathbb {H}$. Let $l^{\delta }$ be the corresponding segment of $\partial \Omega ^{\delta }$. Let $\alpha _{0,1}$ be the angles of the wedges at its endpoints 0 and 1, and denote ${\hat{\alpha }}_{0,1}:=\min \{\alpha _{0,1}/2;\pi /2\}$. We consider the contribution to (6.1) of the points that are at distance at most *r* from $l^{\delta }$, but at the distance greater than $r_{\alpha _{0,1}}=r/\sin ({\hat{\alpha }}_{0,1})$ from its endpoints 0 and 1, respectively. This contribution reads

6.3 $$\begin{aligned} \sum _{x\in R_{r}^{\delta }}I_{\mathbb {C}^{\delta }}^{\mathbb {H}^{\delta }}(x)-\sum _{x\in \Gamma _{0}^{\delta }}I_{\mathbb {C}^{\delta }}^{\mathbb {H}^{\delta }}(x)-\sum _{x\in \Gamma _{1}^{\delta }}I_{\mathbb {C}^{\delta }}^{\mathbb {H}^{\delta }}(x)-\sum _{x\in Y_{0}^{\delta }}I_{\mathbb {C}^{\delta }}^{\mathbb {H}^{\delta }}(x)-\sum _{x\in Y_{1}^{\delta }}I_{\mathbb {C}^{\delta }}^{\mathbb {H}^{\delta }}(x), \end{aligned}$$

where

$$\begin{aligned}&R_{r}^{\delta } =\{x\in \mathbb {H}^{\delta }:\Im \mathfrak {m}\,x\le r;0\le \Re \mathfrak {e}\,x<1\};\\&Y_{0}^{\delta } =\{x\in \mathbb {H}^{\delta }:|x|<r_{\alpha _{0}};0<\mathrm {arg}x\le {\hat{\alpha }}_{0}\};\\&Y_{1}^{\delta } =\{x\in \mathbb {H}^{\delta }:|x-1|<r_{\alpha _{1}};\pi -{\hat{\alpha }}_{1}\le \mathrm {arg}(x-1)<\pi \};\\&\Gamma _{0}^{\delta } =\{x\in \mathbb {H}^{\delta }:\Im \mathfrak {m}\,x<r;{\hat{\alpha }}_{0}<\mathrm {arg}x\le \pi /2\};\\&\Gamma _{1}^{\delta } =\{x\in \mathbb {H}^{\delta }:\Im \mathfrak {m}\,x<r;\pi /2<\mathrm {arg}(x-1)\le \pi -{\hat{\alpha }}_{1}\}, \end{aligned}$$

see Fig. 3; the boundary conditions in $\mathbb {H}^{\delta }$ above are inherited from $l^{\delta }$. We first treat the sum over $R_{r}^{\delta },$ which we can split into $N=\delta _{0}\delta ^{-1}$ strips $R_{r}^{\delta }(k):=\{\Im \mathfrak {m}\,x\le r,\frac{k}{N}\le \Re \mathfrak {e}\,x<\frac{k+1}{N}\}$ that all give equal contributions. As in the cone case, we see that decreasing $\delta$ is tantamount to adding new terms to the sum over $R_{r}^{\delta }(0)$, i.e.,

$$\begin{aligned} \sum _{x\in R_{r}^{\delta }(0)}I_{\mathbb {C}^{\delta }}^{\mathbb {H}^{\delta }}(x)= & {} \sum _{x\in R_{rN}^{\delta _{0}}(0)}I_{\mathbb {C}^{\delta _{0}}}^{\mathbb {H}^{\delta _{0}}}(x)={\hat{B}}-I_{\mathbb {C}}^{\mathbb {H}}(i) \\&\cdot \sum _{x\in R_{\infty }^{\delta _{0}}(0)\setminus R_{rN}^{\delta _{0}}(0)}\delta _{0}^{2}\left( (\Im \mathfrak {m}\,x)^{-2}+O((\Im \mathfrak {m}\,x)^{-2-\rho })\right) \\= & {} {\hat{B}}-I_{\mathbb {C}}^{\mathbb {H}}(i)\cdot \left( (rN)^{-1}+O(N^{-1-\rho })\right) \end{aligned}$$

where ${\hat{B}}=\sum _{x\in R_{\infty }^{\delta _{0}}(0)}I_{\mathbb {C}^{\delta _{0}}}^{\mathbb {H}^{\delta _{0}}}(x)$ is a lattice-dependent constant, so that

$$\begin{aligned} \sum _{x\in R_{r}^{\delta }}I_{\mathbb {C}^{\delta }}^{\mathbb {H}^{\delta }}(x)={\hat{B}}\cdot N-I_{\mathbb {C}}^{\mathbb {H}}(i)r^{-1}+O(\delta ^{\rho }), \end{aligned}$$

where we can compute, with the sign $s=\pm 1$ depending on the boundary conditions as $s_{\mathcal {N}}=+1$ and $s_{\mathcal {D}}=-1$,

$$\begin{aligned} I_{\mathbb {C}}^{\mathbb {H}}(i):=\,&\int _{0}^{\infty }\left( P^{\mathbb {H}}\left( i,i,t\right) -P^{\mathbb {C}}\left( i,i,t\right) \right) \frac{dt}{t}=s\cdot \int _{0}^{\infty }P^{\mathbb {C}}\left( i,-i,t\right) \frac{dt}{t}\\ =&s\cdot \int _{0}^{\infty }\frac{1}{2\pi t}e^{-\frac{2}{t}}\frac{dt}{t}=s\cdot \frac{1}{4\pi }, \end{aligned}$$

The contributions of $Y_{0,1}^{\delta }$ to (6.3) will cancel the corresponding contributions from corners, thus we will leave them as they are for a while. The contribution of $\Gamma _{0,1}^{\delta }$ is computed as in the cone case, applying Corollary 6.3 and then using Brownian scaling and shift invariance of $I_{\mathbb {C}}^{\mathbb {H}}(i):$

$$\begin{aligned} \sum _{y\in \Gamma _{0}^{\delta }}I_{\mathbb {C}^{\delta }}^{\mathbb {H}^{\delta }}(y)&=s\cdot \sum _{\begin{array}{c} 0\le \Im x\le rN;\\ {\hat{\alpha }}_{0}<\mathrm {arg}x<\pi /2 \end{array} }(\Im \mathfrak {m}\,x)^{-2}\delta _{0}^{2}\left( I_{\mathbb {C}}^{\mathbb {H}}(i)+O(\Im \mathfrak {m}\,x^{-\rho })\right) \\&=s\cdot \frac{\cot ({\hat{\alpha }}_{0})}{4\pi }\log \delta +{\hat{D}}_{\alpha _{0}}+o(1), \end{aligned}$$

and similarly

$$\begin{aligned} \sum _{y\in \Gamma _{1}^{\delta }}I_{\mathbb {C}^{\delta }}^{\mathbb {H}^{\delta }}(y)=s\cdot \frac{\cot ({\hat{\alpha }}_{1})}{4\pi }\log \delta +{\hat{D}}_{\alpha _{1}}+o(1). \end{aligned}$$

### Boundary corners

We parameterize a boundary corner $\Upsilon ^{\alpha }$ by a local coordinate *z* so that $\Upsilon ^{\alpha }=\{z\in \mathbb {C}:0<\mathrm {arg}z<\alpha \},$ and denote we denote by $Y_{\text {left}}^{\delta }$ (respectively, $Y_{\text {right}}^{\delta }$) the set $Y_{1}^{\delta }$ (respectively, $Y_{0}^{\delta }$) corresponding to the boundary segment adjacent to $\Upsilon ^{\alpha }$ on the left (respectively, on the right). We also denote $Y_{\mathrm {middle}}^{\delta }:=\{x\in \Upsilon ^{\alpha ,\delta }:|x|\le r_{\alpha }\}\setminus \left( Y_{\text {left}}^{\delta }\cup Y_{\text {right}}^{\delta }\right)$, which is non-empty if and only if $\alpha >\pi$. The contribution of $\Upsilon ^{\alpha }$ to (6.1) can be written as

6.4 $$\begin{aligned} \sum _{x:|x|<r_{\alpha }}I_{\mathbb {C}^{\delta }}^{\Upsilon ^{\alpha ,\delta }}(x)= & {} \sum _{x\in Y_{\mathrm {middle}}^{\delta }}I_{\mathbb {C}^{\delta }}^{\Upsilon ^{\alpha ,\delta }}(x)+\sum _{x\in Y_{\text {right}}^{\delta }}I_{\mathbb {H}^{\delta }}^{\Upsilon ^{\alpha ,\delta }}(x)+\sum _{x\in Y_{\text {left}}^{\delta }}I_{{\hat{\mathbb {H}}}^{\delta }}^{\Upsilon ^{\alpha ,\delta }}(x)\nonumber \\&+\,\sum _{x\in Y_{\text {right}}^{\delta }}I_{\mathbb {C}^{\delta }}^{\mathbb {H}^{\delta }}(x)+\sum _{x\in Y_{\text {left}}^{\delta }}I_{\mathbb {C}^{\delta }}^{{\hat{\mathbb {H}}}^{\delta }}(x). \end{aligned}$$

where ${\hat{\mathbb {H}}}^{\delta }$ stands for the upper-half plane $\mathbb {H}^{\delta }$ rotated counterclockwise by $\alpha -\pi$ around $\pi$ (so that its boundary coincides with the *left* boundary of the corner), and the boundary conditions $\Upsilon ^{\alpha ,\delta },\mathbb {H}^{\delta },{\hat{\mathbb {H}}}^{\delta }$ are inherited from those in $\Omega ^{\delta }$. The first three terms yield, similarly to the computations above,

$$\begin{aligned} -{\hat{C}}_{\alpha }^{b{\hat{b}}}\cdot \log \delta +{\hat{D}}_{\alpha }^{b{\hat{b}}}+o(1), \end{aligned}$$

where

6.5 $$\begin{aligned} {\hat{C}}_{\alpha }^{b{\hat{b}}}=\int _{0}^{{\hat{\alpha }}}I_{\mathbb {H}_{b}}^{\Upsilon ^{\alpha }}(e^{i\theta })\,d\theta +\int _{\alpha -{\hat{\alpha }}}^{\alpha }I_{{\hat{\mathbb {H}}}_{{\hat{b}}}}^{\Upsilon ^{\alpha }}(e^{i\theta })\,d\theta +\mathbf {1}_{\alpha >\pi }\int _{\pi /2}^{\alpha -\pi /2}I_{\mathbb {C}}^{\Upsilon ^{\alpha }}(e^{i\theta })\,d\theta , \end{aligned}$$

${\hat{D}}_{\alpha }^{b{\hat{b}}}$ are constants and $b,{\hat{b}}\in \{\mathcal {D},\mathcal {N}\}$ are boundary conditions on $\mathbb {H},{\hat{\mathbb {H}}}.$

Observe that when collecting the contributions to (6.1) along $\partial \Omega ^{\delta }$, the last two terms in (6.4) cancel out the corresponding terms in (6.3). The total contribution of the *r*-neighborhood of $\partial \Omega ^{\delta }$ to (6.1) is therefore

6.6 $$\begin{aligned}&{\hat{B}}_{\mathcal {D}}\cdot N\cdot |\partial _{\mathcal {D}}\Omega |+{\hat{B}}_{\mathcal {N}}\cdot N\cdot |\partial _{\mathcal {N}}\Omega |-I_{\mathbb {C}}^{\mathbb {H}_{\mathcal {D}}}(i)r^{-1}|\partial _{\mathcal {D}}\Omega |-I_{\mathbb {C}}^{\mathbb {H}_{\mathcal {N}}}(i)r^{-1}|\partial _{\mathcal {N}}\Omega |\nonumber \\&\quad -\left( \sum _{p\in \Upsilon }C_{p}\right) \cdot \log \delta +\sum _{i\in \text {Corners}}{\hat{D}}_{\alpha _{i}}^{b_{i}b_{i+1}}+o(1), \end{aligned}$$

where $C_{p}$ depends only on the angle and the boundary conditions $b,{\hat{b}}\in \{\mathcal {D},\mathcal {N}\}$ on the segments adjacent to the $p\simeq \Upsilon _{b{\hat{b}}}^{\alpha }$ as

6.7 $$\begin{aligned} C_{\Upsilon _{b{\hat{b}}}^{\alpha }}={\hat{C}}_{\alpha }^{b{\hat{b}}}-(s_{b}+s_{{\hat{b}}})\cdot \frac{\cot (\min \{\frac{\alpha }{2};\frac{\pi }{2}\})}{4\pi }. \end{aligned}$$

Taking into account the discussion at the end of Sect. 4, we have

6.8 $$\begin{aligned} B_{\mathcal {D},\mathcal {N}}=-d\cdot \left( {\hat{B}}_{\mathcal {D},\mathcal {N}}{\mp }\frac{1}{2}\sum _{x\in T^{\delta _{0}}\cap [0,1)}A_{x}\right) , \end{aligned}$$

where the sum is over the vertices in one boundary edge of the fundamental domain $T^{\delta _{0}}.$

### Punctures

Using a suitable modification of Lemma 6.1, similarly to the conical singularity case, we have, in the local coordinate where $p=0,$

6.9 $$\begin{aligned} \sum _{x\in \mathbb {C}^{\delta }:|x|\le r}I_{\mathbb {C}^{\delta }}^{\mathbb {C}^{\delta }\setminus \{0\},\varphi _{p}}(x)&=I_{\mathbb {C}}^{\mathbb {C}\setminus \{0\},\varphi _{p}}(1)\cdot \sum _{x\in \mathbb {C}^{\delta _{0}}:|x|\le rN}\delta _{0}^{2}\left( |x|^{-2}+O(|x|^{-2-\rho })\right) \nonumber \\&=-2\pi I_{\mathbb {C}}^{\mathbb {C}\setminus \{0\},\varphi _{p}}(1)\log \delta +D_{\varphi _{p}}+o(1). \end{aligned}$$

## Explicit Computations for the Logarithmic Term

In this section, we compute the integrals involving heat kernels that contribute to the logarithmic term of the asymptotics. The results are not new. Namely, as pointed out in [18, 21], the constant *C* in (1.1) is related to the spectral zeta-function by $C=-2\zeta _{\Omega }(0),$ see Remark 9.1. The value of $\zeta _{\Omega }(0)$ has been computed in a much greater generality by Cheeger, see [6, Theorem 4.4] and the discussion thereafter.

Here, we propose an alternative computation based on the following identity for the heat kernel on the universal cover of a punctured plane:

### Lemma 7.1

We have, for the heat kernel $\tilde{P}:=P^{\widetilde{\mathbb {C}\setminus \{0\}}}$,

$$\begin{aligned} \int _{0}^{\infty }\tilde{P}(1,e^{i\alpha },t)\cdot \frac{dt}{t}=\frac{1}{\pi \alpha ^{2}}. \end{aligned}$$

### Proof

We use the Brownian loop measure of Lawler and Werner, see [35] or [33, Section 5.6], defined as a $\sigma$-finite measure on the space of unrooted closed loops in a Riemann surface $\Lambda$ by

7.1 $$\begin{aligned} \mu _{\Lambda }=\int _{0}^{\infty }\frac{1}{t}P^{\Lambda }(z,z,t)\mu _{\Lambda ,z,t}^{\sharp }\,|dz|^{2}dt, \end{aligned}$$

where $P^{\Lambda }$ is the heat kernel in $\Lambda$ with Dirichlet boundary conditions, and $\mu _{\Lambda ,z,t}^{\sharp }$ is the Brownian probability measure on the paths from *z* to *z* of duration *t*. We will only need the conformal invariance of this measure, see [35, Proposition 6] which is stated for planar domains, but the proof, being a local computation, extends verbatim to Riemann surfaces. Consider the annular region $\mathcal {A}_{r,\alpha }=\{r\le |z|\le 1\}/\{z\sim e^{i\alpha }z\}$ in the cone of angle $\alpha$, $\tilde{\mathcal {A}}_{r,\alpha }$ its universal cover, and let *E* denote the set of loops in $\mathcal {A}_{r,\alpha }$ that wind around the annulus once counterclockwise. The map $\phi :z\mapsto -i\log z$ maps ${{\mathcal {A}_{r,\alpha }}}$ onto the cylinder $\mathcal {O}_{\alpha ,r}=\mathcal {S}_{r}/\{z\sim z+\alpha \},$ where $\mathcal {S}_{r}=\{0\le \Im \mathfrak {m}\,z\le -\log r\},$ so, by the conformal invariance, we have

7.2 $$\begin{aligned}&\int _{\mathcal {A}_{r,\alpha }}\int _{0}^{\infty }P^{\mathcal {\tilde{A}}_{r,\alpha }}(z,ze^{i\alpha },t)|dz|^{2}\frac{dt}{t}=\mu _{\mathcal {A}_{r,\alpha }}(E)\nonumber \\&\quad =\mu _{\mathcal {O}_{\alpha ,r}}(\phi (E))=\int _{\begin{array}{c} \{0\le \Re \mathfrak {e}\,z<\alpha \}\cap S_{r} \end{array} }\int _{0}^{\infty }P^{\mathcal {S}_{r}}(z,z+\alpha ,t)|dz|^{2}\frac{dt}{t}. \end{aligned}$$

Note that by scaling invariance, the total $\mu _{\mathcal {C}^{\alpha }}$ measure of the loops that wind around $\mathcal {C}^{\alpha }$ and intersect a given circle $|z|=r$ does not depend on *r*. We claim that it is also finite. Indeed, by conformal invariance, we can pass to the cylinder $\mathcal {O}_{\alpha }=\mathbb {C}/\{z\sim z+\alpha \}$, when the circle is mapped to $l_{h}:=\{w:\Im \mathfrak {m}\,w=h\},$ and then use that for some $c,C>0,$

$$\begin{aligned} \mu _{\mathcal {O}_{\alpha },z,t}^{\sharp }(\{\gamma :\gamma \cap l_{h}\ne \emptyset )\le Ce^{-c\frac{(\Im \mathfrak {m}\,z-h)^{2}}{t}}. \end{aligned}$$

Also, $P^{\mathcal {O}_{\alpha }}(z,z,t)\sim Ct^{-\frac{1}{2}}$ as $t\rightarrow \infty$. These two bounds imply that the contribution to (7.1) from the points *z* with $|\Im \mathfrak {m}\,z-h|>1$ is finite. Since the probability that a bridge with a small *t* winds around $\mathcal {O}_{\alpha }$ is exponentially small, the region $|\Im \mathfrak {m}\,z-h|\le 1$ also gives a finite contribution.

Hence, up to *O*(1) as $r\rightarrow 0$, the left-hand side of (7.2) equals

$$\begin{aligned} \int _{\mathcal {A}_{r,\alpha }}\int _{0}^{\infty }\tilde{P}(z,ze^{i\alpha },t)\frac{dt}{t}= & {} \int _{\mathcal {A}_{r,\alpha }}|z|^{-2}\int _{0}^{\infty }\tilde{P}(1,e^{i\alpha },t)\frac{dt}{t}\\= & {} -\alpha \log r\int _{0}^{\infty }\tilde{P}(1,e^{i\alpha },t)\frac{dt}{t}. \end{aligned}$$

We conclude by comparing this to the right-hand side of (7.2), which is, up to *O*(1), 

$$\begin{aligned} \int _{\begin{array}{c} \{0\le \Re \mathfrak {e}\,z<\alpha \}\cap S_{r} \end{array} }\int _{0}^{\infty }P^{\mathbb {C}}(z,z+\alpha ,t)\frac{dt}{t}=-\alpha \log r\int _{0}^{\infty }\frac{1}{2\pi t}e^{-\frac{\alpha ^{2}}{2t}}\frac{dt}{t}=-\frac{\log r}{\pi \alpha }. \end{aligned}$$

$$\square $$

Since $\widetilde{\mathbb {C}\setminus \{0\}}$ also covers each of the cones $\mathcal {C}^{\alpha }\backsimeq \mathbb {C}/\{z\sim e^{i\alpha }z\}$, we have

$$\begin{aligned} P^{\mathcal {C}^{\alpha }}\left( x,y,t\right) =\sum _{k\in \mathbb {Z}}\tilde{P}(x,ye^{ik\alpha },t). \end{aligned}$$

We now can compute, using that $\sum _{k=1}^{\infty }1/k^{2}=\frac{\pi ^{2}}{6}$,

7.3 $$\begin{aligned} I_{\mathbb {C}}^{\mathcal {C}^{\alpha }}(1)&=\int _{0}^{\infty }\left( \sum _{k\in \mathbb {Z}}\tilde{P}(1,e^{\alpha ik},t)-\tilde{P}(1,e^{2\pi ik},t)\right) \frac{dt}{t}\nonumber \\&=\sum _{k\in \mathbb {Z}\setminus \{0\}}\left( \frac{1}{\pi (\alpha k)^{2}}-\frac{1}{\pi (2\pi k)^{2}}\right) =\frac{\pi }{3\alpha ^{2}}-\frac{1}{12\pi } \end{aligned}$$

By the reflection principle, we have $P^{\Upsilon ^{\alpha }}\left( x,y,t\right) =P^{\mathcal {C}^{2\alpha }}\left( x,y,t\right) \pm P^{\mathcal {C}^{2\alpha }}\left( x,{\bar{y}},t\right)$ for $\Upsilon ^{\alpha }=\Upsilon _{\mathcal {N}}^{\alpha }$ and $\Upsilon ^{\alpha }=\Upsilon _{\mathcal {D}}^{\alpha }$ respectively, and hence, using that $\sum _{k\in \mathbb {Z}}(x+k)^{-2}=\pi ^{2}\sin ^{-2}\pi x$, we get

7.4 $$\begin{aligned}&\int _{0}^{\frac{\alpha }{2}}I_{\mathbb {H}}^{\Upsilon ^{\alpha }}(e^{i\theta })d\theta \nonumber \\&\quad =\int _{0}^{\frac{\alpha }{2}}\sum _{k\in \mathbb {Z}\setminus \{0\}}\left( \frac{1}{\pi (2\alpha k)^{2}}-\frac{1}{\pi (2\pi k)^{2}}\pm \frac{1}{\pi (2\theta +2\alpha k)^{2}}{\mp }\frac{1}{\pi (2\theta +2\pi k)^{2}}\right) d\theta \nonumber \\&\quad =\frac{\pi }{24\alpha }-\frac{\alpha }{24\pi }\pm \frac{\pi }{4\alpha ^{2}}\int _{0}^{\alpha /2}\left( \sin ^{-2}\left( \frac{\pi }{\alpha }\theta \right) -\frac{\alpha ^{2}}{\pi ^{2}\theta ^{2}}\right) d\theta {\mp }\frac{1}{4\pi }\nonumber \\&\qquad \int _{0}^{\alpha /2}\left( \sin ^{-2}\theta -\frac{1}{\theta ^{2}}\right) d\theta \nonumber \\&\quad =\frac{\pi }{24\alpha }-\frac{\alpha }{24\pi }\pm \frac{1}{4\pi }\cot \frac{\alpha }{2}. \end{aligned}$$

Using that, by reflection principle applied to the line $\mathrm {arg}z=\alpha$, we have

7.5 $$\begin{aligned} P^{\Upsilon _{\mathcal {N}\mathcal {D}}^{\alpha }}\left( x,y,t\right)= & {} P^{\Upsilon _{\mathcal {D}}^{2\alpha }}\left( x,y,t\right) +P^{\Upsilon _{\mathcal {D}}^{2\alpha }}\left( x,e^{2i\alpha }{\bar{y}},t\right) ;\end{aligned}$$

7.6 $$\begin{aligned} P^{\Upsilon _{\mathcal {D}\mathcal {N}}^{\alpha }}\left( x,y,t\right)= & {} P^{\Upsilon _{\mathcal {N}}^{2\alpha }}\left( x,y,t\right) -P^{\Upsilon _{\mathcal {N}}^{2\alpha }}\left( x,e^{2i\alpha }{\bar{y}},t\right) , \end{aligned}$$

a similar straightforward but tedious computation yields

7.7 $$\begin{aligned} \int _{0}^{\frac{\alpha }{2}}I_{\mathbb {H}_{\mathcal {N}}}^{\Upsilon _{\mathcal {D}\mathcal {N}}^{\alpha }}(e^{i\theta })d\theta =-\frac{\pi }{48\alpha }-\frac{\alpha }{24\pi }-\frac{1}{4\alpha }+\frac{1}{4\pi }\cot \frac{\alpha }{2}; \end{aligned}$$

7.8 $$\begin{aligned} \int _{0}^{\frac{\alpha }{2}}I_{\mathbb {H}_{\mathcal {D}}}^{\Upsilon _{\mathcal {N}\mathcal {D}}^{\alpha }}(e^{i\theta })d\theta =-\frac{\pi }{48\alpha }-\frac{\alpha }{24\pi }+\frac{1}{4\alpha }-\frac{1}{4\pi }\cot \frac{\alpha }{2}. \end{aligned}$$

Now we are ready to collect the values of $C_{\Upsilon ^{\alpha }}$ for $\alpha \le \pi$: ${\hat{C}}_{\alpha }^{\mathcal {D}\mathcal {D}}$ and ${\hat{C}}_{\alpha }^{\mathcal {N}\mathcal {N}}$ consist of two equal terms given by (7.4), with the cotangent terms canceling out the corresponding terms in (6.7), while ${\hat{C}}_{\alpha }^{\mathcal {D}\mathcal {N}}$ is given by the sum of (7.7) and (7.8) above. If $\alpha >2\pi$, then we need, in addition, to compute the contribution of the third term in (6.5) which is done similarly, and change the integration limits in (7.4), (7.7–7.8) to $\frac{\pi }{2};$ we leave it to the reader to check that the answer is (unsurprisingly) given by the same analytic expression in $\alpha$.

In the case of a puncture, we can compute

$$\begin{aligned} I_{\mathbb {C}}^{\mathbb {C}\setminus \{0\},\varphi _{p}}(1)&=\int _{0}^{\infty }\left( \sum _{k\in \mathbb {Z}}^{\infty }\mathrm {Tr\,}M^{k}\tilde{P}(1,e^{2\pi ik},t)-d\cdot \sum _{k\in \mathbb {Z}}\tilde{P}(1,e^{2\pi ik},t)\right) \frac{dt}{t}\\&=\sum _{k\in \mathbb {Z}\setminus \{0\}}^{\infty }\left( \mathrm {Tr\,}M^{k}-d\right) \frac{1}{\pi (2\pi k)^{2}}=\frac{1}{2\pi }\frac{1}{\pi ^{2}}\sum _{k=1}^{\infty }\left( \Re \mathfrak {e}\,\mathrm {Tr\,}M^{k}-d\right) \frac{1}{k^{2}}. \end{aligned}$$

## Explicit Computation of the Constant *B* for Some Lattices

**Fig. 4:**
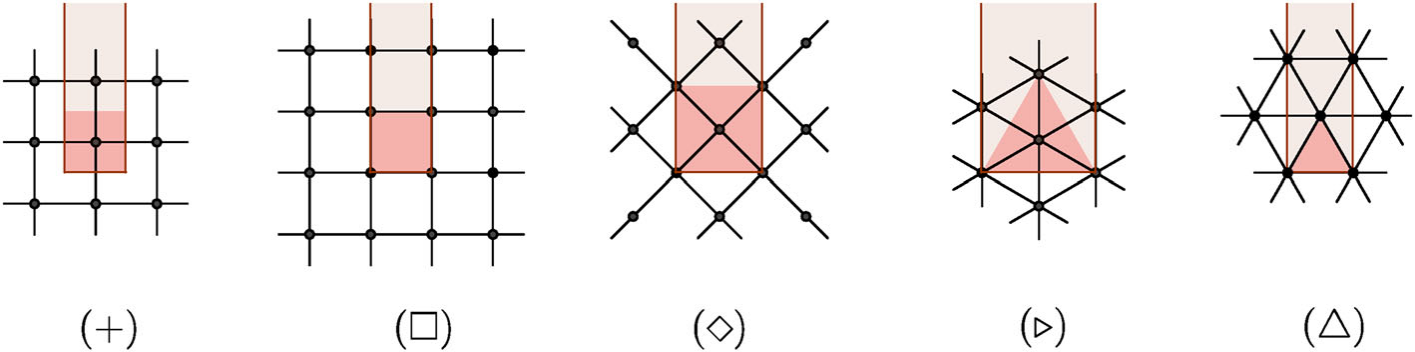
The lattices considered in Sect. 8, their fundamental domains and half-strips $R_{\infty }^{\delta _{0}}$

In this section, we show how the exact values of $B_{\mathcal {N}}=-B_{\mathcal {D}}$ can be computed for several nice lattices, see Figure 4. We assume $d=1,$ since, as remarked before, the constants $A,B_{\mathcal {D}},B_{\mathcal {N}}$ depend linearly on *d*. We note that the values $B_{\mathcal {N}}^{+}$ and $B_{\mathcal {D}}^{\square }$ are related to each other by planar UST duality, see [13, Section 5], therefore, in fact, each of $B_{\mathcal {N}}^{+},B_{\mathcal {N}}^{\square },B_{\mathcal {D}}^{+},B_{\mathcal {D}}^{\square }$ can be deduced from the results in [13]. Similarly, it can be deduced from duality that $B_{\mathcal {N}}^{\Diamond }=B_{\mathcal {D}}^{\Diamond },$ and hence $B_{\mathcal {N}}^{\Diamond }=B_{\mathcal {D}}^{\Diamond }=0.$ Here, we give a direct self-contained computation.

For each of those lattices, the constant ${\hat{B}}_{\mathcal {N}}=-{\hat{B}}_{\mathcal {D}}$ can be expressed in the form

$$\begin{aligned} {\hat{B}}_{\mathcal {N}}=\sum _{x\in R_{\infty }^{\delta _{0}}(0)}I_{\mathbb {C}^{\delta _{0}}}^{\mathbb {H}^{\delta _{0}}}(x)=\sum _{x\in R_{\infty }^{\delta _{0}}(0)}\int _{0}^{\infty }P^{\mathbb {C}^{\delta _{0}}}(x,{\bar{x}},t)\,\frac{dt}{t}=\int _{0}^{\infty }\sum _{y\in S}P^{\mathbb {C}^{\delta _{0}}}(0,y,t)\,\frac{dt}{t}, \end{aligned}$$

where $S:=\{{\bar{x}}-x\mid x\in R_{\infty }^{\delta _{0}}(0)\}$, and we shift the lattice ($+$) so that it has a vertex at the origin. For the lattice $(+)$ (resp. $(\square )$), we have $S=\{v\in \mathbb {C}^{\delta _{0}}:\Re \mathfrak {e}\,v=0,\Im \mathfrak {m}\,v<0\text { odd}\}$ (resp., $S=\{v\in \mathbb {C}^{\delta _{0}}:\Re \mathfrak {e}\,v=0,\Im \mathfrak {m}\,v<0\text { even}\}$). For the lattices $(\Diamond )$, $(\triangleright )$, $(\triangle )$, we have $S=\{v\in \mathbb {C}^{\delta _{0}}:\Re \mathfrak {e}\,v=0,\Im \mathfrak {m}\,v<0\}.$

Denote $(X_{t},Y_{t}):=\gamma _{t}^{\mathbb {C}^{\delta _{0}}}$, started at the origin. Since $P^{\mathbb {C}^{\delta _{0}}}(0,y,t)=P^{\mathbb {C}^{\delta _{0}}}(0,{\bar{y}},t)$, one has

8.1 $$\begin{aligned} 2{\hat{B}}_{\mathcal {N}}^{*}={\left\{ \begin{array}{ll} \int _{0}^{\infty }\mathbb {P}(X_{t}=0\text { and }Y_{t}\text { is odd})\,\frac{dt}{t}, &{} { {*=+,}}\\ \int _{0}^{\infty }\left( \mathbb {P}(X_{t}=0\text { and }Y_{t}\text { is even})-\mathbb {P}(X_{t}=Y_{t}=0)\right) \,\frac{dt}{t}, &{} {{*=\square },}\\ \int _{0}^{\infty }\left( \mathbb {P}(X_{t}=0)-\mathbb {P}(X_{t}=Y_{t}=0)\right) \,\frac{dt}{t}, &{} { {*=\Diamond ,\triangleright ,\triangle }{.}} \end{array}\right. } \end{aligned}$$

Let $\gamma ^{\mathbb {Z}}_{t}$ be the continuous time symmetric random walk on $\mathbb {Z}$ that moves with intensity 1. Note that in the cases $*=+,\square ,\Diamond ,\triangleright ,$ we have $X_{t}{\mathop {=}\limits ^{\mathcal {D}}}\alpha ^{*}\gamma ^{\mathbb {Z}}_{\beta ^{*}t},$ with $\beta ^{+}=\beta ^{\square }=1,$ $\beta ^{\Diamond }=2$, $\beta ^{\triangleright }=\frac{2}{\sqrt{3}}.$ In the cases $*=+$ and $*=\square$, $X_{t}$ and $Y_{t}$ are independent. We compute $\mathbb {P}(\gamma ^{\mathbb {Z}}_{t}\text { is even})=\frac{1}{2}(1+e^{-2t})=:K^{\mathbb {Z}_{2}}(t)$ and $\mathbb {P}(\gamma ^{\mathbb {Z}}_{t}=0)=e^{-t}I_{0}(t)=:K^{\mathbb {Z}}(t)$ where $I_{0}(t)=\frac{1}{\pi }\int _{0}^{\pi }e^{t\cos \phi }\,d\phi$ is the modified Bessel function of the first kind (see [7] or the computation in the end of this section). Recall from Sect. 4 that in all cases, $\int _{0}^{\infty }\left( \mathbb {P}(X_{t}=Y_{t}=0)-e^{-t}\right) \,\frac{dt}{t}=-A$ .

Below we will use the formula

8.2 $$\begin{aligned} \int _{0}^{\infty }\left( e^{-at}I_{0}(t)-e^{-bt}\right) \,\frac{dt}{t}=\log (a-\sqrt{a^{2}-1})+\log 2b\text { for }a\ge 1,b>0, \end{aligned}$$

which follows from the formula for the Laplace transform of the Bessel function, $\int _{0}^{\infty }e^{-at}I_{0}(t)\,dt=1/\sqrt{a^{2}-1},$ $a>1$, by taking the derivative of (8.2) with respect to *a* and matching the behavior as $a\rightarrow \infty .$

With these observations, we are ready to evaluate the right-hand side of (8.1) for $*=+,\square ,\Diamond ,\triangleright$:

$$\begin{aligned} {\hat{B}}_{\mathcal {N}}^{+}= & {} \frac{1}{2}\int _{0}^{\infty }K^{\mathbb {Z}}(t)(1-K^{\mathbb {Z}_{2}}(t))\,\frac{dt}{t}\\= & {} \frac{1}{4}\left[ \int _{0}^{\infty }\left( e^{-t}I_{0}(t)-e^{-t}\right) \,\frac{dt}{t}-\int _{0}^{\infty }\left( e^{-3t}I_{0}(t)-e^{-t}\right) \,\frac{dt}{t}\right] \\= & {} \frac{1}{4}\log (3-\sqrt{8}) =\frac{1}{2}\log (\sqrt{2}-1),\\ {\hat{B}}_{\mathcal {N}}^{\square }= & {} \frac{1}{2}\int _{0}^{\infty }K^{\mathbb {Z}}(t)\left( K^{\mathbb {Z}_{2}}(t)-K^{\mathbb {Z}}(t)\right) \,\frac{dt}{t}\\= & {} \frac{1}{2}\left[ \underbrace{\int _{0}^{\infty }\left( e^{-t}I_{0}(t)-e^{-t}\right) \,\frac{dt}{t}}_{=\log 2}-\underbrace{\int _{0}^{\infty }[K^{\mathbb {Z}}(t)]^{2}-e^{-t}\,\frac{dt}{t}}_{=-A}-\underbrace{\int _{0}^{\infty }K^{\mathbb {Z}}(t)(1-K^{\mathbb {Z}_{2}}(t))\,\frac{dt}{t}}_{=\log (\sqrt{2}-1)}\right] , \\ {\hat{B}}_{\mathcal {N}}^{\Diamond }= & {} \frac{1}{2}\int _{0}^{\infty }\left( K^{\mathbb {Z}}(2t)-\mathbb {P}(X_{t}=Y_{t}=0)\right) \,\frac{dt}{t}\\= & {} \frac{1}{2}\left[ \underbrace{\int _{0}^{\infty }\left( e^{-2t}I_{0}(2t)-e^{-t}\right) \,\frac{dt}{t}}_{=0}-\underbrace{\int _{0}^{\infty }\left( \mathbb {P}(X_{t}=Y_{t}=0)-e^{-t}\right) \,\frac{dt}{t}}_{=-A}\right] \\ {\hat{B}}_{\mathcal {N}}^{\triangleright }= & {} \frac{1}{2}\int _{0}^{\infty }\left( K^{\mathbb {Z}}(2/\sqrt{3}\cdot t)-\mathbb {P}(X_{t}=Y_{t}=0)\right) \,\frac{dt}{t}\\= & {} \frac{1}{2}\left[ \underbrace{\int _{0}^{\infty }\left( e^{-2/\sqrt{3}\cdot t}I_{0}(2/\sqrt{3}\cdot t)-e^{-t}\right) \,\frac{dt}{t}}_{=\log \sqrt{3}}-\underbrace{\int _{0}^{\infty }\left( \mathbb {P}(X_{t}=Y_{t}=0)-e^{-t}\right) \,\frac{dt}{t}}_{=-A}\right] \end{aligned}$$

and plugging into (6.8) gives

$$\begin{aligned} B_{\mathcal {N}}^{+}&=-B_{\mathcal {D}}^{+}=-\frac{1}{2}\log (\sqrt{2}-1);\\ B_{\mathcal {N}}^{\square }&=-B_{\mathcal {D}}^{\square }=\frac{1}{2}(\log (\sqrt{2}-1)-\log 2);\\ B_{\mathcal {N}}^{\Diamond }&=-B_{\mathcal {D}}^{\Diamond }=0\\ B_{\mathcal {N}}^{\triangleright }&=-B_{\mathcal {D}}^{\triangleright }=-\frac{1}{4}\log 3. \end{aligned}$$

In the case $\star =\triangle ,$ $X_{t}$ is equal in law to a scaled copy of a continuous time walk on $\mathbb {Z}$ jumping by $-2,-1,1,2$ with intensities $\frac{\sqrt{3}}{6},\frac{2\sqrt{3}}{6},\frac{2\sqrt{3}}{6},\frac{\sqrt{3}}{6}$ respectively. Its generator is ${\tilde{\Delta }}=f(\Delta _{\mathbb {Z}})$, where $f(x):=\frac{2\sqrt{3}}{3}(x^{2}+3x)$ and $\Delta _{\mathbb {Z}}$ is the generator of $\gamma ^{\mathbb {Z}}_{t}$. When considered on $(\mathbb {Z}/M\mathbb {Z}),$ both $\Delta$ and ${\tilde{\Delta }}$ have eigenfunctions $\psi _{m}(x)=e^{\frac{2\pi imx}{M}}$, and the eigenvalues are $\lambda _{m}=1-\cos \frac{2\pi im}{M}$ and ${\tilde{\lambda }}_{m}=f(\lambda _{m})$, respectively. Thus, for any *M* we explicitly find that $\mathbb {P}(X_{t}\in M\mathbb {Z})=\frac{1}{M}\sum _{m=1}^{M}\exp (-tf(1-\cos \frac{2\pi im}{M}))$ and, passing to the limit as $M\rightarrow \infty$, we have

$$\begin{aligned} \mathbb {P}(X_{t}=0)=\int _{0}^{1}\exp (-tf(1-\cos {2\pi is}))\,ds, \end{aligned}$$

so

$$\begin{aligned} B_{\mathcal {N}}^{\triangle }&=-B_{\mathcal {D}}^{\triangle }=-\frac{1}{2}\int _{0}^{\infty }\left( \mathbb {P}(X_{t}=0)-e^{-t}\right) \,\frac{dt}{t}\\&=-\frac{1}{2}\int _{0}^{1}\int _{0}^{\infty }\left( \exp (-tf(1-\cos {2\pi is}))-\exp (-t)\right) \,\frac{dt}{t}\,ds\\&=\frac{1}{2}\int _{0}^{1}\log f(1-\cos {2\pi is})\,ds \end{aligned}$$

## Proof of Theorem 1.1

To identify the universal constant term in (1.1), we derive an analog of the key formula in the continuum. This is slightly more delicate than in the discrete because the continuous heat kernel is more singular at $t=0$, in particular, there is no *s* for which the integral $\int _{0}^{\infty }P^{\mathbb {C}}(x,x,t)t^{s-1}dt$ converges. Therefore, we perform the analytic continuation in two steps. Starting with (3.4), we write

9.1 $$\begin{aligned} \Gamma (s)\zeta ^{\Omega ,\varphi }(s)&=\int _{1}^{\infty }\left( \Theta ^{\Omega ,\varphi }(t)-k\right) t^{s-1}\,dt+\int _{\Omega }\int _{0}^{1}\left( \mathrm {Tr\,}P^{\Omega ,\varphi }(x,x,t)-d\nonumber \right. \\&\quad \left. \cdot P^{\Omega _{x}}(x,x,t)\right) t^{s-1}\,dtdx +\, d\int _{\Omega }\int _{0}^{1}\left( P^{\Omega _{x}}(x,x,t)-P^{\mathbb {C}}(x,x,t)\right) t^{s-1}\,dxdt\nonumber \\&\quad +d|\Omega |\int _{0}^{1}\frac{1}{2\pi t}t^{s-1}dt-\int _{0}^{1}kt^{s-1}\,dt. \end{aligned}$$

The first two terms converge for all *s* and rest converge when $\Re \mathfrak {e}\,s>1$. The last two terms evaluate to $\frac{d|\Omega |}{2\pi (s-1)}-\frac{k}{s}$. Let’s have a closer look at the third term. To construct its analytic continuation to $s=0$, we first remark that it is, in fact, already analytic for $\Re \mathfrak {e}\,s>\frac{1}{2}.$ Indeed, for $x\in \mathbb {H}$, let $\rho :=\Im \mathfrak {m}\,x$; by Brownian scaling, $P^{\mathbb {H}}(x,x,t)=\rho ^{-2}P^{\mathbb {H}}(i,i,t/\rho ^{2})$ and thus

$$\begin{aligned} \int _{0}^{1}\left| P^{\mathbb {H}}(x,x,t)-P^{\mathbb {C}}(x,x,t)\right| |t^{s-1}|\,dt&=(2\pi )^{-1}\rho ^{2\Re \mathfrak {e}\,s-2}\int _{0}^{\rho ^{-2}}e^{-\frac{2}{t}}|t^{s-2}|\,dt\\&\le {\left\{ \begin{array}{ll} C\rho ^{2\Re \mathfrak {e}\,s-2}, &{} \Re \mathfrak {e}\,s<1;\\ C|\log \rho |, &{} \Re \mathfrak {e}\,s=1,\\ C, &{} \Re \mathfrak {e}\,s>1. \end{array}\right. } \end{aligned}$$

Hence, the integral in the third term in (9.1) over $x:\Omega _{x}\backsimeq \mathbb {H}$ converges absolutely for all *s* with $\Re \mathfrak {e}\,s>\frac{1}{2}.$ For $\Omega _{x}$ a cone, the same scaling argument leads to the same bound with $\rho$ the distance to the tip, thus, the contribution of those *x* converges absolutely for $\Re \mathfrak {e}\,s>0$. Finally, near a corner, we break the integral down as in (6.4) and treat the first three terms as in the cone case and the last two as in the half-plane case.

Now, for $\frac{1}{2}<\Re \mathfrak {e}\,s<1$, we can write

$$\begin{aligned} \int _{0}^{1}\left( P^{\Omega _{x}}(x,x,t)-P^{\mathbb {C}}(x,x,t)\right) t^{s-1}\,dt&=I_{\mathbb {C}}^{\Omega _{x}}(x,s) -\int _{1}^{\infty }P^{\Omega _{x}}(x,x,t)t^{s-1}\,dt\\&\quad -\frac{1}{2\pi (s-1)}, \end{aligned}$$

where $I_{\Lambda _{1}}^{\Lambda _{2}}(x,s):=\int _{0}^{\infty }\left( P^{\Lambda _{2}}(x,x,t)-P^{\Lambda _{1}}(x,x,t)\right) t^{s-1}\,dt,$ cf. the notation in Sect. 6. The last two terms give a contribution that is analytic over $\Re \mathfrak {e}\,s<1,$ hence our task is to analytically continue $\int _{\Omega }I_{\mathbb {C}}^{\Omega _{x}}(x,s).$ We split it into contributions of neighborhoods of cones, boundary segments, corners and punctures, and leverage the fact that for the scaling on each of $\Omega _{x},$ one has $I_{\mathbb {C}}^{\Omega _{x}}(x,s)=a^{2-2s}I_{\mathbb {C}}^{\Omega _{x}}(ax,s)$. For $\Omega _{x}\backsimeq \mathcal {C}^{\alpha }\backsimeq \mathbb {C}/\{z\sim e^{i\alpha }z\},$ we thus get

9.2 $$\begin{aligned} \int _{x\in \mathcal {C}^{\alpha }:|x|<r}I_{\mathbb {C}}^{\mathcal {C}^{\alpha }}(x,s)=I_{\mathbb {C}}^{\mathcal {C}^{\alpha }}(1,s)\cdot \int _{x\in \mathcal {C}^{\alpha }:|x|<r}|x|^{2s-2}=\alpha \cdot I_{\mathbb {C}}^{\mathcal {C}^{\alpha }}(1,s)\cdot \frac{r^{2s}}{2s}, \end{aligned}$$

This is analytic for $\Re \mathfrak {e}\,s\le 1$ when divided by $\Gamma (s)$, which is the only thing we care about; when we eventually evaluate the derivative at 0, we get some value that can be absorbed into the constant $D_{\mathcal {C}^{\alpha }}.$ For the contributions of boundary segments and boundary corners, we split their neighborhoods as in Sects. 6.2 and 6.3. The integral over $R_{r}$ yields

9.3 $$\begin{aligned} \int _{\Im \mathfrak {m}\,x\le r,0\le \Re \mathfrak {e}\,x\le 1}I_{\mathbb {C}}^{\mathbb {H}}(x,s)\,dx=I_{\mathbb {C}}^{\mathbb {H}}(i,s)\cdot \int _{0}^{r}\rho ^{2s-2}d\rho =I_{\mathbb {C}}^{\mathbb {H}}(i,s)\cdot \frac{r^{2s-1}}{2s-1}. \end{aligned}$$

For the other contributions, note that those of $Y_{0,1}$ cancel out as in Sects. 6.2–6.3, and other contributions can be treated as in the cone case, eventually contributing a constant that can be absorbed into $D_{\Upsilon ^{\alpha }};$ same applies to punctures. Dividing (9.1) by $\Gamma (s)$ and differentiating at $s=0$, we get

9.4 $$\begin{aligned} -\log \mathrm {det}^{\star }_{\zeta }\Delta ^{\Omega ,\varphi }&=\int _{1}^{\infty }\left( \Theta ^{\Omega ^{\delta },\varphi }(t)-k\right) \frac{dt}{t}\nonumber \\&\quad +\int _{\Omega }\int _{0}^{1}\left( \mathrm {Tr\,}P^{\Omega ,\varphi }(x,x,t)-d\cdot P^{\Omega _{x}}(x,x,t)\right) \frac{dt}{t}dx\nonumber \\&\quad -d\int _{\Omega }\int _{1}^{\infty }P^{\Omega _{x}}(x,x,t)\frac{dt}{t}dx-k\gamma _{\mathrm {Euler}}-I_{\mathbb {C}}^{\mathbb {H}_{\mathcal {D}}}(i)r^{-1}|\partial _{\mathcal {D}}\Omega |\nonumber \\&\quad -I_{\mathbb {C}}^{\mathbb {H}_{\mathcal {N}}}(i)r^{-1}|\partial _{\mathcal {N}}\Omega | +\sum _{p\in \mathcal {C}^{}\cup \Upsilon \cup \mathcal {P}}\tilde{D}_{p}. \end{aligned}$$

### Remark 9.1

The equation (9.1) also allows one to see that in fact, $C=-2\zeta _{\Omega }(0).$ Since $\frac{1}{\Gamma (s)}\sim s$ near the origin, $\zeta _{\Omega }(0)$ only receives the contributions from those terms in the right-hand side of (9.1) that have a pole at the origin, that is, the third term and $-\frac{k}{s}$. The third term is split into the contributions from near boundary segments, i.e., where $\Omega _{x}=\mathbb {H},$ and the contributions from neighborhoods of the punctures, the cone tips and the corners. The former is evaluated at (9.3) and has no pole at the origin, and the contribution from a cone tip is computed in (9.2), with the residue at $s=0$ matching the coefficient found in (6.2). A similar result holds for boundary corners and punctures.

We are in the position to put everything together and prove Theorem 1.1:

### Proof of Theorem 1.1

We look at the key formula (4.1) term by term. The first three terms converge to the first three terms of (9.4) by Lemma 5.6 and Corollary 6.4. The fourth term can be broken into the contributions of neighborhoods of conical singularities, punctures, and the boundary, whose asymptotics is given by (6.2), (6.9), (6.6), and the constants $C_{p}$ are made explicit in Sect. 7. The fifth term gives the “volume” contribution that is discussed in the end of Sect. 4. $\square$

### Remark 9.2

The underlying triangulation or quadrangulation structure of $\Omega$ was only used in the discretization procedure, but otherwise it plays no role in the proof. While we found no elegant way to state Theorem 1.1 in a more general form that would account for that, we give an example: let $\mathbb {T}_{\omega _{1}^{\delta },\omega _{2}^{\delta }}=\mathbb {C}^{\delta }/(\omega _{1}^{\delta }\mathbb {Z}+\omega _{2}^{\delta }\mathbb {Z})$ be a sequence of discretized tori, whose periods $\omega _{1,2}^{\delta }$ converge as $\delta \rightarrow 0$ to $\omega _{1,2}\in \mathbb {C}\setminus \{0\}$ with $\omega _{2}/\omega _{1}\notin \mathbb {R}$. Then, the asymptotics (1.1) holds, and since there are no boundary or corners/cones, it takes the form

$$\begin{aligned} \log \mathrm {det}^{\star }\Delta ^{\Omega ^{\delta },\varphi }=A\cdot |\mathbb {T}_{\omega _{1}^{\delta },\omega _{2}^{\delta }}|-2\dim \ker \Delta ^{\Omega ^{\delta },\varphi }\cdot \log \delta +\log \mathrm {det}^{\star }_{\zeta }\Delta ^{\Omega ,\varphi }+o(1),\quad \delta \rightarrow 0. \end{aligned}$$

This extends the results in [7, 8, 13, 19]. The above proof applies verbatim; also note that the symmetries of the lattice (other than the double periodicity) are not needed here, as long as the embedding is such that the random walk converges to the Brownian motion.

## Proof of the Lemmas

In preparation for the proof of Lemmas 3.1 and A.4, we prove the following bounds on the transition kernel $P^{{\hat{\Omega }}}(x,y,t)$ of for the process ${\hat{\gamma }}$ defined in Sect. 3, in particular, establishing the existence of this transition kernel.

### Lemma 10.1

The transition kernel $P^{{\hat{\Omega }}}(x,y,t)$ exists, and there is a constant $C>0$ such that if $B_{R}(x_{0})\subset {\hat{\Omega }}$ is isometric to a Euclidean disc, then

10.1 $$\begin{aligned} P^{{\hat{\Omega }}}(x,y,t)&\le \frac{C}{R^{2}}e^{-\frac{R^{2}}{8t}},&\forall x\notin B_{R}(x_{0}),y\in B_{\frac{R}{2}}(x_{0}),t>0; \end{aligned}$$

10.2 $$\begin{aligned} |P^{{\hat{\Omega }}}(x,y,t)-P^{\mathbb {C}}(x,y,t)|&\le \frac{C}{R^{2}}e^{-\frac{R^{2}}{2t}},&\forall x,y\in B_{\frac{R}{2}}(x_{0}),t>0. \end{aligned}$$

### Proof

If ${\check{\gamma }}$ is the Brownian motion in $\mathbb {C}$ started at the origin and $\tau =\min \{t:|{\check{\gamma }}(t)|\ge r\},$ then, by symmetry, $\mathbb {P}(|{\check{\gamma }}_{t}|\ge r|\tau <t)\ge \frac{1}{2}.$ Therefore,

10.3 $$\begin{aligned} \mathbb {P}(\mathrm {diam}\,({\check{\gamma }}_{[0,t]})>2r)\le \mathbb {P}(\tau _{r}<t)\le 2\mathbb {P}(|{\check{\gamma }}_{t}|>r)=2e^{-\frac{r^{2}}{2t}}. \end{aligned}$$

Define the sequence of stopping times $\tau _{0}=0,$ $\tau _{2k+1}=\min \{t>\tau _{2k}:|{\hat{\gamma }}_{t}-x_{0}|=\frac{3}{4}R\},$ $\tau _{2k}=\min \{t>\tau _{2k-1}:|{\hat{\gamma }}_{t}-x_{0}|=R\}.$ If $\phi$ is any non-negative continuous function supported inside $B_{R/2}(x_{0}),$ we have $\mathsf {\mathbb {E}}^{x}\phi ({\hat{\gamma }}_{t})=\sum _{k=1}^{\infty }\mathsf {\mathbb {E}}^{x}[\phi ({\hat{\gamma }}_{t})\mathbb {I}_{t\in [\tau _{2k-1},\tau _{2k}]}].$ Conditionally on $\mathcal {F}({\hat{\gamma }}_{[0,\tau _{2k-1}]})$ and on the event $t\in [\tau _{2k-1},\tau _{2k}],$ the distribution of ${\hat{\gamma }}_{t}$ is that of the (time-shifted) Brownian motion started at a point on $\partial B_{\frac{3R}{4}}(x_{0})$ and conditioned to stay in $B_{R}(x_{0}).$ If $\tilde{P}(x,y,t)$ denotes the heat kernel of this conditioned Brownian motion, then we have, for some $C>0,$

10.4 $$\begin{aligned} \sup _{x\in \partial B_{\frac{3R}{4}}(x_{0}),y\in B_{\frac{R}{2}}(x_{0}),t>0}\tilde{P}(x,y,t)\le \frac{C}{R^{2}}. \end{aligned}$$

Indeed, by Brownian scaling, we may assume $R=1$. Write $P^{\mathbb {D}}(x,y,t)=\sum _{i}e^{-\lambda _{i}t} \psi _{i}(x)\psi _{i}(y)$ and $Q(x,t)=\int _{\mathbb {D}}P^{\mathbb {D}}(x,y,t)dy$, where $\psi _{i}$ and $\lambda _{i}$ are normalized eigenfunctions and eigenvalues of the Laplacian in the unit disc $\mathbb {D}$ with zero boundary conditions. We have $\tilde{P}(x,y,t)=P^{\mathbb {D}}(x,y,t)/Q(x,t),$ and $P^{\mathbb {D}}(x,y,t)\le P^{\mathbb {C}}(x,y,t)=\frac{1}{2\pi t}e^{-\frac{|x-y|^{2}}{2t}}.$ From this, it follows that $\tilde{P}(x,y,t)$ is bounded for small *t* and $\tilde{P}(x,y,t){\mathop {\longrightarrow }\limits ^{t\rightarrow \infty }}\psi _{1}(y)$ for large *t*,  and (10.4) follows. We arrive at

$$\begin{aligned} \mathsf {\mathbb {E}}^{x}\phi ({\hat{\gamma }}_{t})&\le \sum _{k=0}^{\infty }\frac{C}{R^{2}}\left( \int _{B_{\frac{R}{2}}(x_{0})}\phi \right) \mathbb {P}(t\in [\tau _{2k-1},\tau _{2k}])\\&\le \frac{C}{R^{2}}\left( \int _{B_{\frac{R}{2}}(x_{0})}\phi \right) \mathbb {P}(\tau _{1}<t)\le \frac{2C}{R^{2}}e^{-\frac{R^{2}}{8t}}\left( \int _{B_{\frac{R}{2}}(x_{0})}\phi \right) \end{aligned}$$

which proves (10.1). To prove (10.2), let $\tau _{R}=\min \{s:|{\hat{\gamma }}_{s}-x_{0}|=R\}$ and note that the Brownian motions ${\hat{\gamma }},{\check{\gamma }}$ are coupled to coincide up to $\tau ,$ so that

$$\begin{aligned} |\mathsf {\mathbb {E}}^{x}\phi ({\hat{\gamma }}_{t})-\mathsf {\mathbb {E}}^{x}\phi ({\check{\gamma }}_{t})|\le (\mathsf {\mathbb {E}}(\mathsf {\mathbb {E}}^{{\hat{\gamma }}_{\tau }}(\phi ({\hat{\gamma }}_{t})|\tau<t)+\mathsf {\mathbb {E}}^{{\hat{\gamma }}_{\tau }}(\phi ({\check{\gamma }}_{t})|\tau<t))\mathbb {P}(\tau _{R}<t), \end{aligned}$$

and (10.2) follows by (10.1) and (10.3). $\square$

### Proof of Lemma 3.1

We first check that ${\hat{\gamma }}_{t}$ is a $\mathrm {Leb}({\hat{\Omega }})$-symmetric Markov process. For ${\hat{x}}_{0},{\hat{y}}_{0}\in {\hat{\Omega }}$ and small $\varepsilon _{x,y}>0,$ fix a ball $B_{\varepsilon }({\check{x}}_{0})\subset \mathbb {C}$ and an isometry ${\tilde{\sigma }}$ of $B_{\varepsilon _{x}}({\check{x}}_{0})$ to $B_{\varepsilon _{x}}({\hat{x}}_{0}).$ Let ${\check{\gamma }}:[0,\tau ]\rightarrow \mathbb {C}$ be a path with ${\check{\gamma }}(0)\in B_{\varepsilon _{x}}({\check{x}}_{0}).$ If its lift ${\hat{\gamma }}$ ends up in $B_{\varepsilon _{y}}({\hat{y}}_{0})$, we denote by $\sigma _{y}({\check{\gamma }},{\tilde{\sigma }})$ the isometry of $B_{\varepsilon _{y}}({\check{y}}_{0})$ to $B_{\varepsilon _{y}}({\hat{y}}_{0})$ obtained by extending ${\tilde{\sigma }}$ along ${\check{\gamma }}$ (otherwise, $\sigma _{y}({\check{\gamma }},{\tilde{\sigma }})$ is undefined.) We denote by $\mathcal {Y}$ the set of all ${\check{y}}_{0}$ obtained in this way, and by $\Sigma _{y}$ the set of all isometries $\sigma _{y}({\check{\gamma }},{\tilde{\sigma }})$ modulo shifts. If ${\hat{\gamma }}$ ends up in $B_{\varepsilon _{x}}({\hat{x}}_{0}),$ we define $\sigma _{x}({\check{\gamma }},{\tilde{\sigma }}),$ $\mathcal {X}$ and $\Sigma _{x}$ similarly. Note that $\mathcal {X},\mathcal {Y}$ are discrete sets in the plane, and $\Sigma _{x},\Sigma _{y}$ are finite with $|\Sigma _{x}|=|\Sigma _{y}|$.

We now write, for the coupled Brownian motions ${\check{\gamma }},{\hat{\gamma }},$

$$\begin{aligned}&\int _{{\hat{x}}\in B_{\varepsilon _{x}}({\hat{x}}_{0})}\mathbb {P}^{{\hat{x}}}({\hat{\gamma }}_{t}\in B_{\varepsilon _{y}}({\hat{y}}_{0}))\,d{\hat{x}}\\&\quad =\frac{1}{|\Sigma _{x}|}\sum _{\sigma \in \Sigma _{x}}\sum _{\sigma '\in \Sigma _{y}}\sum _{{\check{y}}_{0}\in \mathcal {Y}}\int _{{\check{x}}\in B_{\varepsilon _{x}}({\check{x}}_{0})}\int _{{\check{y}}\in B_{\varepsilon _{y}}({\check{y}}_{0})}\\&\qquad \mathbb {P}^{{\check{x}}}\left( \sigma _{y}({\check{\gamma }}_{[0,t]},\sigma )\sim \sigma '|{\check{\gamma }}_{t}={\check{y}}\right) P^{\mathbb {C}}({\check{x}},{\check{y}},t)\,d{\check{x}}d{\check{y}}, \end{aligned}$$

where $\sim$ stands for equality of isometries modulo shifts. By the reversibility of the planar Brownian notion, denoting by ${\check{\gamma }}^{-1}$ the time-reversal of ${\check{\gamma }},$

$$\begin{aligned} \mathbb {P}^{{\check{x}}}\left( \sigma _{y}({\check{\gamma }}_{[0,t]},\sigma )\sim \sigma '|{\check{\gamma }}_{t}={\check{y}}\right)&=\mathbb {P}^{{\check{x}}}\left( \sigma _{x}({\check{\gamma }}_{[0,t]}^{-1},\sigma ')\sim \sigma |{\check{\gamma }}_{t}={\check{y}}\right) \\&=\mathbb {P}^{{\check{y}}}\left( \sigma _{x}({\check{\gamma }}_{[0,t]},\sigma ')\sim \sigma |{\check{\gamma }}_{t}={\check{x}}\right) . \end{aligned}$$

Also, $P^{\mathbb {C}}({\check{x}},{\check{y}},t)=P^{\mathbb {C}}({\check{y}},{\check{x}},t),$ and if we shift every point in $\mathcal {Y}$ to some fixed point in this set, then the point ${\check{x}}_{0}$ gets shifted to every point in $\mathcal {X}.$ Collecting all these observations together, we end up with

$$\begin{aligned} \int _{{\hat{x}}\in B_{\varepsilon _{x}}({\hat{x}}_{0})}\mathbb {P}^{{\hat{x}}}({\hat{\gamma }}_{t}\in B_{\varepsilon _{y}}({\hat{y}}_{0}))\,d{\hat{x}}=\int _{{\hat{y}}\in B_{\varepsilon _{y}}({\hat{y}}_{0})}\mathbb {P}^{{\hat{y}}}({\hat{\gamma }}_{t}\in B_{\varepsilon _{x}}({\hat{x}}_{0}))\,d{\hat{y}}, \end{aligned}$$

which implies that ${\hat{\gamma }}_{t}$ is $\mathrm {Leb}({\hat{\Omega }})$-symmetric. The transition kernel of the reflected process can be written as ${\hat{P}}(x,y,t)+{\hat{P}}(x,{\overline{y}},t),$ implying symmetry, and killing upon hitting a closed subset preserves the class of symmetric processes.

For the boundedness, the bound (10.1) and the symmetry imply that $P^{{\hat{\Omega }}}(x,y,t)\le CR^{-2}$ if either *x* or *y* are at distance at least *R* from any conical tip of ${\hat{\Omega }}.$ Now, fix a small $R>0$, and let $x\in B_{R}(x_{0}),$ where $x_{0}$ is a conical tip. If $y\notin B_{R}(x_{0}),$ then $P^{{\hat{\Omega }}}(x,y,t)\le CR^{-2}$ by the strong Markov property with respect to exit time from $B_{R}(x_{0}).$ Else, coupling the Brownian motions in ${\hat{\Omega }}$ and in the infinite cone $\mathcal {C^{\alpha }}_{x_{0}}$ up to exiting $B_{R}(x_{0}),$ we similarly obtain

$$\begin{aligned} P^{{\hat{\Omega }}}(x,y,t)\le CR^{-2}+P^{\mathcal {C}_{x_{0}}}(x,y,t). \end{aligned}$$

Coupling to the Brownian motion in $\mathbb {C}$, we have $P^{\mathcal {C}_{x_{0}}}(x,y,t)\le \sum _{{\check{y}}\in \mathcal {Y}}P^{\mathbb {C}}({\check{x}},{\check{y}},t)\le {{\mathrm {const}}}(t,\alpha )$ where $\mathcal {Y}$ is the finite set of endpoints of paths in $\mathbb {C}$ starting at ${\check{x}}$ that lift to a path from *x* to *y*.

For the smoothness, if $B_{R}(x_{0})$ is Euclidean, $y\in B_{\frac{R}{2}}(x_{0})$ and $x\notin B_{R}(x_{0}),$ we can write as in the proof of (10.1):

$$\begin{aligned} P^{{\hat{\Omega }}}(x,y,t)=\sum _{k}\mathsf {\mathbb {E}}\mathsf {\mathbb {E}}\left[ \left. \tilde{P}(\gamma _{\tau _{2k-1}},y,t-\tau _{2k-1})\mathbb {I}_{t\in \tau _{2k-1},\tau _{2k}}\right| \mathcal {F}_{\tau _{2k-1}}\right] , \end{aligned}$$

and note that all the derivatives of $\tilde{P}(x,y,t)$ are uniformly bounded over $x\in \partial B_{\frac{3R}{4}}(x_{0}),$ $y\in B_{\frac{R}{2}}(x_{0}),$ $t>0.$ Since *R* is at our disposal, this proves smoothness for $x\ne y;$ for $x=y$, one can use a similar decomposition, on the event that $t<\tau _{2},$ we can use that the heat kerned in the disc is smooth. $\square$

In order to prove Lemma 5.1, we first invoke the functional CLT in the plane:

### Lemma 10.2

(Coupling the random walk to the Brownian motion). It is possible to multiply the weights $w_{xy}$ by a common factor so that for any $\eta >0$, there exist $C,\epsilon >0$ such that for any $\delta ,T>0$ the random walk $\gamma _{\delta ^{-2}t}^{\delta }$ on $\mathbb {C}^{\delta }$ can be coupled to the Brownian motion $\gamma _{t}$ so that

10.5 $$\begin{aligned} \mathbb {P}\left[ \sup \limits _{t\in [0,T]}|\gamma _{\delta ^{-2}t}^{\delta }-\gamma _{t}|>T^{\frac{1}{4}+\eta }\delta ^{\frac{1}{2}-2\eta }\right] <C\exp (-(T\delta ^{-2})^{\epsilon }) \end{aligned}$$

### Proof

For the random walk $\gamma _{t}^{\delta _{0}}$on $\mathbb {C}^{\delta _{0}}$, we define a sequence of times $t_{0}=0$,

$$\begin{aligned} t_{k+1}:=\min \{t\ge t_{k}+1:\gamma _{t}^{\delta _{0}}\cong \gamma _{0}^{\delta _{0}}\}, \end{aligned}$$

where $\cong$ means equality modulo a shift of $\mathbb {C}^{\delta _{0}}$. Then $t_{k}$ and $\gamma _{t_{k}}^{\delta _{0}}$ have i.i.d. increments with exponentially small tails; because of the symmetries of the lattice, the increments of $\gamma _{t_{k}}^{\delta _{0}}$ have zero mean and a scalar covariance matrix $\Sigma$. Let $\tau =\mathsf {\mathbb {E}}t_{1}.$ Put $n=\left\lfloor 2\tau ^{-1}\delta ^{-2}T\right\rfloor .$ Einmahl’s version of KMT theorem ([14, Theorem 4], plug in $H(t):=\exp (\sqrt{t})$, $x:=\delta _{0}n^{\frac{1}{4}}/3$) provides a coupling of $\gamma _{t_{k}}^{\delta _{0}}$ and a Brownian motion ${\tilde{\gamma }}_{t}$ with covariance matrix $\Sigma$ such that

$$\begin{aligned} \mathbb {P}\left[ \sup \limits _{k\le n}|\gamma _{t_{k}}^{\delta _{0}}-{\tilde{\gamma }}_{k}|>\frac{\delta _{0}}{3}n^{\frac{1}{4}}\right] <K_{1}\cdot n\cdot \exp (-K_{2}n^{\frac{1}{4}}). \end{aligned}$$

with $K_{1,2}$ depending only on $\mathbb {C}^{\delta _{0}}.$ We put $\gamma _{t}={\tilde{\gamma }}_{t/\tau }.$ For $t>0$ we set $k(t):=\max \{k:t_{k}<t\}$, then $t_{k(t)}<t\le t_{k(t)+1}$. We estimate

10.6 $$\begin{aligned} |\gamma _{t}^{\delta _{0}}-\gamma _{t}|\le |\gamma _{t}^{\delta _{0}}-\gamma _{t_{k(t)}}^{\delta _{0}}|+|\gamma _{t_{k(t)}}^{\delta _{0}}-\gamma _{\tau k(t)}|+|\gamma _{\tau k(t)}-\gamma _{t}|. \end{aligned}$$

By Chernoff bound, given $\eta >0$, we have $\mathbb {P}(|t_{k}-\tau k|>n^{\frac{1}{2}+\eta })\le C\exp (-cn^{2\eta })$ for each $k\le n$. Therefore, $\mathbb {P}(\exists k\le n:|t_{k}-\tau k|\ge n^{\frac{1}{2}+\eta })\le Cn\exp (-cn^{2\eta }).$ In particular, $\mathbb {P}(\exists t\le T\delta ^{-2}:k(t)>n)\le Cn\exp (-cn^{2\eta }).$ Also, $\mathbb {P}(\exists k\le n:t_{k+1}-t_{k}\ge n^{\frac{1}{2}+\eta })\le C\exp (-cn^{\eta }).$ Together, this implies that $\mathbb {P}(\exists t\le T\delta ^{-2}:|t-\tau k(t)|\ge 2n^{\frac{1}{2}+\eta })\le Cn\exp (-cn^{\eta }).$ For the Brownian motion $\gamma _{t}$, for each fixed $k\le n$, we have $\mathbb {P}(\exists t:|t-\tau k|\le 2n^{\frac{1}{2}+\eta };|\gamma _{t}-\gamma _{\tau k}|\ge \frac{\delta _{0}}{3}n^{\frac{1}{4}+\eta })\le C\cdot \exp (-cn^{\eta }).$ Summing over *k*,  we conclude that $\mathbb {P}(\exists t\le \delta ^{-2}T:|\gamma _{t}-\gamma _{\tau k(t)}|\ge \frac{\delta _{0}}{3}n^{\frac{1}{4}+\eta })\le C\cdot n\cdot \exp (-cn^{\eta }).$ Also, because of exponential tails of $t_{k}-t_{k-1},$ we have $\mathbb {P}(\exists t\le \delta ^{-2}T:|\gamma _{t}^{\delta _{0}}-\gamma _{t_{k(t)}}^{\delta _{0}}|\ge \frac{\delta _{0}}{3}n^{\frac{1}{4}})\le C\cdot n\cdot \exp (-c(n^{1/4}+n^{2\eta })).$ Combining the estimates of the three terms in (10.6) together, we see that

$$\begin{aligned} \mathbb {P}\left[ \sup \limits _{t\in [0,T\delta ^{-2}]}|\gamma _{t}^{\delta _{0}}-\gamma _{t}|>\delta _{0}n^{\frac{1}{4}+\eta }\right] \le {\hat{C}}\exp (-n^{\epsilon }), \end{aligned}$$

for any $\epsilon <\min (\eta ;\frac{1}{4}).$ Scaling time by $\delta ^{-2}$, the lattice by *N*, and the weights $w_{xy}$ so that $N^{-1}\cdot \gamma _{\delta ^{-2}t}\sim \delta _{0}^{-1}\cdot \gamma _{t}$ is a standard Brownian motion, yields the result. $\square$

### Proof of Lemma 5.1

Let ${\hat{\Omega }}$ (respectively, ${\hat{\Omega }}^{\delta }$) be two copies of $\Omega$ glued along the boundary. The random walk ${\hat{\gamma }}_{[\delta ^{-2}t]}^{\delta }$ in ${\hat{\Omega }}^{\delta }$ (respectively, the Brownian motion ${\hat{\gamma }}_{t}$ in ${\hat{\Omega }}$) can be coupled to a random walk ${\check{\gamma }}_{[\delta ^{-2}t]}^{\delta }$ in $\mathbb {C}^{\delta }$ (respectively, to planar Brownian motion ${\check{\gamma }}_{t}$) by moving in the same way locally; note that the BM in ${\hat{\Omega }}$ never visits conical singularities. By (10.5), ${\check{\gamma }}_{[\delta ^{-2}t]}^{\delta }$ can be coupled to ${\check{\gamma }}_{t}$ in such a way that $\sup _{t\le T}|{\check{\gamma }}_{\delta ^{-2}t}^{\delta }-{\check{\gamma }}_{t}|\rightarrow 0$ as $\delta \rightarrow 0$ almost surely. On the event of probability 1 that ${\hat{\gamma }}_{[0,T]}$ does not visit conical singularities, this implies $\mathrm {dist}({\hat{\gamma }}_{\delta ^{-2}t}^{\delta },{\hat{\gamma }}_{t})\le |{\check{\gamma }}_{\delta ^{-2}t}^{\delta }-{\check{\gamma }}_{t}|$ for all $t\le T$ eventually. Reflecting the random walk and the Brownian motion at the Neumann boundary does not increase distances. If $\tau$ (resp. $\tau ^{\delta }$) is the first time ${\hat{\gamma }}_{t}$ (resp. ${\hat{\gamma }}_{\delta ^{-2}t}^{\delta }$) hits $\partial _{D}\Omega$ (resp. $\partial _{D}\Omega ^{\delta }$), then, almost surely, ${\hat{\gamma }}_{t}$ will have points on both sides of the boundary in each interval $(\tau ,\tau +\varepsilon )$. On that event, almost surely, $\tau ^{\delta }\rightarrow \tau$ and hence ${\hat{\gamma }}_{\delta ^{-2}\tau ^{\delta }}^{\delta }\rightarrow {\hat{\gamma }}_{\tau }$. Therefore, stopping at Dirichlet boundary also does not affect the convergence, and $\sup _{t\le T}\mathrm {dist}(\gamma _{[\delta ^{-2}t]}^{\delta };\gamma _{t})\rightarrow 0$, almost surely. This completes the proof. $\square$

### Proof of Lemma 5.2

It suffices to prove the bound for the walk on $\mathbb {C}^{\delta }$ (by passing first to ${\hat{\Omega }}^{\delta }$ as in the proof of Lemma 5.1 and then assuming by Markov property that *x* is at distance at least $\varepsilon /10$ from conical singularities). If $t>\delta ^{\frac{3}{2}},$ we use Lemma 10.2 and bound $\mathbb {P}(\mathrm {diam}\,\gamma _{[0,t\delta ^{-2}]}^{\delta }>\varepsilon )\le \mathbb {P}(\mathrm {diam}\,\gamma _{[0,t]}>\frac{\varepsilon }{3})+\mathbb {P}(\sup _{s\in [0,t]}|\gamma _{s\delta ^{-2}}^{\delta }-\gamma _{s}|>\frac{\varepsilon }{3}).$ The first term converges to 0 super-polynomially in *t* while the second one bounded from above by $C\cdot \exp (-(t\delta ^{-2})^{\epsilon })\le C\cdot \exp (-t{}^{-\frac{\epsilon }{3}})$ provided that $t\le 1$ and $\delta ^{\frac{1}{2}-2\eta }<\frac{\varepsilon }{3}.$

If $t\le \delta ^{\frac{3}{2}},$ then $\delta ^{-2}t\le t^{\frac{1}{3}}\delta ^{-1}.$ Pick ${\hat{c}}>0$ in such a way that for all $\delta$ small enough, the random walk on $\mathbb {C}^{\delta }$ needs at least $K:=\left\lfloor {\hat{c}}\delta ^{-1}\right\rfloor$ steps to reach diameter $\varepsilon$. The probability of this is bounded by $\mathbb {P}(X\ge K),$ where *X* is a Poisson random variable with mean *MK*, and $M=t^{\frac{1}{3}}\delta ^{-1}\max _{x\in \mathbb {C}^{\delta _{0}}}\{\sum _{y\sim x}w_{xy}\}/K\le c't^{\frac{1}{3}},$ with $c'$ a constant depending on $\mathbb {C}^{\delta _{0}}$ and $\varepsilon .$ If $M<\frac{1}{2},$ then, for any $\alpha >0$, we have by Stirling bound

$$\begin{aligned} \mathbb {P}(X\ge K)\le \sum _{n=K}^{\infty }\frac{(MK)^{n}}{n!}e^{-MK}\le 2\frac{(MK)^{K}}{K!}e^{-MK}\le 2M^{\alpha K}e^{K(1-M+(1-\alpha )\log M)}. \end{aligned}$$

Since $1-M+\log M<0$ for $M<1,$ we can pick $\alpha >0$ such that the exponential is bounded by 1 for all $M<\frac{1}{2},$ i.e., $\mathbb {P}(X\ge K)\le 2(c't^{\frac{1}{3}})^{\alpha \left\lfloor {\hat{c}}\delta ^{-1}\right\rfloor }.$ For $\delta$ small enough, the exponent is at least 20, and so we have $\mathbb {P}(\mathrm {diam}\,\gamma _{[0,t\delta ^{-2}]}^{\delta }>\varepsilon )\le \mathbb {P}(X\ge K)\le 2t^{\frac{20}{6}}$ provided that $t<(c')^{-6}.$ $\square$

For $x\in \Omega ^{\delta }$ and $r>10N^{-1}>0,$ denote $Q(x,r):=[0;r^{2}]\times B(x,r).$ The *parabolic Harnack inequality* (PHI) asserts that there exists a constant $C_{H}$ such that, for any $\delta ,$ any *x*, *r* such that $B(x,r)\cap \partial _{D}\Omega ^{\delta }=\emptyset$, and any *u* positive and satisfying

10.7 $$\begin{aligned} \partial _{t}u=\delta ^{2}\cdot \Delta ^{\Omega ^{\delta }}u \end{aligned}$$

in *Q*(*x*, *r*), one has

10.8 $$\begin{aligned} \inf _{Q_{+}(x,r)}u\ge C_{H}\sup _{Q_{-}(x,r)}u, \end{aligned}$$

where $Q_{-}(x,r)=[\frac{1}{4}r^{2};\frac{1}{2}r^{2}]\times B(x,\frac{r}{2})$ and $Q_{+}(x,r)=[\frac{3}{4}r^{2};r^{2}]\times B(x,\frac{r}{2}).$ In our setting, PHI follows from [9, Theorem 1.7]. Delmotte uses normalized Laplcian in which the random walk jumps at rate one; however since he allows for jumps from a vertex to itself, the two setups are equivalent; note that we rescale the graph distance but don’t rescale time, hence the additional factor of $\delta ^{2}$ in (10.7).

Of the three conditions of [9, Theorem 1.7], the volume doubling condition $DV(C_{1})$ and uniform ellipticity conditions $\Delta (c)$ are obvious in our setting: they state that $|B(x,r)|\le C_{1}|B(x,2r)|$ for any *x*, *r*, and $\min _{y\sim x}w_{yx}\ge cw_{x}$ for any *x*, respectively. This third one, the Poincaré inequality $P(C_{2})$, asserts that

$$\begin{aligned}&\sum _{B(x_{0},r)}w_{x}(f(x)-{\overline{f}}_{B})^{2}\le C_{2}r^{2}\sum _{x\sim y\in B(x_{0},2r)}w_{xy}(f(x)-f(y))^{2},\\&{\overline{f}}_{B}=\frac{1}{\sum _{x\in B(x_{0},r)}w_{x}}\sum _{B(x_{0},r)}w_{x}f(x) \end{aligned}$$

Recall (see e.g. [15]) the classical proof of the Poincaré inequality for a ball *B*: by Cauchy–Schwarz, $(f(x)-f(y))^{2}\le \left( \int _{[xy]}|\nabla f(z)|\,dz\right) ^{2}\le |x-y|\int _{[xy]}|\nabla f(z)|^{2}\,dz,$ from which the Poincaré inequality follows by integrating over $x,y\in B$. This proof extends to the discrete settings of balls in $\mathbb {C}^{\delta },$ simply by replacing integration with summation, in particular, integration over a segment [*xy*] with summation over $H\delta$-neighborhood of [*xy*] for a large enough fixed *H*. In the final step, we sum with weights $w_{x}w_{y};$ note that $w_{xy}$ in the right-hand side can be ignored since they are uniformly bounded away from 0. For balls in an infinite cone or an infinite wedge, it suffices to map the cone or the wedge by a bi-Lipschitz map to the plane or the half-plane and apply the same proof, increasing *H* if necessary. Since we only apply PHI to balls in model surfaces, these cases are all we need. A typical example of a function *u* that PHI is applied to is $P^{\Omega ^{\delta }}(x_{0},x,\delta ^{-2}t).$

We recall the standard argument that PHI implies Hölder regularity of solutions to (10.7), namely, there exist $\theta >0$ and $C_{{\mathrm{H}}{\ddot{\mathrm{o}}}{\mathrm{l}}}>0$ such that any $\delta >0$ and for *Q*(*x*, *r*) as above, one has

10.9 $$\begin{aligned} |u(r^{2},x)-u(r^{2},y)|\le C_{{\mathrm{H}}{\ddot{\mathrm{o}}}{\mathrm{l}}}\cdot \left( \frac{|x-y|}{r}\right) ^{\theta }\cdot {\mathrm {osc}}_{Q(x,r)}u,\quad y\in B(x,r). \end{aligned}$$

To prove (10.9), note that if ${\hat{u}}$ is *u* normalized so that $\inf _{Q(x,r)}{\hat{u}}=0$ and $\sup _{Q(x,r)}{\hat{u}}=1$, and $\sup _{Q_{-}(x,r)}{\hat{u}}\ge \frac{1}{2},$ then

$$\begin{aligned} {\mathrm {osc}}_{Q_{+}(x,r)}{\hat{u}}=\sup _{Q_{+}(x,r)}{\hat{u}}-\inf _{Q_{+}(x,r)}{\hat{u}}\le 1-\frac{C_{H}}{2}. \end{aligned}$$

If $\sup _{Q_{-}(x,r)}{\hat{u}}\le \frac{1}{2},$ then passing to $1-{\hat{u}}$ leads to the same conclusion. Hence, ${\mathrm {osc}}_{Q_{+}(x,r)}u\le c\cdot {\mathrm {osc}}_{Q(x,r)}u,$ with $c=1-\frac{C_{H}}{2}<1.$ Applying the same reasoning to $Q_{+}(x,r)$ and iterating, we conclude that if $|y-x|<\frac{r}{2^{k}}$, then $|u(r^{2},x)-u(r^{2},y)|<c^{k}{\mathrm {osc}}_{Q(x,r)}u$, yielding (10.9).

### Proof of Lemma 5.3

Since turning Dirichlet boundary into Neumann one only increases $P^{\Omega ^{\delta }},$ by passing to ${\hat{\Omega }}^{\delta }$ as in the proof of Lemma 5.1, we may assume that $\partial \Omega ^{\delta }=\emptyset$. Let $M=M_{\varepsilon ,\eta }^{\delta }:=\sup _{t\ge \varepsilon ,\mathrm {dist}(x,y)\ge \eta }P^{\Omega ^{\delta }}(x,y,\delta ^{-2}t),$ and fix $t_{0}\ge \varepsilon$ and $x_{0},y_{0}$ with $\mathrm {dist}(x_{0},y_{0})\ge \eta$ such that $P^{\Omega ^{\delta }}(x_{0},y_{0},\delta ^{-2}t_{0})>\frac{M}{2}.$ Applying PHI to $P^{\Omega ^{\delta }}(x,\cdot ,\cdot )$ and to $Q(y_{0},\sqrt{\varepsilon })$ shifted in time by $t_{0}-\frac{\varepsilon }{4}$, we find that $P^{\Omega ^{\delta }}(x_{0},y,\delta ^{-2}t)\ge C_{H}\frac{M}{2}$ if $\mathrm {dist}(y,y_{0})\le \sqrt{\varepsilon }/2$ and $t\in [t_{0}+\frac{\varepsilon }{2},t_{0}+\frac{3\varepsilon }{4}].$ Since $\sum _{y}P^{\Omega ^{\delta }}(x_{0},y,\delta ^{-2}t)=1,$ this implies, for $\eta =0,$

$$\begin{aligned} C_{H}\frac{M_{\varepsilon ,0}^{\delta }}{2}\cdot \#\{y:|y-y_{0}|<\frac{\sqrt{\varepsilon }}{2}\}\le 1, \end{aligned}$$

that is, $M_{\varepsilon ,0}^{\delta }\le C(\varepsilon )\delta ^{2}.$ If $\eta \ne 0$ is fixed and $\varepsilon <4\eta ^{2}$, then $\mathrm {dist}(y,y_{0})<\frac{\sqrt{\varepsilon }}{2}$ implies $\mathrm {dist}(y,x_{0})\ge \eta /2$. By Lemma 5.2, we have $\sum _{y:\mathrm {dist}(x_{0},y)>\eta /2}P^{\Omega ^{\delta }}(x_{0},y,\delta ^{-2}t)\le C(\eta )t^{3}$, thus

$$\begin{aligned} C_{H}\frac{M_{\varepsilon ,\eta }^{\delta }}{2}\cdot \#\{y:|y-y_{0}|<\frac{\sqrt{\varepsilon }}{2}\}\le C(\eta )t^{3}, \end{aligned}$$

i.e., $M_{\varepsilon ,\eta }^{\delta }\le C'(\eta )\frac{\delta ^{2}}{\varepsilon }t^{3}$. Note that if $M_{\varepsilon ,\eta }^{\delta }>M_{2\varepsilon ,\eta }^{\delta },$ then we could take $t_{0}\le 2\varepsilon$ and thus $t\le \frac{11}{4}\varepsilon$, in which case the last inequality becomes $M_{\varepsilon ,\eta }^{\delta }\le C''(\eta )\delta ^{2}\varepsilon ^{2}.$ Hence, $M_{0,\eta }^{\delta }=M_{\varepsilon _{0}(\eta ),\eta }^{\delta }\le M_{\varepsilon _{0}(\eta ),0}^{\delta }$ for some $\varepsilon _{0}(\eta )>0$. $\square$

### Proof of Lemma 5.4

Since $||P^{\Omega ^{\delta },\varphi }(x,y,\tau )||\le P^{\Omega ^{\delta }}(x,y,\tau )$, we have, by Lemma 5.3,

$$\begin{aligned} {\mathrm {osc}}_{Q}(P^{\Omega ^{\delta },\varphi }(x,\cdot ,\cdot ))\le C\delta ^{2}, \end{aligned}$$

where $Q:=[t-\frac{\eta ^{2}}{4},t]\times B(x,\frac{\eta }{2}).$ Since the matrix components of $P^{\Omega ,\varphi }(x,\cdot ,\delta ^{-2}t)$ satisfy (10.7), the result now follows directly from (10.9). $\square$

### Proof of Lemma 5.5

The proof proceeds case by case.

*Case 1.*: Suppose that $\partial _{\mathcal {D}}\Omega \ne \emptyset$, thus $k=0.$ Since $||P^{\Omega ^{\delta },\varphi }(x,y,t)||\le |P^{\Omega ^{\delta }}(x,y,t)|,$ it suffices to prove the result for the trivial line bundle. The probability that by time 1, the Brownian motion $\gamma _{t}$ started at *x* has hit the Dirichlet boundary is a positive continuous function on $\Omega ,$ hence it is bounded from below, say by $2\eta .$ Hence, for $\delta$ small enough, the probability that the random walk $\gamma _{\delta ^{-2}t}^{\delta }$ hits the Dirichlet boundary before $t=1$ is bounded below by $\eta$, independently of the starting point. By Markov property, this implies that the probability that it does not hit $\partial _{\mathcal {D}}\Omega ^{\delta }$ by time *t* is bounded above by $(1-\eta )^{\left\lfloor t\right\rfloor }$, i.e.,
  $$\begin{aligned} \sum _{y\in \Omega ^{\delta }}P^{\Omega ^{\delta }}(x,y,\delta ^{-2}t)\le (1-\eta )^{\left\lfloor t\right\rfloor }. \end{aligned}$$
  Using PHI as in the proof of Lemma 5.3, we see that this implies $P^{\Omega ^{\delta }}(x,y,\delta ^{-2}t)<C\delta ^{2}(1-\eta )^{\left\lfloor t\right\rfloor }$ for any *x*, *y*. Summing this bound over $x=y\in \Omega ^{\delta }$ yields the desired result.
*Case 2.*: Suppose that $\Omega$ has no Dirichlet boundary and $\varphi$ is the trivial line bundle, thus $k=1$. We claim that the heat kernel $P^{\Omega ^{\delta }}(x,y,\delta ^{-2}t)$ at time $t=1$ is uniformly contracting in the total variation distance, i.e., there exists $\eta >0$ such that for any $x_{1},x_{2}$, and any $\delta$ small enough, one has
  10.10 $$\begin{aligned} \frac{1}{2}\sum _{y}\left| P^{\Omega ^{\delta }}(x_{1},y,\delta ^{-2})-P^{\Omega ^{\delta }}(x_{2},y,\delta ^{-2})\right| \le 1-\eta . \end{aligned}$$
  Indeed, for any test function $\psi >0$, by compactness, we have for the *continuous* heat kernel,
  $$\begin{aligned} \inf _{x}\int _{\Omega }P^{\Omega }\left( x,y,\frac{1}{2}\right) \psi (y)dy=:2c_{\psi }>0. \end{aligned}$$
  since the expression under infimum is positive and continuous in *x*. Hence,
  $$\begin{aligned} \inf _{x}\sum _{y\in \Omega ^{\delta }}P^{\Omega ^{\delta }}\left( x,y,\delta ^{-2}/2\right) \psi (y)>c_{\psi }. \end{aligned}$$
  for $\delta$ small enough. If $\mathrm {supp}\,\psi \subset B(y_{0},r)$, say with with $r=1$, this implies that
  $$\begin{aligned} \inf _{x}\sup _{y\in B(y_{0},r)}P^{\Omega ^{\delta }}(x,y,\delta ^{-2}/2)\ge c'_{\psi }\delta ^{2}, \end{aligned}$$
  and hence, by PHI, $\inf _{x}\inf _{y\in B(y_{0},r)}P^{\Omega ^{\delta }}(x,y,\delta ^{-2})\ge C_{H}c'_{\psi }\delta ^{2}.$ This gives the desired improvement on the trivial bound of 1 on the LHS of (10.10). Now, iterating (10.10), we see that $\frac{1}{2}\sum _{y}\left| P^{\Omega ^{\delta }}(x_{1},y,\delta ^{-2}t)-P^{\Omega ^{\delta }}(x_{2},y,\delta ^{-2}t)\right| \le (1-\eta )^{\left\lfloor t\right\rfloor }$, hence
  $$\begin{aligned} \sum _{x}P^{\Omega ^{\delta }}(x,x,\delta ^{-2}t)-k&=\sum _{x}P^{\Omega ^{\delta }}(x,x,\delta ^{-2}t)-\frac{1}{|\Omega ^{\delta }|}\left( \sum _{x,y}P^{\Omega ^{\delta }}(y,x,\delta ^{-2}t)\right) \\&=\frac{1}{|\Omega ^{\delta }|}\sum _{x,y}\left( P^{\Omega ^{\delta }}(x,x,\delta ^{-2}t)- P^{\Omega ^{\delta }}(y,x,\delta ^{-2}t)\right) \\&\le 2\cdot (1-\eta )^{\left\lfloor t\right\rfloor } \end{aligned}$$
  for all $\delta$ small enough, independently of *t*, as required.
*Case 3.*: Suppose that $\Omega$ has no Dirichlet boundary and $k=0$. Pick any $x_{0}\in \Omega ^{\delta }$, and (non-contractible) loops $\beta _{(1)},\dots \beta _{(n)}$ rooted at $x_{0}$ such that $\{\varphi (\gamma _{j})\}_{j=0}^{n}$ do not have a common eigenvector of eigenvalue 1; if such loops did not exist, then the translations of the common eigenvector would form a covariant constant. Pick a a small *r* such that $B(x_{0},r)$ is contractible, and, for $x,y\in B(x_{0},r),$ denote, for $i=0,1,\dots ,n$, $P_{(i)}^{\Omega ^{\delta }}(x,y,t):=\mathbb {P}^{x}([\gamma _{[0,t]}^{\delta }]=[\beta _{(i)}],\gamma _{t}^{\delta }=y)$, where $\beta _{(0)}$ is a contractible loop, and we identify the points of $B(x_{0},r)$ in order to compute the homotopy type of a non-closed path. There exists a constant $c>0$ such that $c\delta ^{2}\le P_{(i)}^{\Omega ^{\delta }}(x,y,\delta ^{-2})\le c^{-1}\delta ^{2}$ for each *i* and each $x,y\in B(x_{0},r)$ and all $\delta$ small enough; the upper bound follows from Lemma 5.3 and the lower one is done exactly as in Case 2. We can write
  10.11 $$\begin{aligned} \Vert P^{\Omega ^{\delta },\varphi }(x,y,\delta ^{-2}t)\Vert&=\left\| \mathsf {\mathbb {E}}^{x}\varphi (\gamma _{[0,\delta ^{-2}t]}^{\delta })\mathbb {I}_{\mathbb {I}_{\gamma _{t}^{\delta }=y}}\right\| \nonumber \\&\le \left\| \sum _{i=0}^{n}\varphi (\beta _{(i)})\cdot P_{(i)}^{\Omega ^{\delta }}(x,y,\delta ^{-2})\right\| \nonumber \\&\quad +\, \mathbb {P}^{x}(\forall i,[\gamma _{[0,t]}^{\delta }]\ne [\beta _{(i)}],\gamma _{t}^{\delta }=y) \end{aligned}$$
  where in the last term, we used that $||\varphi (\gamma _{[0,\delta ^{-2}t]}^{\delta })||\le 1$ as $\varphi$ is unitary. We claim that
  10.12 $$\begin{aligned} \sup _{c\le p_{i}\le c^{-1},\left\| v\right\| =1}\frac{\left\| \sum _{i=0}^{n}p_{i}\varphi (\beta _{(i)})v\right\| }{\left( \sum _{i=0}^{n}p_{i}\right) }=:1-\eta <1. \end{aligned}$$
  Indeed, the fraction is strictly smaller than 1 unless all $\varphi (\beta _{(i)})v$ are non-negative multiples of each other, but since $\varphi (\beta _{0})=\mathrm {Id},$ this would mean that *v* is a common eigenvector of eigenvalue 1. Applying (10.12) to (10.11), we get, for any $x\in B(x_{0},r)$ and all $\delta$ small enough,
  $$\begin{aligned} \sum _{y\in \Omega ^{\delta }}\left\| P^{\Omega ^{\delta },\varphi }(x,y,\delta ^{-2})\right\| \le (1-\eta )\mathbb {P}(E)+P(E^{c})\le 1-\eta ' \end{aligned}$$
  where $E=\{\gamma _{\delta ^{-2}}^{\delta }\in B(x_{0},r)\text { and }\exists i:[\gamma _{[0,\delta ^{-2}]}^{\delta }]=[\beta _{(i)}]\},$ and we have used that $\mathbb {P}(E)\ge \sum _{y\in B(x_{0},r)}P_{(0)}^{\Omega ^{\delta }}(x,y,\delta ^{-2})$ is uniformly bounded from below. Since the bounds obtained, in fact, did not depend on $x_{0},$ the proof is now completed as in Case 1.
*Case 4.*: Suppose that $\Omega$ has no Dirichlet boundary. Let $\varphi _{0}$ be the trivial sub-bundle of the maximal dimension of $\varphi$, which is a direct sum of trivial line bundles. Since $\varphi$ is unitary, we have $\varphi =\varphi _{0}\oplus \varphi _{0}^{\perp }$ for $\varphi _{0}^{\perp }$ the (point-wise) orthogonal complement to $\varphi _{0}$; moreover, $\varphi _{0}^{\perp }$ has no trivial line sub-bundles. Applying Case 2 to $\varphi _{0}$ and Case 3 to $\varphi _{1}$ concludes the proof. $\square$

### Proof of Lemma 6.1

It suffices to consider the case when $\Lambda$ is a cone (or, in particular, a plane). Indeed, $\mathbb {P}^{\mathbb {H}^{\delta }}(x,y,t)=\mathbb {P}^{\mathbb {C}^{\delta }}(x,y,t)\pm \mathbb {P}^{\mathbb {C}^{\delta }}(x,{\bar{y}},t),$ with ${\bar{y}}$ denoting the reflection with respect to the boundary and the sign being $+$ for Neumann boundary condition and − for Dirichlet one. Similarly, for a corner $\Upsilon ^{\alpha }$ with Dirichlet or Neumann boundary conditions, one has $\mathbb {P}^{\Upsilon ^{\alpha ,\delta }}(x,y,t)=\mathbb {P}^{\mathcal {C}^{2\alpha ,\delta }}(x,y,t)\pm \mathbb {P}^{\mathcal {C}^{2\alpha ,\delta }}(x,{\bar{y}},t),$ where the cone $\mathcal {C}^{2\alpha ,\delta }$ is obtained by gluing two copies of $\Upsilon ^{\alpha ,\delta }$ along the boundary. Finally, $\mathbb {P}^{\Upsilon _{\mathcal {D}\mathcal {N}}^{\alpha ,\delta }}(x,y,t)=\mathbb {P}^{\Upsilon _{\mathcal {D}}^{2\alpha ,\delta }}(x,y,t)+\mathbb {P}^{\Upsilon _{\mathcal {D}}^{2\alpha ,\delta }}(x,{\bar{y}},t),$ where $\Upsilon _{\mathcal {D}}^{2\alpha ,\delta }$ is obtained by gluing two copies of $\Upsilon _{\mathcal {D}\mathcal {N}}^{\alpha ,\delta }$ along the Neumann boundary.

The proof below is for the case when there is no vertex at the tip of the cone; we explain the necessary modifications for that case in Remark 10.3. We begin by comparing the probabilities $\mathbb {P}(\gamma _{\delta ^{-2}t}^{\delta }\in B(y,\varepsilon ))$ and $\mathbb {P}(\gamma _{t}\in B(y,\varepsilon ))$ (hereinafter $\mathbb {P}=\mathbb {P}^{x}$) for a mesoscopic scale $\delta \ll \varepsilon (\delta )\ll 1$ to be specified later and $t\le T:=\delta ^{-\frac{1}{10}}$; the goal is to show that they agree up to $O(\max \{t^{-1},1\}\varepsilon ^{2}\delta ^{\rho }$) for some $\rho >0$. We couple $\gamma _{\delta ^{-2}t}^{\delta }$ and $\gamma _{t}$ to the planar random walk and the Brownian motion ${\hat{\gamma }}_{[\delta ^{-2}t]}^{\delta },{\hat{\gamma }}_{t}$ by the same local moves, and assume that ${\hat{\gamma }}_{\delta ^{-2}t}^{\delta },{\hat{\gamma }}_{t}$ are coupled as in Lemma 10.5, say, with $\eta =\frac{1}{10}.$ Pick a positive $\nu <\frac{1}{2}-2\eta -\frac{1}{10}(\frac{1}{4}+\eta )$, so that $T^{\frac{1}{4}+\eta }\delta ^{\frac{1}{2}-\eta }\ll \delta ^{\nu }.$ We put $\mathcal {D}=\{\sup _{t\in [0,T]}|{\hat{\gamma }}_{\delta ^{-2}t}^{\delta }-{\hat{\gamma }}_{t}|>\delta ^{\nu }\}$, $\mathcal {T}=\{\inf _{t\in (0,T)}(\mathrm {dist}(\gamma _{t},0))<2\delta ^{\nu }\},$ where 0 is the tip of the cone, and $\mathcal {B}:=\{\varepsilon (\delta )-\delta ^{\nu }\le \mathrm {dist}(\gamma _{t};y)\le \varepsilon (\delta )+\delta ^{\nu }\}.$ On the event $\mathcal {D}^{c}\cap \mathcal {T}^{c}$, we have $\sup _{t\in (0,T)}|\gamma _{\delta ^{-2}t}^{\delta }-\gamma _{t}|\le \delta ^{\nu }$, therefore, on $\mathcal {D}^{c}\cap \mathcal {T}^{c}\cap \mathcal {B}^{c}$, either $\gamma _{\delta ^{-2}t}^{\delta },\gamma _{t}\in B(y,\varepsilon )$ or $\gamma _{\delta ^{-2}t}^{\delta },\gamma _{t}\notin B(y,\varepsilon )$ simultaneously. This implies

10.13 $$\begin{aligned}&\left| \mathbb {P}(\gamma _{\delta ^{-2}t}^{\delta }\in B(y,\varepsilon ))-\mathbb {P}(\gamma _{t}\in B(y,\varepsilon ))\right| \le \mathbb {P}(\mathcal {D})+\mathbb {P}(\mathcal {B})\nonumber \\&\quad +\left| \mathbb {P}(\gamma _{\delta ^{-2}t}^{\delta }\in B(y,\varepsilon ),\mathcal {D}^{c},\mathcal {T})-\mathbb {P}(\gamma _{t}\in B(y,\varepsilon ),\mathcal {D}^{c},\mathcal {T})\right| . \end{aligned}$$

We have $\mathbb {P}(\mathcal {D})\le C\cdot \delta ^{10}$ and $\mathbb {P}(\mathcal {B})\le C\varepsilon \delta ^{\nu }\cdot t^{-1}$ provided that $\varepsilon \gg \delta ^{\nu },$ thus it remains to estimate the last term. Let $\sigma$ denote the rotation of the cone around its tip by $\pi /3$ (we assume here $\Omega$ is triangulated, the other case is completely similar), and let ${\hat{\sigma }}$ be the rotation, by the same angle, of the plane obtained as a quotient the universal cover of the cone punctured at its tip; thus ${\hat{\sigma }}^{6}=\mathrm {Id}$ and $\sigma ^{\frac{3\alpha }{\pi }}=\mathrm {Id}.$ We have $\gamma _{\delta ^{-2}t}^{\delta }\in \cup _{k}\sigma ^{k}\left( B(y,\varepsilon )\right)$ if and only if ${\hat{\gamma }}_{\delta ^{-2}t}^{\delta }\in \cup _{k}{\hat{\sigma }}^{k}\left( B(y,\varepsilon )\right) ,$ and thus, as above,

10.14 $$\begin{aligned}&\left| \mathbb {P}(\gamma _{\delta ^{-2}t}^{\delta }\in \cup _{k}\sigma ^{k}(B(y,\varepsilon ),\mathcal {D}^{c},\mathcal {T})-\mathbb {P}(\gamma _{t}\in \cup _{k}\sigma ^{k}(B(y,\varepsilon )),\mathcal {D}^{c},\mathcal {T})\right| \nonumber \\&\quad \le \left| \mathbb {P}({\hat{\gamma }}_{\delta ^{-2}t}^{\delta }\in \cup _{k}{\hat{\sigma }}^{k}(B(y,\varepsilon ),\mathcal {D}^{c},\mathcal {T})-\mathbb {P}({\hat{\gamma }}_{t}\in \cup _{k}{\hat{\sigma }}^{k}(B(y,\varepsilon )),\mathcal {D}^{c},\mathcal {T})\right| \nonumber \\&\quad \le {{\mathbb {P}}({\mathcal {B}\cap \mathcal {T}}){\le }}C\varepsilon \delta ^{\nu }t^{-1} \end{aligned}$$

Now let $\tau :=\min \{t:\mathrm {dist}(\gamma _{t},0))<2\cdot \delta ^{\nu }\}.$ On $\mathcal {T}$, to estimate the difference between probabilities to arrive to $B(y,\varepsilon )$ and to $\sigma (B(y,\varepsilon ))$, we use strong Markov property with respect to $\tau .$ We have

$$\begin{aligned}&\left| \mathbb {P}^{\gamma _{\delta ^{-2}\tau }^{\delta }}\left[ \gamma _{\delta ^{-2}(t-\tau )}^{\delta }\in B(y,\varepsilon )\right] -\mathbb {P}^{\gamma _{\delta ^{-2}\tau }^{\delta }}\left[ \gamma _{\delta ^{-2}(t-\tau )}^{\delta }\in \sigma (B(y,\varepsilon ))\right] \right| \\&\quad =\left| \mathbb {P}^{\gamma _{\delta ^{-2}\tau }^{\delta }}\left[ \gamma _{\delta ^{-2}(t-\tau )}^{\delta }\in B(y,\varepsilon )\right] -\mathbb {P}^{\sigma ^{-1}(\gamma _{\delta ^{-2}\tau }^{\delta })}\left[ \gamma _{\delta ^{-2}(t-\tau )}^{\delta }\in B(y,\varepsilon )\right] \right| \\&\quad \le \sup _{x,z\in B(0,3\delta ^{\nu }),\,t\le T}\left| \mathbb {P}^{x}(\gamma _{\delta ^{-2}t}^{\delta }\in B(y,\varepsilon ))-\mathbb {P}^{z}(\gamma _{\delta ^{-2}t}^{\delta }\in B(y,\varepsilon ))\right| . \end{aligned}$$

We estimate the expression in the supremum separately for $t>10\delta ^{\nu }>(3\delta ^{\nu })^{2}$ and for $t<10\delta ^{\nu }.$ In the first case, we use Hölder continuity (10.9) with $r=3\delta ^{\frac{\nu }{2}}$, time shifted by $t-r^{2}>0$, and bounding the oscillation by 1; this gives the bound of $\le C\cdot \delta {}^{\theta \frac{\nu }{2}}.$ In the second case, we use Lemma 5.2 to get the bound of $\le C\delta ^{3\nu }\ll \delta ^{\theta \frac{\nu }{2}}.$ We infer that

$$\begin{aligned} \left| \frac{\pi }{3\alpha }\mathbb {P}(\gamma _{\delta ^{-2}T}^{\delta }\in \cup _{k}\sigma ^{k}(B(y,\varepsilon )),\mathcal {D}^{c},\mathcal {T})-\mathbb {P}(\gamma _{\delta ^{-2}T}^{\delta }\in B(y,\varepsilon (\delta )),\mathcal {D}^{c},\mathcal {T})\right| \le C\cdot \delta {}^{\frac{\theta \nu }{2}}. \end{aligned}$$

A similar estimate holds for the continuous heat kernel. Therefore, we finally get

10.15 $$\begin{aligned}&\left| \mathbb {P}(\gamma _{\delta ^{-2}T}^{\delta }\in B(y,\varepsilon ))-\mathbb {P}(\gamma _{T}\in B(y,\varepsilon ))\right| \nonumber \\&\quad \le C\delta {}^{10}+C\cdot \delta {}^{\frac{\theta \nu }{2}}+C\cdot \varepsilon \delta ^{\nu }\cdot t^{-1}\le C\varepsilon ^{2}\delta ^{\rho }\cdot \max \{t^{-1},1\}. \end{aligned}$$

with $\rho >0$ if we choose $\varepsilon (\delta ):=\delta ^{\mu }$ with $\mu$ small enough.

Now, for $\kappa >0,$ as in the proof of Lemma 5.3, we can bound

$$\begin{aligned} \sup _{t\ge \frac{\delta ^{2\kappa }}{2},z\in B(y,\varepsilon )}\mathbb {P}(\gamma _{\delta ^{-2}t}^{\delta }=z)\le C\delta ^{2-2\kappa }. \end{aligned}$$

Using this to bound the oscillation in the Hölder bound (10.9), we get, for $t\ge \delta ^{2\kappa },$

$$\begin{aligned}&\left| \frac{1}{|B(y,\varepsilon )|}\mathbb {P}(\gamma _{\delta ^{-2}t}^{\delta }\in B(y,\varepsilon ))-\mathbb {P}(\gamma _{\delta ^{-2}t}^{\delta }=y)\right| \\&\quad \le \left( \frac{\varepsilon }{\delta ^{\kappa }}\right) ^{\theta }\cdot \delta ^{2-2\kappa }=\delta ^{\mu \theta +2-(2+\theta )\kappa }=:\delta ^{2+q}, \end{aligned}$$

provided that $\kappa$ is small enough (in which case also $\varepsilon \ll \delta ^{\nu }$ so that the Hölder bound applies). A similar estimate holds for the continuous heat kernel. Combining this with (10.15) gives the claim. $\square$

### Remark 10.3

If there is a vertex at the tip of the cone, then the coupling of $\gamma _{\delta ^{-2}t}^{\delta }$ and ${\hat{\gamma }}_{\delta ^{-2}t}^{\delta }$ fails after the moment the former hits the tip, in particular, the distributions of the first time they leave the tip will be different. However, forcing ${\hat{\gamma }}_{\delta ^{-2}t}^{\delta }$ to leave the tip simultaneously with $\gamma _{\delta ^{-2}t}^{\delta }$ yields a coupling of $\gamma _{\delta ^{-2}t}^{\delta }$ to ${\hat{\gamma }}_{\delta ^{-2}t+\tau }^{\delta }$ such that still $\gamma _{\delta ^{-2}t}^{\delta }\in \cup _{k}\sigma ^{k}\left( B(y,\varepsilon )\right)$ if and only if ${\hat{\gamma }}_{\delta ^{-2}t+\tau }^{\delta }\in \cup _{k}{\hat{\sigma }}^{k}\left( B(y,\varepsilon )\right) .$ Here $\tau$ a random variable given by a sum of *N* i. i. d. contributions, where *N* is the number of visits to the tip. The expectation of *N*, and hence that of $\tau$, is $O(\log (\delta ^{-2}t)),$ and therefore it will introduce a negligible error into the above computations.

### Proof of Lemma 6.2

As explained in in the proof of Lemma 6.1, it suffices to consider the case of a cone. Moreover, since, in the notation of that proof, $\gamma _{\delta ^{-2}t}^{\delta }\in \cup _{k}\{\sigma ^{k}(y)\}$ if and only if ${\hat{\gamma }}_{\delta ^{-2}t}^{\delta }\in \cup _{k}\{{\hat{\sigma }}^{k}(y)\},$ it is in fact sufficient to consider the case of a plane, where it is immediate from the local Central limit theorem. $\square$

## A. Appendix: The Domain of the Dirichlet Form $\mathcal {E}^{\Omega ,\varphi }$

For a unitary vector bundle $\varphi$, the point-wise scalar product $\langle \cdot ;\cdot \rangle$ induces the scalar product on the set $L^{2,\varphi }(\Omega )$ be of $L^{2}$ sections of $\varphi$, given by $\int _{\Omega }\langle f(x);f(x)\rangle dx,$ and also on gradients of nice enough sections, using $\langle \nabla ^{\varphi }f(x);\nabla ^{\varphi }f(x)\rangle =\langle \partial _{\nu }\tilde{f}(x);\partial _{\nu }\tilde{f}(x)\rangle +\langle \partial _{\eta }\tilde{f}(x);\partial _{\eta }\tilde{f}(x)\rangle ,$ where $(\nu ,\eta )$ are isometric local coordinates, and we identify *f* with a function $\tilde{f}$ using a local trivialization of $\varphi$. We thus define $H^{1,\varphi }(\Omega ):=\{f\in L^{2,\varphi }(\Omega ):\nabla ^{\varphi }f\in L^{2}\},$ equipped with the scalar product $\langle f;f\rangle +\langle \nabla ^{\varphi }f;\nabla ^{\varphi }f\rangle .$ Put $H_{\partial }^{1,\varphi }(\Omega )=\{f\in H^{1,\varphi }(\Omega ):\,f\equiv 0\text { on }\partial _{\mathcal {D}}\Omega \};$ the vanishing on the boundary can be understood in the sense of the trace operator in the theory of Sobolev spaces, or, more elementarily, by requiring that the extension of *f* by 0 across the boundary has a finite $H^{1}$ norm. It is well known that for piece-wise smooth boundaries, $H_{\partial }^{1,\varphi }(\Omega )$ is the closure in $H^{1,\varphi }(\Omega )$ of the set of $f\in H^{1,\varphi }(\Omega )$ supported away from $\partial _{\mathcal {D}}\Omega$ (cf. [45, before Theorem 4.11] or [36].) The next lemma describes explicitly the Dirichlet form $\mathcal {E}^{\Omega ,\varphi }(f,f)=\lim _{t\searrow 0}t^{-1}\langle (1-P_{t}^{\Omega ,\varphi })f;f\rangle ,$ defined in Sect. 3.

### Lemma A.4

*We have* $\mathcal {D}(\mathcal {E}^{\Omega ,\varphi })=H_{\partial }^{1,\varphi }(\Omega )$ *and* $\mathcal {E}^{\Omega ,\varphi }(f,f)=\langle \nabla ^{\varphi }f,\nabla ^{\varphi }f\rangle$ *for any* $f\in \mathcal {D}(\mathcal {E}^{\Omega ,\varphi }).$

In other words, the generator $\Delta ^{\Omega ,\varphi }$ is the *Friedrichs* *extension* of the Laplacian acting on smooth sections of $\varphi$ compactly supported away from $\partial _{\mathcal {D}}\Omega$, corners, conical singularities and punctures. Before giving the a proof, we remark that this also follows from the general theory. By [20, Theorem 7.2.2], we can *define* $\gamma _{t}$ as the unique diffusion associated to the Dirichlet form $\mathcal {E}_{BM}(f,f)=\langle \nabla f;\nabla f\rangle$ with the domain $\mathcal {D}(\mathcal {E}_{BM})=H_{\partial }^{1}(\Omega ).$ Moreover, if $\Lambda \subset \Omega$ is isometric to a domain in the plane, then, up to hitting $\partial \Lambda \setminus \partial _{\mathcal {N}}\Omega$, the distribution of $\gamma _{t}$ coincides with that of the Brownian motion in $\Lambda$ reflected at $\partial _{\mathcal {N}}\Omega$ [20, Theorem 4.4.2 and Example 4.5.3]. Therefore, this construction of $\gamma _{t}$ coincides with the one given above. This proves Lemma A.4 for trivial line bundle; for the general case, observe that the multiplication by smooth functions preserves both $\mathcal {D}(\mathcal {E}^{\Omega ,\varphi })$ and $H_{\partial }^{1,\varphi }(\Omega ),$ hence we can reduce the result to the case of the trivial line bundle using a suitable partition of unity.

### Proof of Lemma A.4

Denote by $\mathcal {S}$ the set of conical tips, corners, and punctures of $\Omega .$ Let $\mathcal {C}_{\partial }^{\infty ,\varphi }(\Omega )$ be the set of all smooth sections of $\varphi$ that are supported away from $\mathcal {S}$ and $\partial _{\mathcal {D}}\Omega$, and which extend smoothly across $\partial _{\mathcal {N}}\Omega$ by $\phi ({\overline{x}})=\phi (x),$ where $x\mapsto {\overline{x}}$ is the reflection in ${\hat{\Omega }}$. Using a trivialization of $\varphi ,$ we can locally identify a section $\phi \in \mathcal {C}_{\partial }^{\infty ,\varphi }(\Omega )$ with a function ${\tilde{\phi }}$; expanding the latter in a Taylor series and using (10.1–10.2), it is easy to see that

$$\begin{aligned} D_{t}^{\Omega ,\varphi }\phi {\mathop {\longrightarrow }\limits ^{t\rightarrow 0}}\Delta ^{\varphi }\phi =-\frac{1}{2}(\partial _{\eta }^{2}+\partial _{\nu }^{2}){\tilde{\phi }}(x_{\eta \nu }) \end{aligned}$$

uniformly and hence in $L^{2}(\Omega ),$ where $(\eta ,\nu )\mapsto x_{\eta \nu }$ is an isometric local chart. Therefore, we have $\mathcal {C}_{\partial }^{\infty ,\varphi }(\Omega )\subset \mathcal {D}(\mathcal {E}^{\Omega ,\varphi }),$ and $\mathcal {E}^{\Omega ,\varphi }(\phi ,\phi )=\langle \phi ,\Delta ^{\varphi }\phi \rangle =\langle \nabla ^{\varphi }\phi ,\nabla ^{\varphi }\phi \rangle$ for all $\phi \in \mathcal {C}_{\partial }^{\infty ,\varphi }(\Omega ).$ Since the form $\mathcal {E}^{\Omega ,\varphi }$ is closed, this implies $H_{\partial }^{1,\varphi }(\Omega )\subset \mathcal {D}(\mathcal {E}^{\Omega ,\varphi })$ and $\mathcal {E}^{\Omega ,\varphi }(f,f)=\langle \nabla ^{\varphi }f,\nabla ^{\varphi }f\rangle ,$ $f\in H_{\partial }^{1,\varphi }(\Omega ),$ once we show that $\mathcal {C}_{\partial }^{\infty ,\varphi }(\Omega )$ is dense in $H_{\partial }^{1,\varphi }(\Omega ).$

The density is proven by a series of standard arguments. First, we can approximate any $f\in H_{\partial }^{1,\varphi }(\Omega )$ supported away from $\mathcal {S}$ and $\partial _{\mathcal {D}}\Omega$ by $\phi \in \mathcal {C}_{\partial }^{\infty ,\varphi }(\Omega ),$ by extending *f* by reflection across $\partial _{\mathcal {N}}\Omega$ and mollifying. Second, any $f\in H_{\partial }^{1,\varphi }(\Omega )$ can be approximated by bounded sections in $H_{\partial }^{1,\varphi }(\Omega ),$ by truncation at level lines (cf. [20, Example 1.2.1]): let $g_{R}(v)=\frac{Rv}{\max \{R,|v|\}},$ $v\in \mathbb {R}^{d},$ then, approximating $g_{R}$ with smooth functions and applying the chain rule, we see that $\left\| f-g_{R}f\right\| _{H^{1}}^{2}\le \int _{|f|>R}\langle \nabla ^{\varphi }f,\nabla ^{\varphi }f\rangle +\int _{|f|>R}\langle f,f\rangle \rightarrow 0$ as $R\rightarrow \infty .$ Finally, to approximate a bounded $f\in H_{\partial }^{1,\varphi }(\Omega )$ by those compactly supported away from $\mathcal {S},$ let $z_{0}\in \mathcal {S}$ and put $\phi _{\varepsilon }(z)=\max \left\{ 0,\min \left\{ 1+\frac{\log |z-z_{0}|}{-\log \epsilon },1\right\} \right\} .$ Then, $\phi _{\varepsilon }(z)\equiv 0$ for $|z-z_{0}|<\varepsilon ,$ and

$$\begin{aligned} \left\| f-\phi _{\varepsilon }\cdot f\right\| _{H^{1}}^{2}=\left\| (1-\phi _{\varepsilon })f\right\| _{L^{2}}^{2}+\left\| (1-\phi _{\varepsilon })\nabla ^{\varphi }f\right\| _{L^{2}}^{2}+\left\| f\nabla (1-\phi _{\varepsilon })\right\| _{L^{2}}^{2}. \end{aligned}$$

Since we have $0\le \phi _{\varepsilon }(z){\nearrow } 1$ as $\varepsilon \rightarrow 0,$ the first two terms above tend to zero as $\varepsilon \rightarrow 0$ by dominated convergence theorem, and $\left\| f\nabla (1-\phi _{\varepsilon })\right\| _{L^{2}}^{2}\le \sup |f|^{2}\left\| \nabla (1-\phi _{\varepsilon })\right\| _{L^{2}}^{2}\le \frac{c}{-\log \varepsilon }\rightarrow 0.$

We turn to the proof of $\mathcal {D}(\mathcal {E}^{\Omega ,\varphi })\subset H_{\partial }^{1,\varphi }(\Omega ).$ For $f\in \mathcal {D}(\mathcal {E}^{\Omega ,\varphi })$ and $\phi \in \mathcal {C}_{\partial }^{\infty ,\varphi }(\Omega )$, we have

$$\begin{aligned} \mathcal {E}^{\Omega ,\varphi }(f,\phi )=\lim _{t\rightarrow 0}\langle f,D_{t}^{\Omega ,\varphi }\phi \rangle =\langle f,\Delta ^{\varphi }\phi \rangle . \end{aligned}$$

On the other hand, by Cauchy–Schwarz,

$$\begin{aligned} \mathcal {E}^{\Omega ,\varphi }(f,\phi )\le \left( \mathcal {E}^{\Omega ,\varphi }(f,f)\right) ^{\frac{1}{2}}\left( \mathcal {E}^{\Omega ,\varphi }(\phi ,\phi )\right) ^{\frac{1}{2}}=\left( \mathcal {E}^{\Omega ,\varphi }(f,f)\right) ^{\frac{1}{2}}\langle \nabla ^{\varphi }\phi ,\nabla ^{\varphi }\phi \rangle ^{\frac{1}{2}}. \end{aligned}$$

It follows that $\phi \mapsto \langle f,\Delta ^{\varphi }\phi \rangle$ extends to a bounded linear functional on $H_{\partial }^{1,\varphi }(\Omega ),$ and thus so does $\phi \mapsto \langle f,\Delta ^{\varphi }\phi \rangle +\langle f,\phi \rangle .$ Therefore, by Riesz–Markov, there exists $\tilde{f}\in H_{\partial }^{1,\varphi }(\Omega )$ such that $\langle f,\Delta ^{\varphi }\phi \rangle +\langle f,\phi \rangle =\langle \nabla ^{\varphi }\tilde{f,}\nabla ^{\varphi }\phi \rangle +\langle \tilde{f,}\phi \rangle ,$ $\phi \in \mathcal {C}_{\partial }^{\infty ,\varphi }.$ If $\phi$ is in addition compactly supported away from $\partial \Omega ,$ the last expression is equal to $\langle \tilde{f,}\Delta ^{\varphi }\phi \rangle +\langle \tilde{f,}\phi \rangle .$ In other words, $h=f-\tilde{f}$ satisfies $\Delta ^{\varphi }h+h\equiv 0$ in the weak sense. But then, by elliptic regularity, *h* is smooth in the interior of $\Omega \setminus \mathcal {S}$; also, since $H_{\partial }^{1,\varphi }(\Omega )\subset \mathcal {D}(\mathcal {E}^{\Omega ,\varphi }),$ we have $h=f-\tilde{f}\in \mathcal {D}(\mathcal {E}^{\Omega ,\varphi }).$ Thus, it is enough to prove that if $h\in \mathcal {D}(\mathcal {E}^{\Omega ,\varphi })$ and *h* is smooth, then $h\in H_{\partial }^{1,\varphi }(\Omega ).$

We follow [45, Section 1.4]. Write

$$\begin{aligned} (P_{t}^{\Omega ,\varphi }f)(x)= & {} \mathsf {\mathbb {E}}^{x}\left[ \varphi _{\gamma _{[0,t]}}^{-1}(f(\gamma _{t}))\right] =\mathsf {\mathbb {E}}\mathsf {\mathbb {E}}^{x}\left[ \left. \varphi _{\gamma _{[0,t]}}^{-1}(f(\gamma _{t}))\right| \gamma _{t}\right] \\= & {} \int _{\Omega }R_{x,y,t}^{\Omega ,\varphi }f(y)P^{\Omega }(x,y,t)\,dy, \end{aligned}$$

where $R_{x,y,t}^{\Omega ,\varphi }f(y)=\mathsf {\mathbb {E}}^{x}\left[ \left. \varphi _{\gamma _{[0,t]}}^{-1}(f(y))\right| \gamma _{t}=y\right] .$ In the trivial bundle case, one simply has $R_{x,y,t}^{\Omega ,\varphi }f(y)=f(y).$ Due to the reversibility of $\gamma$ and the unitarity of $\varphi$, we have $\langle R_{x,y,t}^{\Omega ,\varphi }f(y),g(x)\rangle =\langle f(y),R_{y,x,t}^{\Omega ,\varphi }g(x)\rangle .$ Hence, we can write (cf. [45, Lemma 4.8])

$$\begin{aligned} \langle D_{t}^{\Omega ,\varphi }f;g\rangle =\mathcal {E}^{(1,t)}(f,g)+\mathcal {E}^{(2,t)}(f,g), \end{aligned}$$

where

$$\begin{aligned} \mathcal {E}^{(1,t)}(f,g)&=\frac{1}{2t}\int _{\Omega \times \Omega }\langle f(x)-R_{x,y,t}^{\Omega ,\varphi }f(y),g(x)-R_{x,y,t}^{\Omega ,\varphi }g(y)\rangle P^{\Omega }(x,y,t)\,dxdy,\\ \mathcal {E}^{(2,t)}(f,g)&=\frac{1}{t}\int _{\Omega }(1-Q_{t}(x))f(x)g(x)\,dx. \end{aligned}$$

and $Q_{t}(x)=\int _{\Omega }P^{\Omega }(x,y,t)dy$ is the probability that $\gamma _{t}$ started at *x* did not stop by time *t*.

For a compact set $K\subset \Omega$ and $K_{\varepsilon }$ its small neighborhood, and $h\in \mathcal {D}(\mathcal {E}^{\Omega ,\varphi })$ smooth, we have

$$\begin{aligned} \mathcal {E}^{\Omega ,\varphi }(h,h)&\ge \frac{1}{2t}\int _{K_{\varepsilon }\times K}\langle h(y)-R_{x,y,t}^{\Omega ,\varphi }h(x),h(y)-R_{x,y,t}^{\Omega ,\varphi }h(x)\rangle P^{\Omega }(x,y,t)dxdy\\&\quad {\mathop {\longrightarrow }\limits ^{t\rightarrow 0}}\int _{K}\langle \nabla ^{\varphi }h(x),\nabla ^{\varphi }h(x)\rangle \,dx, \end{aligned}$$

as is readily seen by identifying *h* with a function $\tilde{h}$ using a local trivialization near each *x* and using Taylor approximation of $\tilde{h}$ and (10.1–10.2). Taking a supremum over all such *K*, we conclude that $h\in H^{1,\varphi }(\Omega ).$

Finally, we refine $\mathcal {D}(\mathcal {E}^{\Omega ,\varphi })\subset H^{1,\varphi }(\Omega )$ to $\mathcal {D}(\mathcal {E}^{\Omega ,\varphi })\subset H_{\partial }^{1,\varphi }(\Omega ).$ We do the trivial line bundle case; the general one only differs by heavier notation. Let $0\le \phi \le 1$ be a smooth function compactly supported in a Euclidean neighborhood of $x\in \partial _{\mathcal {D}}\Omega \setminus \mathcal {S}$ and identically equal to 1 in a smaller neighborhood. If $f\in \mathcal {D}(\mathcal {E}^{\Omega }),$ then we have $\mathcal {E}^{(2,t)}(\phi f,\phi f)\le \mathcal {E}^{(2,t)}(f,f)$ and

$$\begin{aligned} \mathcal {E}^{(1,t)}(\phi f,\phi f)&=\frac{1}{2t}\int _{\Omega \times \Omega }(f(x)-f(y))^{2}\phi (x)^{2}P^{\Omega }(x,y,t)dxdy\\&\quad +\int _{\Omega }\left( \int _{\Omega }\frac{1}{2t}(\phi (x)-\phi (y))^{2}P^{\Omega }(x,y,t)dy\right) f(x)^{2}dx, \end{aligned}$$

and both terms remain bounded as $t\rightarrow 0.$ Hence, also $\phi f\in \mathcal {D}(\mathcal {E}^{\Omega }).$ Identifying $\mathrm {supp}\,\phi f$ with a subdomain of the upper half-plane $\mathbb {H}$ equipped with Dirichlet boundary conditions, due to (10.2), we have $\frac{1}{t}(P^{\Omega }(x,y,t)-P^{\mathbb {H}}(x,y,t))\rightarrow 0$ and $\frac{1}{t}(Q_{t}^{\Omega }-Q_{t}^{\mathbb {H}})\rightarrow 0$. We conclude that $\phi f\in \mathcal {D}(\mathcal {E}^{\mathbb {H}}),$ where $\mathcal {E}^{\mathbb {H}}(f,f)=\lim _{t\rightarrow 0}t^{-1}\langle D_{t}^{\mathbb {H}}f,f\rangle$. If *g* denotes $\phi f$ extended to the lower half-plane by $g({\bar{z}})=-g(z),$ then we have $P_{t}^{\mathbb {H}}(\phi f)\equiv P_{t}^{\mathbb {C}}g$ in $\mathbb {H},$ and $\mathcal {E}^{\mathbb {H}}(\phi f,\phi f)=\frac{1}{2}\mathcal {E}^{\mathbb {H}}(g,g),$ so that $g\in \mathcal {D}(\mathcal {E}^{\mathbb {C}}).$ But it is easy to see, using Fourier transform, that $\mathcal {D}(\mathcal {E}^{\mathbb {C}})=H^{1}(\mathbb {C})$ [45, Example 4.1]. If $g({\bar{z}})=-g(z),$ then we can only have $g\in H^{1}(\mathbb {C})$ if $g\equiv 0$ a.e. on $\mathbb {R}.$ We conclude that $f\equiv 0$ a.e. on $\partial _{\mathcal {D}}\Omega .$ $\square$
